# Telemedicine interventions for improving antibiotic stewardship and prescribing: A systematic review

**DOI:** 10.1371/journal.pone.0320840

**Published:** 2025-04-03

**Authors:** Ghasem Dolatkhah Laein, Javad Raeesi, Ali Mokhtari, Omid Salehinia, Mohammad Mehri, Ujjwal Shilanath Tiwary

**Affiliations:** 1 Mashhad University of Medical Sciences, Mashhad, Iran; 2 Health and Rehabilitation Sciences, The University of Western Ontario, London, Ontario, Canada; 3 Department of Biology, Faculty of Science, Islamic Azad University of Mashhad, Mashhad, Iran; 4 Department of Electrical and Electronics Engineering, Ferdowsi University of Mashhad, Mashhad, Iran; 5 Semnan University of Medical Sciences, Semnan, Iran; 6 Rajiv Gandhi University of Health Sciences, Bengaluru, Karnataka, India; UT Southwestern Medical Center, UNITED STATES OF AMERICA

## Abstract

The global antibiotic resistance crisis necessitates optimized stewardship programs, with telemedicine emerging as a promising delivery strategy. This systematic review evaluated the effectiveness of telemedicine interventions in improving antibiotic stewardship across clinical settings. Following Preferred Reporting Items for Systematic Reviews and Meta-Analyses (PRISMA) guidelines, we systematically searched seven databases from January 2010 to July 2024. Two independent reviewers assessed studies using Risk of Bias in Non-randomized Studies (ROBINS-I) and Cochrane Risk of Bias 2.0 tools, with evidence certainty evaluated using Grading of Recommendations Assessment, Development, and Evaluation (GRADE). Twenty-one studies met inclusion criteria (10 observational, 8 quasi-experimental, 2 Randomized Controlled Trials [RCTs], 1 mixed-methods), predominantly from the United States (57.0%, n = 12). Among studies reporting antibiotic use outcomes (52.4%, n = 11), 90.9% demonstrated significant reductions ranging from 5.3% to 62.7%, with the highest-quality evidence showing a 28% reduction (95% Confidence Interval [CI]: 22-34%). Guideline adherence studies (57.1%, n = 12) showed acceptance rates of 67.7% to 98%, with comparable effectiveness between telemedicine and in-person consultation (79.1% vs 80.4%, p = 0.36). Prescribing rate outcomes (38.1%, n = 8) revealed setting-dependent variations: inpatient implementations demonstrated significant reductions (Relative Risk [RR] 0.68; 95% CI: 0.63-0.75), while outpatient findings showed mixed results. Quality assessment revealed predominantly low risk of bias [ROB] (66.7%, n = 14). These findings suggest that telemedicine effectively improves antibiotic stewardship compared to traditional care models, particularly in hospital settings, while outpatient applications demonstrated variable effectiveness. This review was registered with the International Prospective Register of Systematic Reviews (PROSPERO: CRD42023454663).

## Introduction

The global antibiotic resistance crisis poses a significant threat to public health worldwide [1–6]. Up to 50% of hospital antibiotic use is unnecessary or inappropriate [7–10], emphasizing the critical need for optimized antibiotic stewardship programs. Antibiotic resistance occurs when bacteria change their genetics, making antibiotics no longer able to kill or inhibit their growth [11,12]. It can also result from incorrect or excessive antibiotic use, leading to natural selection and the evolution of resistant bacteria [13,14].

One of the main reasons for antibiotic resistance is the unnecessary and inappropriate prescription of antibiotics by doctors [15]. Antibiotics are often prescribed for viral infections that do not naturally respond to them [16]. This problem not only negatively affects the treatment of the disease but also increases the selective pressure for the creation and spread of resistant bacteria [16]. Additionally, patients’ nonadherence to the prescribed antibiotic dose and duration is another important factor contributing to resistance [17].

Another factor contributing to antibiotic resistance is the widespread use of antibiotics in livestock and agriculture [18,19]. Antibiotics are widely used to stimulate growth and prevent animal disease [20,21]. This widespread use can lead to the transfer of resistant bacteria to humans through the food chain. Furthermore, environmental pollution with antibiotics and their residues in water and soil sources can lead to resistance to environmental bacteria, which can then be transferred to humans and animals [22–24].

Telemedicine technologies, such as video consultations, clinical decision support tools, and remote monitoring, have emerged as promising strategies to implement and deliver antibiotic stewardship initiatives, a concept known as telestewardship [25–28]. Previous studies have highlighted the potential of telemedicine technologies to enhance the management of infectious diseases and improve antibiotic prescribing practices [29–32].

The Infectious Diseases Society of America has also recognized the role of telehealth and telemedicine in the practice of infectious diseases, including its application in antimicrobial stewardship programs [33]. Furthermore, previous studies have demonstrated the successful use of telemedicine videoconference consultations for managing infectious diseases in remote settings, showcasing the potential of telestewardship to promote judicious antibiotic use in resource-limited areas [34–39]. Telestewardship could provide a scalable way to enhance stewardship through prospective review, education, and point-of-care decision support [40–44].

However, current evidence regarding the effectiveness of telestewardship in improving prescribing practices across different clinical settings remains limited. The present systematic review aims to provide a more comprehensive, up-to-date synthesis of the impacts of diverse telestewardship interventions across various clinical settings. By examining a broader range of telemedicine modalities and contexts, our findings may help guide policies and guidelines for optimized telestewardship programs in global healthcare systems.

## Methods

This systematic review adhered to PRISMA guidelines for transparent and complete reporting [45]. The current review protocol was registered in advance with PROSPERO, registration number CRD42023454663. This literature review did not involve research on human subjects and did not require ethics approval. Literature searching and access were solely through public databases and resources. The GRADE approach was employed to evaluate the overall certainty of the evidence [46]. Furthermore, we used the Cochrane Handbook for Systematic Reviews of Interventions as a reference throughout the review process [47].

### Search strategy

The search was conducted to identify relevant studies published from January 1, 2010, to July 1, 2024. The databases and platforms were searched, including PubMed, Embase (Ovid), Cochrane Central Register of Controlled Trials (Wiley), CINAHL (EBSCO), Web of Science (Clarivate), PsycINFO (EBSCO), and Google Scholar. The search strategy was developed by a research librarian (AM). It included a combination of keywords, medical subject headings (MeSH), and free text terms related to concepts of telemedicine, antibiotic stewardship, and antibiotic use. The Full details of search strategies are provided in S1 Appendix. The references to the included studies and relevant reviews were also hand-searched. EndNote 21.0 citation management software organized references throughout the review process.

### Study selection

Two reviewers independently conducted literature screening and selection using Covidence systematic review management software [48,49]. The screening was performed in 2 stages - titles/abstracts and then full texts. Studies were included if they met the following criteria: (1) assessed telemedicine interventions aimed at implementing or delivering antibiotic stewardship programs (telestewardship); (2) included a comparator receiving usual care without telemedicine; (3) reported quantitative outcome measures related to antibiotic use or resistance; (4) published between January 2010 to the first of July 2024 in English. Reviews, protocols, editorials, and qualitative studies were excluded. Based on these initial selection criteria, The current study further refined study selection using the following detailed inclusion and exclusion criteria to ensure a comprehensive and relevant analysis of the current evidence in telemedicine for antibiotic stewardship.

### Inclusion criteria

1) Population: Patients receiving care for infectious diseases or conditions that may require antibiotic treatment, with no restrictions on age, gender, or comorbidities.
2) Interventions: Telemedicine programs (e.g., video consultations, remote monitoring, clinical decision support systems) aimed at improving antibiotic stewardship or reducing antibiotic prescribing, delivered by licensed healthcare professionals (e.g., physicians, nurse practitioners, physician assistants).
3) Comparators: In-person care or standard antibiotic prescribing practices without telemedicine components.
4) Outcomes: Quantitative measures related to antibiotic prescribing patterns, antibiotic appropriateness, guideline adherence, and resistance rates.
5) Study Designs: Randomized controlled trials and observational studies, including quasi-experimental, before-after, cohort, case-control, and cross-sectional studies.
6) Language: English.
7) Years: January 1, 2010 to July 1, 2024.

### Exclusion criteria

1) Study Designs: Reviews, protocols, editorials, opinion pieces
2) Outcomes: Qualitative outcomes only
3) Language: Non-English publications

### Definition of terms

1) Inpatient Telestewardship: Telemedicine interventions for hospitalized patients, typically involving remote infectious disease consultations, virtual rounds, or decision-support tools aimed at optimizing antibiotic therapy in real-time.
2) Outpatient Telemedicine Stewardship: Telemedicine-based programs implemented in ambulatory or primary care settings (e.g., clinics, telemedicine platforms) to guide antibiotic prescriptions and reduce inappropriate antibiotic use outside the hospital setting.
3) Comparator Arm: For included studies, the comparator had no telemedicine component. This could be usual care with or without an existing (in-person) antimicrobial stewardship program, as reported by each study.
4) Antibiotic Appropriateness: Defined as selecting the correct agent, dose, route, and duration in line with recognized local or international treatment guidelines.

### Data extraction

A standardized data extraction process was undertaken by two independent reviewers to gather relevant details from the included studies in a consistent manner. Microsoft Excel created a predetermined form to capture key data points on study identification, population, interventions, comparators, measured outcomes, statistical analyses, and confounding factors. Specifically, extracted information included author, publication year, study design, location, sample size, participant demographics, healthcare setting characteristics, types of telemedicine modalities, duration of interventions, details on usual care comparators, antibiotic prescribing, and appropriateness parameters, effect sizes with statistical significance values, and factors adjusted for in the analysis. Any disagreements between the two primary reviewers were resolved through discussion and consensus; if consensus could not be reached, the disagreement was elevated to a third reviewer to make a final determination. The senior author thus did not unilaterally decide outcomes when the primary reviewers’ consensus was lacking, ensuring impartial resolution of conflicts.

### Risk of bias assessment

Two independent reviewers assessed the risk of bias for each included study. For randomized controlled trials, the Cochrane Risk of Bias tool 2.0 [49] and non-randomized studies, the ROBINS-I [50] tool was applied to assess the risk of bias due to confounding, selection, classification, deviation from interventions, missing data, measurement of outcomes, and selection of the reported results. Any disagreements in risk of bias judgments were resolved through discussion between the two reviewers to reach a consensus. The overall risk of bias was categorized as low, moderate, or high for each study based on criteria and domains outlined in the Cochrane and ROBINS-I tools. The assessment process was tailored to judge the biases most applicable to the study design. Additionally, we evaluated the certainty of evidence for each outcome using the GRADE approach [51], considering factors such as risk of bias, inconsistency, indirectness, imprecision, and publication bias.

### Data synthesis

Due to the range of different telemedicine interventions and outcome measures reported across studies, a structured narrative synthesis was performed. Where sufficient clinical and methodological homogeneity existed, summary estimates were pooled using random-effects meta-analysis. All analyses were conducted using Review Manager software (RevMan) [52].

## Results

**Fig 1:**
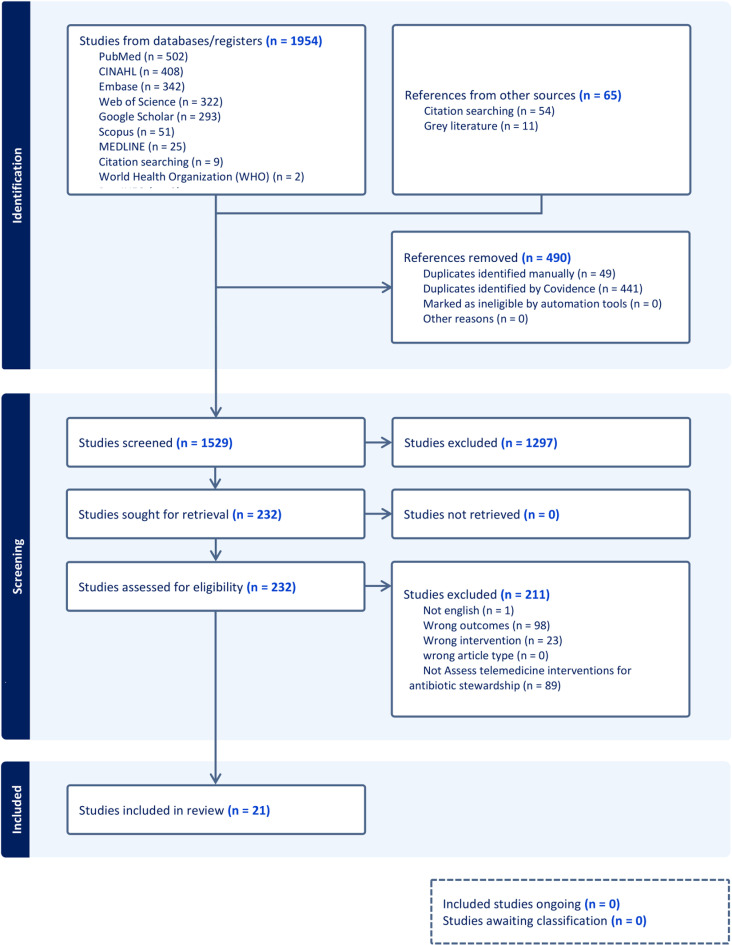
PRISMA flow diagram.

A total of 2019 references were imported for screening. After 490 duplicate records were removed, 1529 studies were screened based on title and abstract. This screening excluded 1297 studies, leaving 232 studies to assess full-text eligibility. Of these, 211 studies were excluded based on the predefined criteria. No ongoing studies or studies awaiting classification were identified. Ultimately, 21 studies fulfilled the inclusion criteria and were incorporated into the systematic review. Complete documentation of exclusion rationales provided in S2 Appendix. The PRISMA checklist is provided in S3 Appendix, and the study selection process is illustrated in the PRISMA flow diagram (Fig 1).

### Study characteristics

Of the 21 included studies [53–73], observational studies constituted 47.6% (n = 10) [53,55,56,58,59,62,63,67–69], followed by quasi-experimental studies 38.1% (n = 8) [54,57,60,61,70–73], randomized controlled trials 9.5% (n = 2) [64,65], and mixed-methods studies 4.8% (n = 1) [66]. Studies were predominantly conducted in the United States 57.0% (n = 12) [54,55,57,61,64–71], with representation from the United Kingdom 14.3% (n = 3) [59,63,72], Brazil 9.5% (n = 2) [53,58], and individual contributions 4.8% (n = 1) each from France [56], Australia [60], Kuwait [62], and Canada [73]. Hospital-based interventions dominated at 71.4% (n = 15) [53–56,60–63,65–67,70–73], while primary care settings represented 23.8% (n = 5) [57,59,64,68,69] and telemedicine centers 4.8% (n = 1) [58]. Regarding intervention comparisons, 71.4% (n = 15) evaluated telemedicine against no formal stewardship service [53–58,61–63,65,66,70–73], while 28.6% (n = 6) compared telemedicine with in-person care [59,60,64,67–69]. Study duration ranged from 3 months [62] to 84 months [72,73], with a median of 18 months. Studies were published between 2015 and 2024, with most conducted during 2019–2024 [54,55,58–68,70,71]. Population sizes showed considerable variation, ranging from 20 beds [62] to 221,128 patients [58], precluding standardized comparison. A summary of study characteristics is presented in Table 1.

**Table 1:** Summary of study characteristics.

Authors	Study Design	Country	Population Size	Care Setting	Telestewardship_Type	Comparison	Comparison Detail	Outcome
Tuon et al. 2017 [53]	Observational	Brazil	186 beds (37 ICU)	Inpatient	Provider-Provider	No Service	Before-After: 12-month pre vs. 12-month post implementation	Antibiotic Use
Shively et al. 2019 [54]	Quasi-Experimental	USA	461 beds total	Inpatient	Provider-Provider	No Service	Before-After: Pre-intervention period with no formal ASP	Antibiotic Use, Guideline Adherence
Vento et al. 2021 [55]	Observational	USA	688 beds (16 hospitals)	Inpatient	Provider-Provider	No Service	Before-After: Pre-implementation period without telehealth services	Antibiotic Use, Guideline Adherence
Morquin et al. 2015 [56]	Observational	France	2500 beds	Inpatient	Provider-Provider	No Service	Before-After: Traditional consultation (implicit comparison)	Guideline Adherence
Grabinski et al. 2024 [57]	Quasi-Experimental	USA	133804 visits	Outpatient	Provider-Provider	No Service	Before-After: Standard telemedicine care without care bundle	Prescribing Rates
Moreira et al. 2024 [58]	Observational	Brazil	221128 patients	Outpatient	Provider-Provider	No Service	Before-After: Telemedicine-only intervention	Prescribing Rates
Vestesson et al. 2023 [59]	Observational	UK	45997 consultations	Outpatient	Patient-Provider	In-Person	Direct Comparison: Face-to-face consultations	Prescribing Rates
Bazargani et al. 2022 [60]	Quasi-Experimental	Australia	783 beds	Inpatient	Patient-Provider	In-Person	Direct Comparison: Face-to-face PAF rounds	Guideline Adherence
May et al. 2023 [61]	Quasi-Experimental	USA	1815 patients	Inpatient	Provider-Provider	No Service	Before-After: Pre-intervention standard practice	Antibiotic Use, Prescribing Rates
Alfraij et al. 2023 [62]	Observational	Kuwait	20 beds	Inpatient	Provider-Provider	No Service	Before-After: Pre-implementation period without ASP	Antibiotic Use, Guideline Adherence
Heard et al. 2019 [63]	Observational	UK	450 beds	Inpatient	Provider-Provider	No Service	Before-After: Pre-CDSS manual workflow	Antibiotic Use, Guideline Adherence
Du Yan et al. 2021 [64]	RCT(Randomized Controlled Trial)	USA	45 clinicians	Outpatient	Patient-Provider	In-Person	Direct Comparison: Education alone	Prescribing Rates
Cantey et al. 2022 [65]	RCT	USA	9277 infants	Inpatient	Provider-Provider	No Service	Before-After: Pre-intervention period without ASP	Antibiotic Use, Prescribing Rates
Livorsi et al. 2023 [66]	Mixed-Methods	USA	502 patients	Inpatient	Provider-Provider	No Service	Before-After: Pre-intervention without ID support	Antibiotic Use, Guideline Adherence
Meredith et al. 2021 [67]	Observational	USA	738 patients	Inpatient	Patient-Provider	In-Person	Direct Comparison: On-site ID consultation	Guideline Adherence
Ray et al. 2021 [68]	Observational	USA	47 practices	Outpatient	Patient-Provider	In-Person	Direct Comparison: In-person office visits	Guideline Adherence, Prescribing Rates
Davis et al. 2018 [69]	Observational	USA	157 patients	Outpatient	Patient-Provider	In-Person	Direct Comparison: Traditional urgent care	Prescribing Rates
Wilson et al. 2019 [70]	Quasi-Experimental	USA	2 centers	Inpatient	Provider-Provider	No Service	Before-After: Pre-intervention standard practice	Antibiotic Use
Klatt et al. 2021 [71]	Quasi-Experimental	USA	95 beds	Inpatient	Provider-Provider	No Service	Before-After: Independent local stewardship activities	Antibiotic Use, Guideline Adherence
Charani et al. 2017 [72]	Quasi-Experimental	UK	1300 beds total	Inpatient	Provider-Provider	No Service	Before-After: Standard ASP practices	Guideline Adherence
Nault et al.2016 [73]	Quasi-Experimental	Canada	40605 hospitalizations	Inpatient	Provider-Provider	No Service	Before-After: Pre-intervention without systematic ASP	Antibiotic Use, Guideline Adherence

### Quality assessment and risk of bias

**Fig 2:**
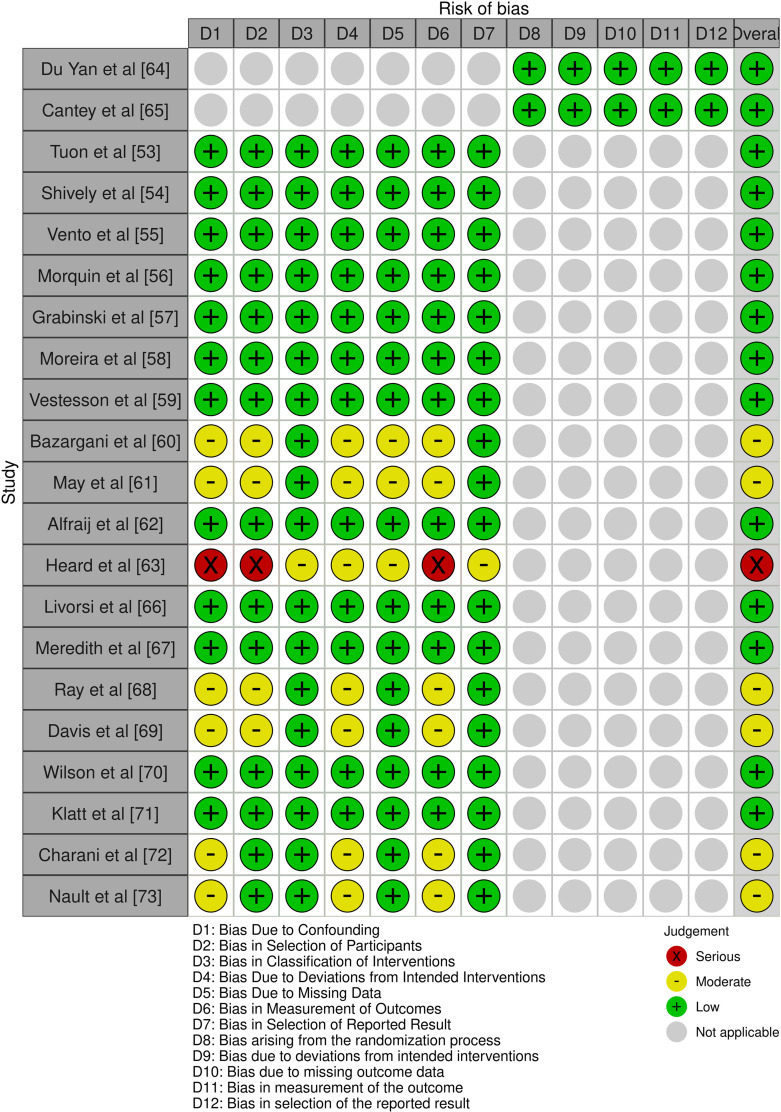
Risk of bias assessment.

The quality evaluation encompassed all studies, utilizing the ROBINS-I tool for 90.5% (n = 19) studies [53–63,66–73] and the Cochrane Risk of Bias 2.0 tool for 9.5% (n = 2) studies [64,65] (Fig 2). Overall risk assessment revealed 66.7% (n = 14) studies demonstrating low risk [53–59,62,64–67,70,71], 28.6% (n = 6) studies showing moderate risk [60,61,68,69,72,73], and 4.8% (n = 1) study exhibiting high risk [63]. The predominant factors contributing to elevated risk included potential confounding and selection bias, while the randomized controlled trials [64,65] demonstrated robust methodological quality. The GRADE assessment revealed 9.5% (n = 2) studies of high quality [64,65], 76.2% (n = 16) studies of moderate quality [55–63,66,68–73], and 14.3% (n = 3) studies of low quality [53,54,67]. A detailed risk of bias assessment for individual studies is presented in S4 Appendix. Table 2 presents the GRADE Summary of Findings for quality assessment of outcomes.

**Table 2:** GRADE summary of findings.

Outcomes	Number of Participants	Study Design	Risk of Bias	Inconsistency	Indirectness	Imprecision	Publication Bias	Effect Estimates	Overall GRADE Rating
**Impact on Antibiotic Use**	11 [53–55,61–63,65,66,70,71,73]	1 RCT (High GRADE) [65]. 8 Non-RCTs (Moderate GRADE) [55,61–63,66,70,71,73]. 2 Non-RCTs (Low GRADE) [53,54].	○ SERIOUS 1 Serious risk study [63]. 2 Moderate risk studies [61,73]. 8 Low risk studies [53–55,62,65,66,70,71].	⊕ NOT SERIOUS Consistent direction in 10/11 studies. One mixed result [55]. Aligned effect sizes.	⊕ NOT SERIOUS Direct population relevance. Appropriate interventions. Relevant outcomes	⊕ NOT SERIOUS Adequate sample sizes. Narrow Cis. Clear effects	⊕ NOT DETECTED No selective reporting Various effect sizes Multiple funding sources	Range: 5.3-62.7% reduction. High-quality evidence: 28% reduction (95% CI: 22-34%)	⊕⊕⊕○ MODERATE¹
**Impact on Guideline Adherence**	12 [54–56,60,62,63,66–68,71–73].	9 Non-RCTs (Moderate GRADE) [55,56,60,62,63,66,68,71,72]. 3 Non-RCTs (Low GRADE) [54,67,73]	○ SERIOUS 1 Serious risk [63]. 3 Moderate risk [60,68,72]. 8 Low risk [54–56,62,66,67,71,73].	⊕ NOT SERIOUS Consistent improvements. Similar magnitude. Comparable results.	⊕ NOT SERIOUS Direct applicability. Clear pathway. Relevant outcomes	⊕ NOT SERIOUS Sufficient samples. Precise estimates. Clear significance	⊕ NOT DETECTED No selective reporting. Range of outcomes. Diverse origins	Range: 67.7-98% adherence. Superiority demonstrated (92.5% vs 90.7%, p = 0.004)	⊕⊕⊕○ MODERATE¹
**Impact on Prescribing Rates**	8 [57–59,61,64,65,68,69].	2 RCTs (High GRADE) [64,65]. 6 Non-RCTs (Moderate GRADE) [57–59,61,68,69].	○ MODERATE 3 Moderate risk [61,68,69]. 5 Low risk studies [57–59,64,65].	○ SERIOUS Conflicting directions. Mixed effects. Variable magnitudes.	⊕ NOT SERIOUS Appropriate population. Direct comparison. Relevant outcomes	⊕ NOT SERIOUS Adequate samples. Precise CIs. Clear effects	⊕ NOT DETECTED No selective reporting. Both positive/negative results. Various funding	No antibiotics: 28% increase. Infant prescriptions: 32% reduction (RR = 0.68; 95% CI: 0.63-0.75)	⊕⊕⊕○ MODERATE¹

**Notes**:

¹ GRADE Rating Definitions: ⊕⊕⊕⊕ HIGH: Further research very unlikely to change confidence in effect estimate. ⊕⊕⊕○ MODERATE: Further research likely to impact confidence in effect estimate. ⊕⊕○○ LOW: Further research very likely to impact confidence in effect estimate. ⊕ ○○○ VERY LOW: Any estimate of effect is very uncertain.

### Synthesis of results

Meta-analysis was not conducted across the 21 included studies [53–73] due to substantial methodological heterogeneity in intervention modalities, comparator groups, and outcome metrics (Cochrane Handbook, Section 10.10.2) [47]. We implemented a structured descriptive synthesis using an outcomes-first approach, stratifying evidence by primary outcomes (antibiotic utilization, guideline adherence, prescribing rates), comparator types (telemedicine vs. no service; telemedicine vs. in-person care), and clinical settings (inpatient/outpatient). Quality assessment was integrated at each analytical level, with findings weighted by risk of bias assessments and maintaining a minimum threshold of three studies per subgroup to ensure reliable conclusions. The complete dataset for all 21 included studies is available in S5 Appendix.

### Impact on antibiotic use

Of the twenty-one included studies, 52.4% (n = 11) reported antibiotic use outcomes [53–55,61–63,65,66,70,71,73], all conducted in inpatient settings comparing telemedicine against no formal stewardship service. Significant reductions in antibiotic use were reported in 90.9% (n = 10) studies [53,54,61–63,65,66,70,71,73], with magnitudes ranging from 5.3% [54] to 62.7% [71]. The highest-quality evidence [65] demonstrated a 28% reduction (95% CI: 22-34%) in total antibiotic consumption. Notable findings included decreased broad-spectrum antibiotic use [53,54], with specific reductions in carbapenems (59%) [54] and fluoroquinolones (35%) [54]. One study reported mixed results, showing decreases in some antibiotics while others increased [55]. The evidence quality distribution showed 9.1% (n = 1) high-GRADE [65], 18.2% (n = 2) low-GRADE [53,54], and 72.7% (n = 8) moderate-GRADE studies [55,61–63,66,70,71,73]. Risk of bias was predominantly low in 72.7% (n = 8) studies [53–55,62,65,66,70,71], with 18.2% (n = 2) showing moderate risk [61,73], and 9.1% (n = 1) exhibiting Serious risk [63].

### Impact on guideline adherence

Among the twenty-one included studies, 57.1% (n = 12) reported guideline adherence outcomes [54–56,60,62,63,66–68,71–73]. Distinct patterns emerged across settings and comparators. For telemedicine versus no service, 42.9% (n = 9) studies [54–56,62,63,66,71–73], all in inpatient settings, demonstrated consistent improvements. Acceptance rates ranged from 67.7% [71] to 98% [55,63], with comprehensive adherence metrics showing significant enhancements. Full compliance rates varied from 79% (95% CI 76.4-81.6%) for diagnostic recommendations [56] to 89.7% for overall interventions [62]. Policy adherence improved by 6.48-6.63% across medical and surgical services [72], with documentation compliance increasing by 15.20-35.97% [72]. For telemedicine versus in-person consultation, 14.3% (n = 3) studies provided evidence [60,67,68]. In the inpatient setting, two studies revealed comparable adherence rates between modalities (79.1% vs 80.4%, p = 0.36 [60]; 89% vs 86%, p = 0.33 [67]). The single outpatient study demonstrated marginally superior guideline concordance with telemedicine (92.5% vs 90.7%, p = 0.004) [68]. Evidence quality assessment revealed 75.0% (n = 9) moderate-GRADE studies [55,56,60,62,63,66,68,71,72] and 25.0% (n = 3) low-GRADE studies [54,67,73]. Risk of bias was predominantly low in 66.7% (n = 8) studies [54–56,62,66,67,71,73], with 25.0% (n = 3) showing moderate risk [60,68,72], and 8.3% (n = 1) exhibiting Serious risk [63].

### Impact on prescribing rates

Among the twenty-one included studies, 38.1% (n = 8) reported prescribing rate outcomes [57–59,61,64,65,68,69]. Analysis revealed distinct patterns across settings and comparators. For telemedicine versus no service, 19.0% (n = 4) studies provided evidence [57,58,61,65], equally distributed between inpatient and outpatient settings. In the inpatient setting, both studies demonstrated significant reductions: a 28% increase in patients receiving no antibiotics (p < 0.0001) [61] and a 32% reduction in infant antibiotic prescriptions (RR 0.68; 95% CI 0.63-0.75) [65]. The outpatient studies showed modest improvements, with one reporting a 3.9% absolute reduction in sinusitis prescribing (p < 0.001) [57] and another maintaining consistently low prescribing rates for COVID-19 cases [58]. For telemedicine versus in-person consultation, 19.0% (n = 4) studies, all in outpatient settings [59,64,68,69], revealed mixed outcomes. Adult telemedicine prescribing showed higher rates compared to face-to-face care (52% vs 42%; OR 1.23; 95% CI 1.18-1.29) [59], while three studies demonstrated favorable reductions in prescribing rates [64,68,69], particularly for respiratory conditions. Evidence quality assessment revealed 25.0% (n = 2) high-GRADE studies [64,65] and 75.0% (n = 6) moderate-GRADE studies [57–59,61,68,69]. Risk of bias was predominantly low in 62.5% (n = 5) studies [57–59,64,65], with 37.5% (n = 3) showing moderate risk [61,68,69].

### Sensitivity analysis and quality assessment impact

A detailed comparison of main and sensitivity analyses, including separate exclusion criteria for severe-ROB and low-GRADE studies, is provided in Table 3, demonstrating that our core findings remained largely unchanged across all analytical scenarios. Our methodological quality assessment revealed 4.8% (n = 1) Serious risk of bias [63] and 14.3% (n = 3) low-GRADE evidence [53,54,67] among twenty-one studies. Quality-adjusted sensitivity analysis demonstrated robust findings across outcomes. For antibiotic use, excluding lower-quality evidence [53,54,63] maintained consistent effect sizes among 72.7% (n = 8) studies, with reductions ranging from 28% [65] to 62.7% [71] (p < 0.05). Prescribing rates outcomes, after excluding one low-GRADE study [67], retained statistical significance (p < 0.001) across 87.5% (n = 7) studies [57,59,64,68,69]. Similarly, guideline adherence outcomes remained stable after removing three lower-quality studies [54,63,67], with acceptance rates among 75.0% (n = 9) studies ranging from 67.7% [71] to 89.7% [62] (p < 0.05). This sensitivity analysis, employing a standardized quality-adjustment framework (Initial Studies - (Serious ROB +  Low GRADE) =  Adjusted Sample), confirms the resilience of our primary findings to methodological limitations across all outcomes, particularly supported by high-GRADE evidence [64,65].

**Table 3:** Main analysis versus sensitivity analysis results.

Outcome	Main Analysis	ROB Sensitivity Analysis^¹^	GRADE Sensitivity Analysis^2^	Combined Sensitivity Analysis^3^	Impact Assessment		
					ROB	GRADE	Combine
**Antibiotic Use**	n = 11 [53–55,61–63,65,66,70,71,73] Range: 5.3%-62.7%	n = 10 [53–55,61,62,65,66,70,71,73] Range: 5.3%-62.7%	n = 9 [55,61–63,65,66,70,71,73] Range: 28%-62.7%	n = 8 [55,61,62,65,66,70,71,73] Range: 28%-62.7%	No significant impact	Narrowed effect range	Maintained core findings
**Prescribing Rates**	n = 8 [57–59,61,64,65,68,69] p < 0.001	n = 8 [57–59,61,64,65,68,69] p < 0.001	n = 7 [57–59,61,64,65,68,69] p < 0.001	n = 7 [57–59,61,64,65,68,69] p < 0.001	No studies with Serious ROB	No impact on significance	Findings remained robust
**Guideline Adherence**	n = 12 [54–56,60,62,63,66–68,71–73] Range: 67.7%-98%	n = 11 [54,55,60,62,66–68,71–73] Range: 67.7%-89.7%	n = 10 [55,56,60,62,63,66,68,71–73] Range: 67.7%-98%	n = 9 [55,56,60,62,66,68,71–73] Range: 67.7%-89.7%	Reduced upper range	Minimal impact	Maintained core acceptance rates

**Notes:**

¹ Excluding Serious ROB study [63],

^2^Excluding low-GRADE studies [53,54,67],

^3^Excluding both Serious ROB [63] and low-GRADE studies [53,54,67].

## Discussion

Antimicrobial resistance represents a critical global health challenge, with inappropriate antibiotic use contributing significantly to this growing threat [74]. As healthcare systems increasingly adopt digital solutions [75], telemedicine emerges as a promising tool for expanding antimicrobial stewardship programs, particularly in resource-limited settings [76]. This systematic review synthesizes evidence from 21 studies (2015-2024) evaluating telemedicine-based antibiotic stewardship interventions, revealing differentiated effectiveness across healthcare settings [53–73]. Analysis of antibiotic utilization outcomes (n = 11 studies) demonstrated consistent improvements, with 90.9% of studies reporting significant reductions (range: 5.3%-62.7%), supported by high-quality randomized controlled trial evidence (28% reduction; 95% CI: 22-34%) [65]. Guideline adherence data from twelve studies showed robust implementation success (acceptance rates: 67.7%-98%) [55,63,71] and comparable effectiveness between telemedicine and in-person consultation (79.1% vs 80.4%, p = 0.36) [60]. Prescribing patterns revealed setting-dependent associations: inpatient implementations showed significant reductions in antibiotic prescriptions (RR 0.68; 95% CI 0.63-0.75) [65], while outpatient outcomes demonstrated more variable effects. The methodological quality of these findings is supported by low risk of bias in 66.7% of studies [53–59,62,64–67,70,71]. Secondary outcome analysis from six inpatient studies comparing telemedicine versus no service demonstrated consistent economic benefits, with annual cost reductions ranging from USD (United States Dollar) 142,629 to USD 350,000 [53,54,73], and operational improvements including reduced antibiotic days [61] and streamlined workflow processes [55]. In the context of increasing healthcare digitalization and the global imperative to optimize antimicrobial use [77], these findings suggest that telemedicine-based interventions may effectively support antibiotic stewardship, particularly in inpatient environments, while highlighting the need for setting-specific optimization strategies in ambulatory care.

### Comparison with existing literature

Our systematic review extends the evidence base established by previous analyses. Nathwani et al. [78] demonstrated comparable effectiveness in traditional stewardship programs, with our inpatient antibiotic reduction rates (5.3%-62.7%) aligning with their reported outcomes (0.06%-80.1%). Bakhit et al.’s [79] analysis of 13 studies revealed condition-specific variations in outpatient prescribing patterns, contrasting with our inpatient findings where high-quality RCT evidence demonstrated consistent improvements (RR 0.68; 95% CI 0.63-0.75) [65]. While Dyar et al.’s [80] framework established comprehensive prescribing competencies through expert consensus (98% agreement, 24 countries), our review provides specific evidence for implementation effectiveness through guideline adherence rates (67.7%-98%) [55,63,71]. The comparable effectiveness between telemedicine and in-person consultation (79.1% vs 80.4%, p = 0.36) [60] suggests successful adaptation of stewardship practices to remote delivery models.

### Strengths and limitations

The review’s strength lies in its comprehensive examination of telemedicine-based interventions during healthcare digitalization (2015-2024), with robust methodology and quality assessment. The predominantly low risk of bias [53–59,62,64–67,70,71] and substantial proportion of moderate GRADE quality studies enhance reliability. However, outcome heterogeneity precluded meta-analysis, and limited outpatient evidence restricts generalizability. Variable study durations may not fully capture long-term intervention sustainability.

### Implications for practice and policy

The evidence supports specific considerations for telemedicine-based antibiotic stewardship implementation. In inpatient settings, our analysis demonstrates both clinical and economic benefits. Six studies comparing telemedicine versus no service [53–55,61,62,73] reported consistent cost reductions, ranging from USD 4,690 to USD 350,000 annually, with one study showing 20.5% reduction in antimicrobial budget [73]. Implementation effectiveness is supported by high guideline adherence rates (67.7-98%) [55,63,71] and equivalent outcomes between telemedicine and in-person consultation (79.1% vs 80.4%, p = 0.36) [60]. Operational improvements include reduced antibiotic days [61] and streamlined workflows [55], suggesting feasible integration into existing healthcare systems. The evidence particularly supports inpatient applications, where systematic monitoring demonstrated significant reductions in antibiotic consumption (range: 5.3%-62.7%) [53,54,61–63,65,66,70,71,73].

### Implications for practice and policy

In the context of global antimicrobial resistance challenges and increasing healthcare digitalization, this systematic review provides evidence-based support for telemedicine as a viable solution for expanding antibiotic stewardship programs. The demonstrated effectiveness in inpatient settings, combining both clinical improvements [53,54,61–63,65,66,70,71,73] and economic benefits [53,54,73], suggests telemedicine offers a scalable model for healthcare systems struggling with antimicrobial resistance. The comparable effectiveness between telemedicine and traditional consultation methods [60] indicates potential for widespread implementation, particularly valuable for resource-limited settings lacking on-site infectious disease specialists. However, the variable effectiveness in outpatient settings highlights the need for context-specific implementation strategies. These findings provide timely evidence for healthcare policymakers and administrators considering digital solutions to enhance antimicrobial stewardship programs, especially given the growing global emphasis on combating antibiotic resistance.

### Future research directions

Future research should address three critical methodological gaps identified in this systematic review. First, standardization of outcome measures for antibiotic stewardship interventions is needed to enable meta-analyses and strengthen the evidence base, as current heterogeneity in metrics precluded quantitative synthesis. Second, while our review found promising evidence for telemedicine-based stewardship, the limited number of randomized controlled trials [64,65] indicates the need for more robust experimental designs, particularly comparative effectiveness studies between telemedicine and traditional in-person services. Third, to inform implementation strategies, future trials should incorporate standardized economic analyses, as current evidence of cost-effectiveness is limited and inconsistently reported across studies [53–55,61,62,73].

## Conclusion

This systematic review demonstrates that telemedicine interventions effectively improve antibiotic stewardship compared to traditional care models, with strongest evidence in inpatient settings. While hospital-based implementations showed consistent benefits in antibiotic utilization and guideline adherence, outpatient applications demonstrated variable effectiveness. The evidence supports telemedicine as a viable approach for expanding antimicrobial stewardship programs, particularly in hospital environments.

## Supporting information

S1 Appendix Full search strategy. A detailed description of the search strategy used for each database.
(PDF)

S2 Appendix Documentation of exclusion rationales. (CSV)

S3 Appendix PRISMA checklist. Completed PRISMA checklist for the systematic review.
(PDF)

S4 Appendix A detailed risk of bias assessment for individual studies. (DOCX)

S5 Appendix Full extracted raw data. (CSV)
