# Supplementary material for: Telemedicine interventions for improving antibiotic stewardship and prescribing: A systematic review
Source: PLoS One. 2025 Apr 3;20(4):e0320840. doi: 10.1371/journal.pone.0320840 (PMC11967954; doi:10.1371/journal.pone.0320840)
Supplement: S1 Appendix — A detailed description of the search strategy used for each database. (PDF) [file pone.0320840.s001.pdf]

Appendix 1. The full search strategies for each database

PubMed

(("Telemedicine"[Mesh]) OR telemedicine[tiab] OR telehealth[tiab] OR ehealth[tiab] OR mhealth[tiab] OR mobile health[tiab] OR remote consult*[tiab] OR videoconsult*[tiab] OR teleconsult*[tiab] OR telecare[tiab] OR e-consult*[tiab] OR virtual consult*[tiab] OR virtual visit*[tiab] OR virtual care[tiab] OR telestewardship[tiab])
AND
("Anti-Bacterial Agents"[Mesh] OR "Drug Therapy"[Mesh:NoExp] OR antibiotic*[tiab] OR anti-bacterial*[tiab] OR anti-microbial*[tiab] OR antibacterial*[tiab] OR antimicrobial*[tiab])
AND
("Drug Prescriptions"[Mesh] OR "Medication Therapy Management"[Mesh] OR stewardship[tiab] OR prescrib*[tiab] OR overprescrib*[tiab] OR over-prescrib*[tiab] OR de-prescrib*[tiab] OR deprescrib*[tiab] OR optimi*[tiab] OR optimiz*[tiab] OR appropriat*[tiab] OR inappropriat*[tiab] OR excess*[tiab] OR reduce*[tiab] OR decreas*[tiab] OR limit*[tiab] OR minimi*[tiab] OR utiliz*[tiab] OR utilis*[tiab] OR use[tiab])

Limiters - Published Date: 20100101-20240701

Embase

Search 1:
Title, abstract or author-specified keywords: ('telemedicine'/exp OR 'telehealth' OR 'ehealth' OR 'mhealth' OR 'mobile health' OR 'remote consult' OR 'videoconsult' OR 'teleconsult' OR 'telecare' OR 'e-consult' OR 'virtual consult' OR 'virtual visit' OR 'virtual care' OR 'telestewardship')
Search 2:
Title, abstract or author-specified keywords: (telemedicine OR telehealth OR ehealth OR mhealth OR 'mobile health' OR 'remote consult*' OR videoconsult* OR teleconsult* OR telecare OR 'e-consult*' OR 'virtual consult*' OR 'virtual visit*' OR 'virtual care' OR 'telestewardship')
Search 3:
Title, abstract or author-specified keywords: ('anti-bacterial agent'/exp OR 'antibiotic agent'/exp OR antibiotic* OR 'anti bacterial*' OR 'anti microbial*' OR antibacterial* OR antimicrobial*)
Search 4:
Title, abstract or author-specified keywords: (stewardship OR prescrib* OR overprescrib* OR 'over prescrib*' OR de-prescrib* OR deprescrib* OR optimi* OR optimiz* OR appropriat* OR inappropriat* OR excess* OR reduce* OR decreas* OR limit* OR minimi* OR utiliz* OR utilis* OR use)
Search 5:
Title, abstract or author-specified keywords: ('drug therapy'/exp OR 'medication therapy management'/exp OR 'drug prescription'/exp)
Search 6:
Title, abstract or author-specified keywords: (stewardship OR prescrib* OR overprescrib* OR 'over prescrib*' OR de-prescrib* OR deprescrib* OR optimi* OR optimiz* OR appropriat* OR inappropriat* OR excess* OR reduce* OR decreas* OR limit* OR minimi* OR utiliz* OR utilis* OR use)

Limiters - Published Date: 20100101-20240701

CINAHL

(MH "Telemedicine+" OR "telemedicine" OR "telehealth" OR "ehealth" OR "mhealth" OR "mobile health" OR "remote consult*" OR "videoconsult*" OR "teleconsult*" OR "telecare" OR "e-consult*" OR "virtual consult*" OR "virtual visit*" OR "virtual care" OR "telestewardship")
AND
(MH "Antiinfective Agents+" OR "antibiotic*" OR "anti-bacterial*" OR "anti-microbial*" OR "antibacterial*" OR "antimicrobial*")
AND
(MH "Medication Therapy Management" OR "Drug Prescriptions" OR "stewardship" OR "prescrib*" OR "overprescrib*" OR "over prescrib*" OR "de-prescrib*" OR "deprescrib*" OR "optimi*" OR "optimiz*" OR "appropriat*" OR "inappropriat*" OR "excess*" OR " reduce*" OR "decreas*" OR "limit*" OR "minimi*" OR "utiliz*" OR "utilis*" OR "use")

Limiters - Published Date: 20100101-20240701

Web of Science

TS=("Telemedicine" OR "telehealth" OR "ehealth" OR "mhealth" OR "mobile health" OR "remote consult*" OR "videoconsult*" OR "teleconsult*" OR "telecare" OR "e-consult*" OR "virtual consult*" OR "virtual visit*" OR "virtual care" OR "telestewardship" OR "tele-stewardship" OR "remote stewardship")
AND TS=("antibiotic*" OR "anti-bacterial*" OR "anti-microbial*" OR "antibacterial*" OR "antimicrobial*" OR "antimicrobial stewardship" OR "antibiotic stewardship" OR "AMS" OR "ASP")
AND TS=("stewardship" OR "prescrib*" OR "overprescrib*" OR "over-prescrib*" OR "de-prescrib*" OR "deprescrib*" OR "optimi*" OR "appropriat*" OR "inappropriat*" OR "excess*" OR "reduce*" OR "decreas*" OR "limit*" OR "minimi*" OR "utiliz*" OR "utilis*" OR "use" OR "oversight" OR "monitoring" OR "intervention*" OR "program*" OR "management")

Limiters - Published Date: 20100101-20240701

PsycINFO

CONCEPT 1: TELEHEALTH Search in:

1. Title: ("Telemedicine" OR "telehealth" OR "remote consult*" OR "teleconsult*" OR "virtual care" OR "telestewardship" OR "tele-stewardship")
2. Abstract: ("Telemedicine" OR "telehealth" OR "remote consult*" OR "teleconsult*" OR "virtual care" OR "telestewardship" OR "tele-stewardship")
3. Keywords: ("Telemedicine" OR "telehealth" OR "remote healthcare" OR "virtual care")
4. Index Terms: ("Telemedicine" OR "Telehealth" OR "Remote Consultation")

Operator: AND

CONCEPT 2: ANTIMICROBIAL Search in:

1. Title: (antibiotic* OR antimicrobial* OR "antimicrobial stewardship" OR "antibiotic stewardship" OR "ASP")
2. Abstract: (antibiotic* OR antimicrobial* OR "antimicrobial stewardship" OR "antibiotic stewardship" OR "ASP")
3. Keywords: ("antimicrobial stewardship" OR "antibiotic stewardship" OR "ASP")

4. MeSH: ("Anti-Bacterial Agents" OR "Antimicrobial Stewardship")

Operator: AND

CONCEPT 3: STEWARDSHIP/MANAGEMENT Search in:

1. Title: (steward* OR prescrib* OR "drug utili?ation" OR "medication management")
2. Abstract: (steward* OR prescrib* OR "drug utili?ation" OR "medication management")
3. Keywords: ("stewardship" OR "prescribing" OR "medication management")
4. Index Terms: ("Medication Management" OR "Drug Utilization")

Limiters:

- Publication Date: 2010-2024
- Language: English
- Source Type: Academic Journals

Google Scholar

“telemedicine” OR “telehealth” OR “ehealth” OR “mhealth” OR “mobile health” OR “remote consult” OR “video consult” OR “teleconsult” OR “telecare” OR “e-consult” OR “virtual consul” OR “virtual visit” OR “virtual care” OR "telestewardship"
AND
“antibiotic” “anti-bacterial” “anti-microbial” “anti-bacterial” “anti-microbial” “stewardship”
AND
(stewardship OR prescrib* OR overprescrib* OR “over prescrib*” OR de-prescrib* OR deprescrib* OR optimi* OR optimiz* OR appropriat* OR inappropriat* OR excess* OR reduce* OR decreas* OR limit* OR minimi* OR utiliz* OR utilis* OR use)

Limiters - Published Date: 20100101-20240701

Cochrane Central Register

1. [mh "Telemedicine"]
2. telemedicine OR telehealth OR ehealth OR mhealth OR "mobile health" OR "remote consult*" OR videoconsult* OR teleconsult* OR telecare OR "e-consult*" OR "virtual consult*" OR "virtual visit*" OR "virtual care" OR "telestewardship":ti,ab
3. #1 OR #2
4. [mh "Anti-Bacterial Agents"]
5. antibiotic* OR "anti-bacterial*" OR “anti-microbial*” OR antibacterial* OR antimicrobial*:ti,ab
6. #4 OR #5
7. [mh "Drug Therapy"] OR [mh "Medication Therapy Management"]
8. stewardship OR prescrib* OR overprescrib* OR “over prescrib*” OR de-prescrib* OR deprescrib* OR optimi* OR optimiz* OR appropriat* OR inappropriat* OR excess* OR reduce* OR decreas* OR limit* OR minimi* OR utiliz* OR utilis* OR use:ti,ab
9. #7 OR #8
10. #3 AND #6 AND #9

WHO Global Index Medicus

(telemedicine OR telehealth OR mhealth OR "mobile health" OR telestewardship)
AND
(antibiotic* OR anti-microbial*)
AND
(stewardship OR prescrib* OR optimi* OR appropriat*)

Date range: 2010-2024
