# Supplementary material for: Telemedicine interventions for improving antibiotic stewardship and prescribing: A systematic review
Source: PLoS One. 2025 Apr 3;20(4):e0320840. doi: 10.1371/journal.pone.0320840 (PMC11967954; doi:10.1371/journal.pone.0320840)
Supplement: S4 Appendix — (DOCX) [file pone.0320840.s004.docx]

**RTC, Cochrane risk-of-bias tool for randomized trials (RoB 2)**

# **1.** Education vs Clinician Feedback on Antibiotic Prescriptions for Acute Respiratory Infections in Telemedicine: a Randomized Controlled Trial [64].

**Domain 1: Risk of bias arising from the randomization process**

| **Signalling questions** | **Comments** | **Response options** |
| --- | --- | --- |
| **1.1 Was the allocation sequence random?** | The study randomized clinicians using a randomization sorting function performed by a programming engineer who was not involved in the study. Based on this, the allocation se The study investigators were blinded to the randomization process, indicating that the allocation sequence was concealed until enrollment and assignment. quence was generated randomly. | Y |
| **1.2 Was the allocation sequence concealed until participants were enrolled and assigned to interventions?** |  | Y |
| **1.3 Did baseline differences between intervention groups suggest a problem with the randomization process?** | Although the baseline antibiotic prescription rates differed between groups (e.g., 18.4% vs. 15.0% for URI, 46.8% vs. 64.0% for bronchitis), clinician and patient demographic characteristics were similar. This suggests that any differences likely arose by chance rather than from a flaw in the randomization process. | N |
| **Risk-of-bias judgement** |  |  |
| Optional: What is the predicted direction of bias arising from the randomization process? |  | NA |

Domain 2: Risk of bias due to deviations from the intended interventions (*effect of assignment to intervention*)

| **Signalling questions** | **Comments** | **Response options** |
| --- | --- | --- |
| **2.1. Were participants aware of their assigned intervention during the trial?** | Clinicians were aware of their assignment since those in the intervention arm received additional dashboard feedback, whereas those in the control arm received education only.  The personnel delivering the interventions (e.g., the Associate Medical Director providing the presentation and the online education system) were not blinded, as the intervention components differed clearly between arms. | Y |
| **2.2. Were carers and people delivering the interventions aware of participants' assigned intervention during the trial?** |  | Y |
| **2.3. If Y/PY/NI to 2.1 or 2.2: Were there deviations from the intended intervention that arose because of the trial context?** | There is no indication that the awareness of group assignment led to deviations from the planned interventions; both groups received the core educational content as intended. | N |
| **2.4 If Y/PY to 2.3: Were these deviations likely to have affected the outcome?** |  | NA |
| **2.5. If Y/PY/NI to 2.4: Were these deviations from intended intervention balanced between groups?** |  | NA |
| **2.6 Was an appropriate analysis used to estimate the effect of assignment to intervention?** | The study used an intention-to-treat analysis, which is appropriate for estimating the effect of assignment to the intervention. | Y |
| **2.7 If N/PN/NI to 2.6: Was there potential for a substantial impact (on the result) of the failure to analyse participants in the group to which they were randomized?** |  | NA |
| **Risk-of-bias judgement** |  | Low |
| Optional: What is the predicted direction of bias due to deviations from intended interventions? |  | NA |

Domain 2: Risk of bias due to deviations from the intended interventions (*effect of adhering to intervention*)

| **Signalling questions** | **Comments** | **Response options** |
| --- | --- | --- |
| **2.1. Were participants aware of their assigned intervention during the trial?** | Clinicians were aware of their group assignment because the intervention arm required active engagement with a personalized dashboard in addition to receiving the education.  As with the assignment phase, the delivery of both the educational content and the dashboard feedback was not blinded. | Y |
| **2.2. Were carers and people delivering the interventions aware of participants' assigned intervention during the trial?** |  | Y |
| **2.3. [If applicable:] If Y/PY/NI to 2.1 or 2.2: Were important non-protocol interventions balanced across intervention groups?** | There is no evidence that any non-protocol interventions were provided; both groups received the core educational components as planned. | Y |
| **2.4. [If applicable:] Were there failures in implementing the intervention that could have affected the outcome?** | Although there was some variability in the uptake of the educational components (with 4.3% of control clinicians and 22.7% of intervention clinicians not receiving any education), these variations were documented. There is no clear evidence that such deviations materially affected the outcome. | PN |
| **2.5. [If applicable:] Was there non-adherence to the assigned intervention regimen that could have affected participants’ outcomes?** | While a higher proportion of clinicians in the intervention group did not complete all components (notably, 22.7% did not receive any education compared to 4.3% in the control), the analysis was conducted on an intention-to-treat basis, which mitigates the potential impact of this non-adherence. | PN |
| **2.6. If N/PN/NI to 2.3, or Y/PY/NI to 2.4 or 2.5: Was an appropriate analysis used to estimate the effect of adhering to the intervention?** | The use of intention-to-treat analysis is appropriate in this context to account for deviations in adherence. | Y |
| **Risk-of-bias judgement** |  | Low |
| Optional: What is the predicted direction of bias due to deviations from intended interventions? |  | NA |

Domain 3: Missing outcome data

| **Signalling questions** | **Comments** | **Response options** |
| --- | --- | --- |
| **3.1 Were data for this outcome available for all, or nearly all, participants randomized?** | All clinicians were retained throughout the study period, and 99.8% of the total visits (55,364 out of 55,498) met the inclusion criteria, indicating that outcome data were available for nearly all participants. | Y |
| **3.2 If N/PN/NI to 3.1: Is there evidence that the result was not biased by missing outcome data?** |  | NA |
| **3.3 If N/PN to 3.2: Could missingness in the outcome depend on its true value?** |  | NA |
| **3.4 If Y/PY/NI to 3.3: Is it likely that missingness in the outcome depended on its true value?** |  | NA |
| **Risk-of-bias judgement** |  | Low |
| Optional: What is the predicted direction of bias due to missing outcome data? |  | NA |

Domain 4: Risk of bias in measurement of the outcome

| **Signalling questions** | **Comments** | **Response options** |
| --- | --- | --- |
| **4.1 Was the method of measuring the outcome inappropriate?** | The outcome—antibiotic prescription rates—was measured using data extracted from a proprietary electronic health record, which is an objective and standardized method. | N |
| **4.2 Could measurement or ascertainment of the outcome have differed between intervention groups?** | Data were collected uniformly from the electronic health record across all groups, making differential measurement unlikely. | N |
| **4.3 If N/PN/NI to 4.1 and 4.2: Were outcome assessors aware of the intervention received by study participants?** | Outcome data were de-identified and extracted automatically, reducing the risk that outcome assessors’ awareness of intervention allocation could influence the results. | N |
| **4.4 If Y/PY/NI to 4.3: Could assessment of the outcome have been influenced by knowledge of intervention received?** |  | NA |
| **4.5 If Y/PY/NI to 4.4:** **Is it likely that assessment of the outcome was influenced by knowledge of intervention received?** |  | NA |
| **Risk-of-bias judgement** |  | Low |
| Optional: What is the predicted direction of bias in measurement of the outcome? |  | NA |

Domain 5: Risk of bias in selection of the reported result

| **Signalling questions** | **Comments** | **Response options** |
| --- | --- | --- |
| **5.1 Were the data that produced this result analysed in accordance with a pre-specified analysis plan that was finalized before unblinded outcome data were available for analysis?** | The study’s statistical analysis methods are described in detail—including the use of mixed effects models, backwards selection of covariates, and an intention-to-treat approach—which suggests that analyses were conducted according to a pre-specified plan, even though explicit mention of protocol finalization is not provided. | PY |
| **Is the numerical result being assessed likely to have been selected, on the basis of the results, from...** |  |  |
| **5.2. ... multiple eligible outcome measurements (e.g. scales, definitions, time points) within the outcome domain?** | The primary outcomes (antibiotic prescription rates for URI, bronchitis, sinusitis, and pharyngitis) were clearly defined and reported consistently, with no indication that multiple alternative measurements were available or selectively chosen. | N |
| **5.3 ... multiple eligible analyses of the data?** | The analysis was conducted using appropriate statistical methods without evidence of multiple competing analyses being selectively reported. | N |
| **Risk-of-bias judgement** |  | Low |
| Optional: What is the predicted direction of bias due to selection of the reported result? |  | NA |

Overall risk of bias

| **Risk-of-bias judgement** |  | Low |
| --- | --- | --- |
| Optional: What is the overall predicted direction of bias for this outcome? |  | NA |

# 2. Remote Stewardship for Medically Underserved Nurseries: A SteppedWedge, Cluster Randomized Study [65].

**Domain 1: Risk of bias arising from the randomization process**

| **Signalling questions** | **Comments** | **Response options** |
| --- | --- | --- |
| **1.1 Was the allocation sequence random?** | The study employed a stepped‐wedge cluster randomized design in which the eight level 1 nurseries were assigned to receive the remote antimicrobial stewardship program (ASP) in a “randomly generated order” during the step‐in period. This indicates that the allocation sequence was generated by chance.  While the study states that the order of receiving the intervention was randomly generated, it does not provide explicit details regarding the concealment of this sequence prior to enrollment. However, given the cluster design and that each nursery served as its own control over time, there is no indication of compromised allocation; thus, any potential concerns are minimal. | Y |
| **1.2 Was the allocation sequence concealed until participants were enrolled and assigned to interventions?** |  | NI |
| **1.3 Did baseline differences between intervention groups suggest a problem with the randomization process?** | The study reports no statistically significant differences in demographics or clinical features between infants born in the preintervention and postintervention periods. This suggests that the randomization (i.e., the order in which nurseries crossed over) did not result in problematic baseline imbalances. | N |
| **Risk-of-bias judgement** |  | Low |
| Optional: What is the predicted direction of bias arising from the randomization process? |  | NA |

Domain 2: Risk of bias due to deviations from the intended interventions (*effect of assignment to intervention*)

| **Signalling questions** | **Comments** | **Response options** |
| --- | --- | --- |
| **2.1. Were participants aware of their assigned intervention during the trial?** | In this stepped‐wedge design, the nurseries (and by extension, their providers) were aware of when the remote ASP was implemented at their site. Thus, participants were not blinded to the intervention status.  The intervention (remote ASP) involved active provider-to-provider consultations, education sessions, and audit/feedback that were delivered openly at each nursery once the intervention was implemented. Therefore, the personnel delivering care were aware of the intervention. | Y |
| **2.2. Were carers and people delivering the interventions aware of participants' assigned intervention during the trial?** |  | Y |
| **2.3. If Y/PY/NI to 2.1 or 2.2: Were there deviations from the intended intervention that arose because of the trial context?** | There is no indication that awareness of the intervention led to systematic deviations from the intended delivery. Although the study acknowledges a “step-in” period and the absence of a wash-out period, these features are inherent to the stepped‐wedge design rather than deviations caused by lack of blinding. | N |
| **2.4 If Y/PY to 2.3: Were these deviations likely to have affected the outcome?** |  | NA |
| **2.5. If Y/PY/NI to 2.4: Were these deviations from intended intervention balanced between groups?** |  | NA |
| **2.6 Was an appropriate analysis used to estimate the effect of assignment to intervention?** | The study used an intention-to-treat approach by classifying infants as preintervention or postintervention based on whether the ASP was active at the time of birth, and employed multivariate mixed-effects logistic regression (or comparable models) appropriately adjusting for clustering by center. | Y |
| **2.7 If N/PN/NI to 2.6: Was there potential for a substantial impact (on the result) of the failure to analyse participants in the group to which they were randomized?** |  | NA |
| **Risk-of-bias judgement** |  | Low |
| Optional: What is the predicted direction of bias due to deviations from intended interventions? |  | NA |

Domain 2: Risk of bias due to deviations from the intended interventions (*effect of adhering to intervention*)

| **Signalling questions** | **Comments** | **Response options** |
| --- | --- | --- |
| **2.1. Were participants aware of their assigned intervention during the trial?** | As in Domain 2a, nursery staff and providers were aware of the intervention status because the remote ASP was actively delivered once Yes; providers, nurses, and other clinical staff were directly involved in receiving and delivering the components of the remote ASP, so they were aware of the intervention. implemented. | Y |
| **2.2. Were carers and people delivering the interventions aware of participants' assigned intervention during the trial?** |  | Y |
| **2.3. [If applicable:] If Y/PY/NI to 2.1 or 2.2: Were important non-protocol interventions balanced across intervention groups?** | There is no evidence from the study that non-protocol interventions (outside of the remote ASP components) were introduced or that any extraneous clinical practices were implemented unevenly between the preintervention and postintervention periods. | Y |
| **2.4. [If applicable:] Were there failures in implementing the intervention that could have affected the outcome?** | The study acknowledges a “step-in” period and the absence of a wash-out period, which might have led to a modest underestimation of the intervention’s efficacy during the initial learning phase. However, the rapid uptake of provider-to-provider consultations and the consistent reduction in antibiotic use across centers suggest that any implementation issues were minimal. | PN |
| **2.5. [If applicable:] Was there non-adherence to the assigned intervention regimen that could have affected participants’ outcomes?** | The study does not report systematic non-adherence to the intervention. All nurseries eventually received the remote ASP, and there is no indication that deviations in adherence materially affected the outcomes. | N |
| **2.6. If N/PN/NI to 2.3, or Y/PY/NI to 2.4 or 2.5: Was an appropriate analysis used to estimate the effect of adhering to the intervention?** | The analytical approach, which included multivariate mixed-effects models adjusting for the cluster design, appropriately accounted for the manner in which the intervention was implemented across centers. | Y |
| **Risk-of-bias judgement** |  | Low |
| Optional: What is the predicted direction of bias due to deviations from intended interventions? |  | NA |

Domain 3: Missing outcome data

| **Signalling questions** | **Comments** | **Response options** |
| --- | --- | --- |
| **3.1 Were data for this outcome available for all, or nearly all, participants randomized?** | The study reports outcome data for all 9,277 infants (4,586 preintervention and 4,691 postintervention) with no mention of losses to follow-up. This indicates that nearly complete data were available. | Y |
| **3.2 If N/PN/NI to 3.1: Is there evidence that the result was not biased by missing outcome data?** |  | NA |
| **3.3 If N/PN to 3.2: Could missingness in the outcome depend on its true value?** |  | NA |
| **3.4 If Y/PY/NI to 3.3: Is it likely that missingness in the outcome depended on its true value?** |  | NA |
| **Risk-of-bias judgement** |  | Low |
| Optional: What is the predicted direction of bias due to missing outcome data? |  | NA |

Domain 4: Risk of bias in measurement of the outcome

| **Signalling questions** | **Comments** | **Response options** |
| --- | --- | --- |
| **4.1 Was the method of measuring the outcome inappropriate?** | The primary outcomes—proportion of infants exposed to antibiotics and days of therapy (DOT) per 1000 patient-days—were measured using standardized, prospectively collected data abstraction sheets. These methods are appropriate and objective. | N |
| **4.2 Could measurement or ascertainment of the outcome have differed between intervention groups?** | Data collection was conducted in a similar fashion across all nurseries using a one-page abstraction form, and there is no indication that measurement differed between the preintervention and postintervention periods. | N |
| **4.3 If N/PN/NI to 4.1 and 4.2: Were outcome assessors aware of the intervention received by study participants?** | Although the study does not explicitly state the blinding status of outcome assessors, the use of objective data elements (e.g., antibiotic exposure, DOT) and standardized forms reduces the potential for measurement bias. | N |
| **4.4 If Y/PY/NI to 4.3: Could assessment of the outcome have been influenced by knowledge of intervention received?** |  | NA |
| **4.5 If Y/PY/NI to 4.4: Is it likely that assessment of the outcome was influenced by knowledge of intervention received?** |  | NA |
| **Risk-of-bias judgement** |  | Low |
| Optional: What is the predicted direction of bias in measurement of the outcome? |  | NA |

Domain 5: Risk of bias in selection of the reported result

| **Signalling questions** | **Comments** | **Response options** |
| --- | --- | --- |
| **5.1 Were the data that produced this result analysed in accordance with a pre-specified analysis plan that was finalized before unblinded outcome data were available for analysis?** | The study’s methods section details the statistical analysis plan—including the use of mixed-effects models, adjustment for clustering, and Bonferroni correction for the two primary outcomes. Although the report does not explicitly state that this plan was finalized before outcome data were available, the detailed description supports that a pre-specified plan was likely in place. | PY |
| **Is the numerical result being assessed likely to have been selected, on the basis of the results, from...** |  |  |
| **5.2. ... multiple eligible outcome measurements (e.g. scales, definitions, time points) within the outcome domain?** | The primary outcomes were clearly defined (antibiotic exposure and DOT per 1000 patient-days) with no evidence of multiple alternative measurements being available or selectively chosen. | N |
| **5.3 ... multiple eligible analyses of the data?** | The study employed a single, clearly described set of statistical analyses for the primary outcomes, and there is no indication of multiple competing analyses. | N |
| **Risk-of-bias judgement** |  | Low |
| Optional: What is the predicted direction of bias due to selection of the reported result? |  | NA |

Overall risk of bias

| **Risk-of-bias judgement** |  | Low |
| --- | --- | --- |
| Optional: What is the overall predicted direction of bias for this outcome? |  | NA |

**Non- RTC, ROBINS-1**

# 1: Mobile health application to assist doctors in antibiotic prescription – an approach for antibiotic stewardship [53].

|  | **Signalling questions** | **Description** | **Response options** |
| --- | --- | --- | --- |
| **Bias due to confounding** | | | |
|  | 1.1 Is there potential for confounding of the effect of intervention in this study?  **If N/PN to 1.1:** the study can be considered to be at low risk of bias due to confounding and no further signalling questions need be considered | The study was an observational, analytical, longitudinal design comparing 12 months before and after the implementation of a mobile health application for antibiotic prescription. The authors used segmented regression to account for time trends, and no major baseline differences or confounding factors were reported between the pre- and post-intervention periods. Based on this, the study likely minimized or accounted for confounding, suggesting that potential confounding was not a major concern | PN |
|  | **If Y/PY to 1.1**: determine whether there is a need to assess time-varying confounding: |  |  |
|  | 1.2. Was the analysis based on splitting participants’ follow up time according to intervention received?  **If N/PN**, answer questions relating to baseline confounding (1.4 to 1.6)  **If Y/PY**, go to question 1.3. |  | NA |
|  | 1.3. Were intervention discontinuations or switches likely to be related to factors that are prognostic for the outcome?  **If N/PN**, answer questions relating to baseline confounding (1.4 to 1.6)  **If Y/PY**, answer questions relating to both baseline and time-varying confounding (1.7 and 1.8) |  | NA |

|  | **Questions relating to baseline confounding only** | | |
| --- | --- | --- | --- |
|  | 1.4. Did the authors use an appropriate analysis method that controlled for all the important confounding domains? |  | NA |
|  | 1.5. **If Y/PY to 1.4**: Were confounding domains that were controlled for measured validly and reliably by the variables available in this study? |  | NA |
|  | 1.6. Did the authors control for any post-intervention variables that could have been affected by the intervention? |  | NA |
|  | **Questions relating to baseline and time-varying confounding** | |  |
|  | 1.7. Did the authors use an appropriate analysis method that controlled for all the important confounding domains and for time-varying confounding? |  | NA |
|  | 1.8. **If Y/PY to 1.7**: Were confounding domains that were controlled for measured validly and reliably by the variables available in this study? |  | NA |
|  | **Risk of bias judgement** |  | Low |
|  | Optional: What is the predicted direction of bias due to confounding? |  | Unpredictable |

| **Bias in selection of participants into the study** | | | |
| --- | --- | --- | --- |
|  | 2.1. Was selection of participants into the study (or into the analysis) based on participant characteristics observed after the start of intervention?  **If N/PN to 2.1:** go to 2.4 | This observational study included all patients before and after the implementation of the mobile health application. There was no indication that any patients were selected or excluded based on characteristics that emerged after the intervention began. Based on this, there is no evidence that participant selection depended on post-intervention variables | N |
|  | 2.2. **If Y/PY to 2.1**: Were the post-intervention variables that influenced selection likely to be associated with intervention?  2.3 **If Y/PY to 2.2**: Were the post-intervention variables that influenced selection likely to be influenced by the outcome or a cause of the outcome? |  | NA  NA |
|  | 2.4. Do start of follow-up and start of intervention coincide for most participants? | The before-and-after design used hospital-wide data, capturing all relevant participants at the transition point (pre-intervention period versus post-intervention period). Therefore, the start of follow-up effectively coincided with the introduction of the mobile application for most participants | Y |
|  | 2.5. **If Y/PY to 2.2 and 2.3, or N/PN to 2.4**: Were adjustment techniques used that are likely to correct for the presence of selection biases? |  | NA |
|  | **Risk of bias judgement** |  | Low |
|  | Optional: What is the predicted direction of bias due to selection of participants into the study? |  | Unpredictable |

| **Bias in classification of interventions** | | | |
| --- | --- | --- | --- |
|  | 3.1 Were intervention groups clearly defined? | The study compared outcomes in two distinct time periods: before the mobile application was available (pre-intervention) and after it was implemented (post-intervention). These two groups were clearly defined and corresponded to the presence or absence of the intervention (the app). | Y |
|  | 3.2 Was the information used to define intervention groups recorded at the start of the intervention? | The classification into pre- and post-implementation periods was inherently linked to the time the mobile app was introduced (December 2014). The data for each participant’s exposure status—being ‘pre-app’ or ‘post-app’—was determined at the start of the new intervention period. | Y |
|  | 3.3 Could classification of intervention status have been affected by knowledge of the outcome or risk of the outcome? | Since the intervention status was defined solely by the time period (before vs after app implementation), investigators or participants could not alter the classification based on any emerging outcomes. This temporal design precludes the possibility that knowledge of outcomes could affect how the intervention was classified. | N |
|  | **Risk of bias judgement** |  | Low |
|  | Optional: What is the predicted direction of bias due to classification of interventions? |  | Unpredictable |

| **Bias due to deviations from intended interventions** | | | |
| --- | --- | --- | --- |
|  | **If your aim for this study is to assess the effect of assignment to intervention, answer questions 4.1 and 4.2** | |  |
|  | 4.1. Were there deviations from the intended intervention beyond what would be expected in usual practice? | In this study, the mobile health application was introduced hospital-wide, with all physicians encouraged to use it for antibiotic prescribing. There is no indication of any substantial deviations from the intended use beyond normal practice variations (e.g., some physicians may have used the app more frequently than others, but there were no reported major deviations). | N |
|  | 4.2. **If Y/PY to 4.1**: Were these deviations from intended intervention unbalanced between groups *and* likely to have affected the outcome? |  | NA |
|  | **If your aim for this study is to assess the effect of starting and adhering to intervention, answer questions 4.3 to 4.6** | |  |
|  | 4.3. Were important co-interventions balanced across intervention groups? |  | Y / PY / PN / N / NI |
|  | 4.4. Was the intervention implemented successfully for most participants? |  | Y / PY / PN / N / NI |
|  | 4.5. Did study participants adhere to the assigned intervention regimen? |  | Y / PY / PN / N / NI |
|  | 4.6. **If N/PN to 4.3, 4.4 or 4.5**: Was an appropriate analysis used to estimate the effect of starting and adhering to the intervention? |  | NA / Y / PY / PN / N / NI |
|  | **Risk of bias judgement** |  | Low |
|  | Optional: What is the predicted direction of bias due to deviations from the intended interventions? |  | Unpredictable |

| **Bias due to missing data** | | | |
| --- | --- | --- | --- |
|  | 5.1 Were outcome data available for all, or nearly all, participants? | The study collected comprehensive hospital-wide data on antibiotic use (DDD per 1000 patient-days) and related costs for each month, before and after the application’s implementation. This aggregated information covers nearly all patients within the institution, indicating minimal, if any, missing outcome data. | Y |
|  | 5.2 Were participants excluded due to missing data on intervention status? | All data were categorized simply by time period (pre-intervention versus post-intervention). No exclusions were reported due to unavailable intervention status. | N |
|  | 5.3 Were participants excluded due to missing data on other variables needed for the analysis? | The paper does not mention excluding participants for missing covariates or other key data points. The analysis focused primarily on institutional consumption and resistance rates, which were fully available. | N |
|  | 5.4 **If PN/N to 5.1, or Y/PY to 5.2 or 5.3**: Are the proportion of participants and reasons for missing data similar across interventions? |  | NA |
|  | 5.5 **If PN/N to 5.1, or Y/PY to 5.2 or 5.3**: Is there evidence that results were robust to the presence of missing data? |  | NA |
|  | **Risk of bias judgement** |  | Low |
|  | Optional: What is the predicted direction of bias due to missing data? |  | Unpredictable |

| **Bias in measurement of outcomes** | | | |
| --- | --- | --- | --- |
|  | 6.1 Could the outcome measure have been influenced by knowledge of the intervention received? | The primary outcomes—antibiotic consumption (DDD per 1000 patient-days), cost data (in USD), and microbial susceptibility rates—were collected via routine hospital records and laboratory databases. These objective measures are unlikely to be influenced by awareness of the mobile application, as they involve standardized, institution-wide data tracking. | N |
|  | 6.2 Were outcome assessors aware of the intervention received by study participants? | Although outcome assessors might have known about the implementation of the mobile app (as it was a hospital-wide change), the measured outcomes (e.g., aggregated antibiotic usage, costs) were derived from automated pharmacy and financial records. Thus, any awareness by the data-collecting personnel was unlikely to influence how the outcomes were recorded. | Y  Yes, but the outcome measures were objective (e.g., pharmacy records, lab data), thus reducing the risk that knowledge of the intervention would bias the measurement. |
|  | 6.3 Were the methods of outcome assessment comparable across intervention groups? | The methods for measuring antibiotic consumption (pharmacy records), costs (financial statements), and bacterial susceptibility (microbiology lab) did not change between the pre- and post-app periods. Therefore, the outcome assessment was comparable across both time frames. | Y |
|  | 6.4 Were any systematic errors in measurement of the outcome related to intervention received? | Given that all outcomes were extracted from standard electronic systems, there is no indication of systematic measurement errors specific to the intervention. The same procedures for recording antibiotic use and costs were consistently applied before and after the app’s introduction. | N |
|  | **Risk of bias judgement** |  | Low |
|  | Optional: What is the predicted direction of bias due to measurement of outcomes? |  | Unpredictable |

| **Bias in selection of the reported result** | | | |
| --- | --- | --- | --- |
|  | Is the reported effect estimate likely to be selected, on the basis of the results, from... |  |  |
|  | 7.1. ... multiple outcome *measurements* within the outcome domain? | The authors focused on well-defined outcomes—antimicrobial consumption, cost, and susceptibility—and reported them all. There is no indication that multiple alternative measures of these same outcomes were collected but not reported. | N |
|  | 7.2 ... multiple *analyses* of the intervention-outcome relationship? | The study used standard segmented regression analyses for its before-and-after design. There is no evidence of multiple unpublished or unreported analyses that would introduce selective reporting. | N |
|  | 7.3 ... different *subgroups*? | No subgroup analyses were reported or hinted at in the manuscript, and there is no suggestion that any subgroups were selectively omitted based on the results. | N |
|  | **Risk of bias judgement** |  | Low |
|  | Optional: What is the predicted direction of bias due to selection of the reported result? |  | Unpredictable |

| **Overall bias** | | | |
| --- | --- | --- | --- |
|  | **Risk of bias judgement** |  | Low |
|  | Optional: What is the overall predicted direction of bias for this outcome? |  | Unpredictable |

# 2: Impact of a Telehealth-based Antimicrobial Stewardship Program in a Community Hospital Health System [54].

|  | **Signalling questions** | **Description** | **Response options** |
| --- | --- | --- | --- |
| **Bias due to confounding** | | | |
|  | 1.1 Is there potential for confounding of the effect of intervention in this study?  **If N/PN to 1.1:** the study can be considered to be at low risk of bias due to confounding and no further signalling questions need be considered | The study used a before-and-after (pre-post) design, collecting antimicrobial usage data (DOT/1000 patient-days) over 12 months prior to the TeleASP intervention (baseline) and 6 months after (intervention). Segmented linear regression analysis was employed to account for time trends. The article does not identify additional major confounders or changes in hospital practice (aside from the new ASP), and no alternative secular interventions appear to have coincided with TeleASP that could systematically bias the results. Therefore, there is likely minimal risk of confounding. | PN |
|  | **If Y/PY to 1.1**: determine whether there is a need to assess time-varying confounding: |  |  |
|  | 1.2. Was the analysis based on splitting participants’ follow up time according to intervention received?  **If N/PN**, answer questions relating to baseline confounding (1.4 to 1.6)  **If Y/PY**, go to question 1.3. | The study analyzed antimicrobial utilization data for 12 months pre-implementation (March 2017-February 2018) and 6 months post-implementation (March 2018-August 2018). The analysis was not based on splitting individual patient follow-up time but rather on aggregate hospital-level data comparing distinct time periods. | NA |
|  | 1.3. Were intervention discontinuations or switches likely to be related to factors that are prognostic for the outcome?  **If N/PN**, answer questions relating to baseline confounding (1.4 to 1.6)  **If Y/PY**, answer questions relating to both baseline and time-varying confounding (1.7 and 1.8) |  | NA |

|  | **Questions relating to baseline confounding only** | | |
| --- | --- | --- | --- |
|  | 1.4. Did the authors use an appropriate analysis method that controlled for all the important confounding domains? |  | NA |
|  | 1.5. **If Y/PY to 1.4**: Were confounding domains that were controlled for measured validly and reliably by the variables available in this study? |  | NA |
|  | 1.6. Did the authors control for any post-intervention variables that could have been affected by the intervention? |  | NA |
|  | **Questions relating to baseline and time-varying confounding** | |  |
|  | 1.7. Did the authors use an appropriate analysis method that controlled for all the important confounding domains and for time-varying confounding? |  | NA |
|  | 1.8. **If Y/PY to 1.7**: Were confounding domains that were controlled for measured validly and reliably by the variables available in this study? |  | NA |
|  | **Risk of bias judgement** |  | Low |
|  | Optional: What is the predicted direction of bias due to confounding? |  | Unpredictable |

| **Bias in selection of participants into the study** | | | |
| --- | --- | --- | --- |
|  | 2.1. Was selection of participants into the study (or into the analysis) based on participant characteristics observed after the start of intervention?  **If N/PN to 2.1:** go to 2.4 | All patients hospitalized at the two HVHS community hospitals were included in aggregated antimicrobial usage data. There is no mention of excluding patients post hoc or selecting them based on variables that emerged after TeleASP began. Hence, participant selection did not depend on any post-intervention characteristics. | N |
|  | 2.2. **If Y/PY to 2.1**: Were the post-intervention variables that influenced selection likely to be associated with intervention?  2.3 **If Y/PY to 2.2**: Were the post-intervention variables that influenced selection likely to be influenced by the outcome or a cause of the outcome? |  | NA  NA |
|  | 2.4. Do start of follow-up and start of intervention coincide for most participants? | The baseline period was defined as March 2017–February 2018, and the intervention period started immediately afterward (March 2018–August 2018). Thus, follow-up corresponds clearly to ‘pre’ vs. ‘post’ TeleASP phases for all patients. | Y |
|  | 2.5. **If Y/PY to 2.2 and 2.3, or N/PN to 2.4**: Were adjustment techniques used that are likely to correct for the presence of selection biases? |  | NA |
|  | **Risk of bias judgement** |  | Low |
|  | Optional: What is the predicted direction of bias due to selection of participants into the study? |  | Favours experimental / Favours comparator / Towards null /Away from null / Unpredictable |

| **Bias in classification of interventions** | | | |
| --- | --- | --- | --- |
|  | 3.1 Were intervention groups clearly defined? | Two distinct periods were examined: baseline (no formal ASP) vs. post-implementation (TeleASP). These two time-based groups were clearly defined by the presence or absence of the ASP. | Y |
|  | 3.2 Was the information used to define intervention groups recorded at the start of the intervention? | Patients in the post-intervention group were identified starting exactly when TeleASP was launched in February/March 2018. Classification (‘baseline’ vs. ‘intervention’) was predetermined by the TeleASP start date. | Y |
|  | 3.3 Could classification of intervention status have been affected by knowledge of the outcome or risk of the outcome? | Because the classification is simply by time period, it was not influenced by individuals’ clinical outcomes. Knowledge of outcomes would not alter whether a patient fell into the pre- or post-TeleASP group. | N |
|  | **Risk of bias judgement** |  | Low |
|  | Optional: What is the predicted direction of bias due to classification of interventions? |  | Favours experimental / Favours comparator / Towards null /Away from null / Unpredictable |

| **Bias due to deviations from intended interventions** | | | |
| --- | --- | --- | --- |
|  | **If your aim for this study is to assess the effect of assignment to intervention, answer questions 4.1 and 4.2** | |  |
|  | 4.1. Were there deviations from the intended intervention beyond what would be expected in usual practice? | The TeleASP was implemented as planned: HVHS pharmacists reviewed patients on Tier 1 and Tier 2 antimicrobials, then discussed cases via scheduled calls with the AHN ID physicians. There is no indication of major deviations from this model that might systematically affect outcomes. Minor variations (e.g., duration of calls, availability of local pharmacists) are within normal practice | N |
|  | 4.2. **If Y/PY to 4.1**: Were these deviations from intended intervention unbalanced between groups *and* likely to have affected the outcome? |  | NA |
|  | **If your aim for this study is to assess the effect of starting and adhering to intervention, answer questions 4.3 to 4.6** | |  |
|  | 4.3. Were important co-interventions balanced across intervention groups? |  | Y / PY / PN / N / NI |
|  | 4.4. Was the intervention implemented successfully for most participants? |  | Y / PY / PN / N / NI |
|  | 4.5. Did study participants adhere to the assigned intervention regimen? |  | Y / PY / PN / N / NI |
|  | 4.6. **If N/PN to 4.3, 4.4 or 4.5**: Was an appropriate analysis used to estimate the effect of starting and adhering to the intervention? |  | NA / Y / PY / PN / N / NI |
|  | **Risk of bias judgement** |  | Low |
|  | Optional: What is the predicted direction of bias due to deviations from the intended interventions? |  | Unpredictable |

| **Bias due to missing data** | | | |
| --- | --- | --- | --- |
|  | 5.1 Were outcome data available for all, or nearly all, participants? | The study reports aggregated DOT (days of therapy) per 1000 patient-days for the entire hospital population in both baseline and intervention phases. These data come from standard pharmacy records, covering essentially all inpatients (i.e., minimal missing data). | Y |
|  | 5.2 Were participants excluded due to missing data on intervention status? | No participants appear to be excluded based on unclear intervention status. The classification is fully time-based. | N |
|  | 5.3 Were participants excluded due to missing data on other variables needed for the analysis? | No mention of exclusions for missing covariates; the main variables—DOT and patient-days—were available for the entire hospital population from standard tracking systems. | N |
|  | 5.4 **If PN/N to 5.1, or Y/PY to 5.2 or 5.3**: Are the proportion of participants and reasons for missing data similar across interventions? |  | NA |
|  | 5.5 **If PN/N to 5.1, or Y/PY to 5.2 or 5.3**: Is there evidence that results were robust to the presence of missing data? |  | NA |
|  | **Risk of bias judgement** |  | Low |
|  | Optional: What is the predicted direction of bias due to missing data? |  | Unpredictable |

| **Bias in measurement of outcomes** | | | |
| --- | --- | --- | --- |
|  | 6.1 Could the outcome measure have been influenced by knowledge of the intervention received? | Primary outcomes were antimicrobial utilization (DOT per 1000 patient-days) and ID consult rates. These are objective measures, typically extracted from administrative and pharmacy records, and unlikely to be influenced by awareness of the TeleASP. | N |
|  | 6.2 Were outcome assessors aware of the intervention received by study participants? | Although the ASP team knew TeleASP was in place, the data used for analysis (e.g., pharmacy records, ID consult logs) are systematically recorded. Awareness by those abstracting the data should not meaningfully affect these objective records. | Y  (But objective measures minimize bias.) |
|  | 6.3 Were the methods of outcome assessment comparable across intervention groups? | Data collection methods (e.g., pharmacy systems, ID consult logs) were consistent in both the pre- and post-intervention periods. Hence, outcome assessment was comparable. | Y |
|  | 6.4 Were any systematic errors in measurement of the outcome related to intervention received? | There is no indication of systematic differences in how DOT or consult rates were measured before vs. after TeleASP. Measurement bias due to the intervention itself is unlikely | N |
|  | **Risk of bias judgement** |  | Low |
|  | Optional: What is the predicted direction of bias due to measurement of outcomes? |  | Favours experimental / Favours comparator / Towards null /Away from null / Unpredictable |

| **Bias in selection of the reported result** | | | |
| --- | --- | --- | --- |
|  | Is the reported effect estimate likely to be selected, on the basis of the results, from... |  |  |
|  | 7.1. ... multiple outcome *measurements* within the outcome domain? | The authors explicitly focused on days of therapy (DOT) per 1000 patient-days, ID consult rates, and acceptance of ASP recommendations. There is no indication of unpublished alternative measures of the same outcomes that were withheld based on the findings. | N |
|  | 7.2 ... multiple *analyses* of the intervention-outcome relationship? | The main analysis was segmented linear regression plus a straightforward pre-post comparison for DOT/1000 patient-days and consult rates. The authors did not report multiple, selective analyses for the same outcomes. | N |
|  | 7.3 ... different *subgroups*? | No subgroup analyses (e.g., by patient population) appear to have been selectively presented or omitted. The intervention was applied hospital-wide, and results are reported at the hospital system level. | N |
|  | **Risk of bias judgement** |  | Low |
|  | Optional: What is the predicted direction of bias due to selection of the reported result? |  | Unpredictable |

| **Overall bias** | | | |
| --- | --- | --- | --- |
|  | **Risk of bias judgement** |  | Low |
|  | Optional: What is the overall predicted direction of bias for this outcome? |  | Unpredictable |

# 3: Implementation of an Infectious Diseases Telehealth Consultation and Antibiotic Stewardship Program for 16 Small Community Hospitals [55].

|  | **Signalling questions** | **Description** | **Response options** |
| --- | --- | --- | --- |
| **Bias due to confounding** | | | |
|  | 1.1 Is there potential for confounding of the effect of intervention in this study?  **If N/PN to 1.1:** the study can be considered to be at low risk of bias due to confounding and no further signalling questions need be considered | The study implemented an infectious diseases telehealth (IDt) consultation and antibiotic stewardship program in a stepwise manner across 16 small community hospitals. It compared outcomes (such as telehealth consultation workload, antibiotic usage rates, and stewardship interventions) before and after program implementation. The study design does not report any concurrent changes or additional interventions that would systematically influence the outcomes aside from the IDt program. Based on these details, the study appears to have addressed potential confounding adequately. | PN |
|  | **If Y/PY to 1.1**: determine whether there is a need to assess time-varying confounding: |  |  |
|  | 1.2. Was the analysis based on splitting participants’ follow up time according to intervention received?  **If N/PN**, answer questions relating to baseline confounding (1.4 to 1.6)  **If Y/PY**, go to question 1.3. |  | NA |
|  | 1.3. Were intervention discontinuations or switches likely to be related to factors that are prognostic for the outcome?  **If N/PN**, answer questions relating to baseline confounding (1.4 to 1.6)  **If Y/PY**, answer questions relating to both baseline and time-varying confounding (1.7 and 1.8) |  | NA |

|  | **Questions relating to baseline confounding only** | | |
| --- | --- | --- | --- |
|  | 1.4. Did the authors use an appropriate analysis method that controlled for all the important confounding domains? |  | NA |
|  | 1.5. **If Y/PY to 1.4**: Were confounding domains that were controlled for measured validly and reliably by the variables available in this study? |  | NA |
|  | 1.6. Did the authors control for any post-intervention variables that could have been affected by the intervention? |  | NA |
|  | **Questions relating to baseline and time-varying confounding** | |  |
|  | 1.7. Did the authors use an appropriate analysis method that controlled for all the important confounding domains and for time-varying confounding? |  | NA |
|  | 1.8. **If Y/PY to 1.7**: Were confounding domains that were controlled for measured validly and reliably by the variables available in this study? |  | NA |
|  | **Risk of bias judgement** |  | Low |
|  | Optional: What is the predicted direction of bias due to confounding? |  | Unpredictable |

| **Bias in selection of participants into the study** | | | |
| --- | --- | --- | --- |
|  | 2.1. Was selection of participants into the study (or into the analysis) based on participant characteristics observed after the start of intervention?  **If N/PN to 2.1:** go to 2.4 | The study utilized routinely collected electronic data (from the EMR and REDCap) for all 16 community hospitals over defined baseline and intervention periods. There is no indication that participants (i.e., hospitalized patients) were selected or excluded based on characteristics that emerged after the intervention began. Based on this, the study does not appear to have introduced selection bias through post-intervention characteristics. | N |
|  | 2.2. **If Y/PY to 2.1**: Were the post-intervention variables that influenced selection likely to be associated with intervention?  2.3 **If Y/PY to 2.2**: Were the post-intervention variables that influenced selection likely to be influenced by the outcome or a cause of the outcome? |  | NA  NA |
|  | 2.4. Do start of follow-up and start of intervention coincide for most participants? | The study clearly defined a baseline period (prior to implementation) and an intervention period (after implementation), ensuring that the start of follow-up corresponded to the start of the IDt program for all hospitals. Based on this, the timing of follow-up was aligned with the intervention’s initiation. | Y |
|  | 2.5. **If Y/PY to 2.2 and 2.3, or N/PN to 2.4**: Were adjustment techniques used that are likely to correct for the presence of selection biases? | Given that participant selection was not influenced by post-intervention characteristics and the timing was clearly defined, there was no need for additional adjustment techniques to correct for selection bias. | NA |
|  | **Risk of bias judgement** |  | Low |
|  | Optional: What is the predicted direction of bias due to selection of participants into the study? |  | Unpredictable |

| **Bias in classification of interventions** | | | |
| --- | --- | --- | --- |
|  | 3.1 Were intervention groups clearly defined? | The study compared two distinct periods: the pre-implementation (baseline) phase and the post-implementation (intervention) phase during which the IDt consultation and stewardship program was active. These groups are defined purely by time and the presence or absence of the intervention, ensuring clear classification. | Y |
|  | 3.2 Was the information used to define intervention groups recorded at the start of the intervention? | Intervention status was determined by the fixed start date of the IDt program. The study clearly designated baseline data (before implementation) and intervention data (after implementation), ensuring that group assignment was established at the start of the intervention. | Y |
|  | 3.3 Could classification of intervention status have been affected by knowledge of the outcome or risk of the outcome? | Because the intervention groups were defined solely by the calendar period (pre- vs. post-IDt program), there was no opportunity for outcome data or anticipated risks to influence group classification. Based on this, misclassification driven by outcome awareness is unlikely. | N |
|  | **Risk of bias judgement** |  | Low |
|  | Optional: What is the predicted direction of bias due to classification of interventions? |  | Unpredictable |

| **Bias due to deviations from intended interventions** | | | |
| --- | --- | --- | --- |
|  | **If your aim for this study is to assess the effect of assignment to intervention, answer questions 4.1 and 4.2** | |  |
|  | 4.1. Were there deviations from the intended intervention beyond what would be expected in usual practice? | The study describes a structured implementation of the IDt consultation and antibiotic stewardship program—including standardized telehealth consultations, defined roles for IDt physicians, pharmacists, and other staff, and routine data collection—across all participating hospitals. There is no indication that the program deviated from the intended protocol beyond typical practice variations. Based on this, the study did not exhibit deviations that would introduce additional bias. | N |
|  | 4.2. **If Y/PY to 4.1**: Were these deviations from intended intervention unbalanced between groups *and* likely to have affected the outcome? |  | NA |
|  | **If your aim for this study is to assess the effect of starting and adhering to intervention, answer questions 4.3 to 4.6** | |  |
|  | 4.3. Were important co-interventions balanced across intervention groups? |  | Y / PY / PN / N / NI |
|  | 4.4. Was the intervention implemented successfully for most participants? |  | Y / PY / PN / N / NI |
|  | 4.5. Did study participants adhere to the assigned intervention regimen? |  | Y / PY / PN / N / NI |
|  | 4.6. **If N/PN to 4.3, 4.4 or 4.5**: Was an appropriate analysis used to estimate the effect of starting and adhering to the intervention? |  | NA / Y / PY / PN / N / NI |
|  | **Risk of bias judgement** |  | Low |
|  | Optional: What is the predicted direction of bias due to deviations from the intended interventions? |  | Unpredictable |

| **Bias due to missing data** | | | |
| --- | --- | --- | --- |
|  | 5.1 Were outcome data available for all, or nearly all, participants? | The study collected outcome data from the electronic medical records, REDCap logs, and NHSN AU reports across all 16 community hospitals over the defined periods. There is no mention of significant missing data or exclusions due to unavailable outcomes. Based on this, outcome data appear to be comprehensive. | Y |
|  | 5.2 Were participants excluded due to missing data on intervention status? | Participants were classified into baseline or intervention groups based on a predetermined timeline, and there is no indication that any patients were excluded because of missing information regarding intervention status. | N |
|  | 5.3 Were participants excluded due to missing data on other variables needed for the analysis? | The study does not report any exclusions related to missing data on key variables (e.g., telehealth consultation details, antibiotic usage data). The available data were derived from integrated electronic systems covering all sites. | PN |
|  | 5.4 **If PN/N to 5.1, or Y/PY to 5.2 or 5.3**: Are the proportion of participants and reasons for missing data similar across interventions? |  | NA |
|  | 5.5 **If PN/N to 5.1, or Y/PY to 5.2 or 5.3**: Is there evidence that results were robust to the presence of missing data? |  | NA |
|  | **Risk of bias judgement** |  | Low |
|  | Optional: What is the predicted direction of bias due to missing data? |  | Unpredictable |

| **Bias in measurement of outcomes** | | | |
| --- | --- | --- | --- |
|  | 6.1 Could the outcome measure have been influenced by knowledge of the intervention received? | The outcomes (such as the number and types of telehealth consultations, antibiotic usage measured in DOT per 1000 days present, and satisfaction survey responses) were obtained from objective data sources including EMR records, REDCap logs, and standardized NHSN AU reports. These objective measures are unlikely to be influenced by knowledge of whether the IDt program was in place. | N |
|  | 6.2 Were outcome assessors aware of the intervention received by study participants? | Although those recording the outcomes (e.g., IDt service staff and data abstractors) were aware that the IDt program was active during the intervention period, the outcomes were collected through automated or standardized electronic systems. This minimizes the risk that awareness would affect the measurement | Y |
|  | 6.3 Were the methods of outcome assessment comparable across intervention groups? | The methods used to capture outcomes (EMR note templates, REDCap data entries, and antibiotic usage dashboards) remained consistent across both the pre-intervention (baseline) and post-intervention periods. This ensured that outcome assessment was comparable between groups. | Y |
|  | 6.4 Were any systematic errors in measurement of the outcome related to intervention received? | There is no indication that the introduction of the IDt program itself altered the methods or accuracy of outcome measurement. The same standardized systems were used in both periods, making systematic measurement errors unlikely. | N |
|  | **Risk of bias judgement** |  | Low |
|  | Optional: What is the predicted direction of bias due to measurement of outcomes? |  | Unpredictable |

| **Bias in selection of the reported result** | | | |
| --- | --- | --- | --- |
|  | Is the reported effect estimate likely to be selected, on the basis of the results, from... |  |  |
|  | 7.1. ... multiple outcome *measurements* within the outcome domain? | The study reports predefined outcomes such as telehealth consultation types and volumes, antibiotic usage rates, and satisfaction survey responses. There is no evidence that multiple alternative measures within these domains were available and selectively reported. Based on the description, selective reporting is unlikely. | N |
|  | 7.2 ... multiple *analyses* of the intervention-outcome relationship? | The analyses performed (e.g., descriptive statistics, t-tests, segmented regression for antibiotic usage comparisons) are clearly specified in the methods section. There is no indication of multiple, competing analyses from which the reported estimates were selectively chosen. | N |
|  | 7.3 ... different *subgroups*? | The study does not indicate that subgroup analyses were selectively reported or omitted. The outcomes are presented at the level of the overall IDt program across the 16 community hospitals, minimizing the risk of selective subgroup reporting. | N |
|  | **Risk of bias judgement** |  | Low |
|  | Optional: What is the predicted direction of bias due to selection of the reported result? |  | Unpredictable |

| **Overall bias** | | | |
| --- | --- | --- | --- |
|  | **Risk of bias judgement** |  | Low |
|  | Optional: What is the overall predicted direction of bias for this outcome? |  | Unpredictable |

# 4: Implementing a tele-expertise system to optimise the antibiotic use and stewardship: The case of the Montpellier University Hospital (France) [56].

|  | **Signalling questions** | **Description** | **Response options** |
| --- | --- | --- | --- |
| **Bias due to confounding** | | | |
|  | 1.1 Is there potential for confounding of the effect of intervention in this study?  **If N/PN to 1.1:** the study can be considered to be at low risk of bias due to confounding and no further signalling questions need be considered | The study is an observational prospective design evaluating the implementation of a tele-expertise system at Montpellier University Hospital. It collected data on tele-expertise actions, IDS (Infectious Disease Specialist) recommendations, and prescriber adherence using electronic medical records (EMR) and system notifications over a 12‐month period. The intervention was uniformly implemented without concurrent changes that could affect the outcomes. Based on this, the study adequately addressed potential confounding | PN |
|  | **If Y/PY to 1.1**: determine whether there is a need to assess time-varying confounding: |  |  |
|  | 1.2. Was the analysis based on splitting participants’ follow up time according to intervention received?  **If N/PN**, answer questions relating to baseline confounding (1.4 to 1.6)  **If Y/PY**, go to question 1.3. |  | NA |
|  | 1.3. Were intervention discontinuations or switches likely to be related to factors that are prognostic for the outcome?  **If N/PN**, answer questions relating to baseline confounding (1.4 to 1.6)  **If Y/PY**, answer questions relating to both baseline and time-varying confounding (1.7 and 1.8) |  | NA |

|  | **Questions relating to baseline confounding only** | | |
| --- | --- | --- | --- |
|  | 1.4. Did the authors use an appropriate analysis method that controlled for all the important confounding domains? |  | NA |
|  | 1.5. **If Y/PY to 1.4**: Were confounding domains that were controlled for measured validly and reliably by the variables available in this study? |  | NA |
|  | 1.6. Did the authors control for any post-intervention variables that could have been affected by the intervention? |  | NA |
|  | **Questions relating to baseline and time-varying confounding** | |  |
|  | 1.7. Did the authors use an appropriate analysis method that controlled for all the important confounding domains and for time-varying confounding? |  | NA |
|  | 1.8. **If Y/PY to 1.7**: Were confounding domains that were controlled for measured validly and reliably by the variables available in this study? |  | NA |
|  | **Risk of bias judgement** |  | Low |
|  | Optional: What is the predicted direction of bias due to confounding? |  | Unpredictable |

| **Bias in selection of participants into the study** | | | |
| --- | --- | --- | --- |
|  | 2.1. Was selection of participants into the study (or into the analysis) based on participant characteristics observed after the start of intervention?  **If N/PN to 2.1:** go to 2.4 | The study included 870 inpatients for whom tele-expertise actions were recorded via the EMR and IDS notifications. There is no indication that patients were selected or excluded based on characteristics that emerged after the tele-expertise system was initiated. Based on this, participant selection was not influenced by post-intervention factors | N |
|  | 2.2. **If Y/PY to 2.1**: Were the post-intervention variables that influenced selection likely to be associated with intervention?  2.3 **If Y/PY to 2.2**: Were the post-intervention variables that influenced selection likely to be influenced by the outcome or a cause of the outcome? |  | NA  NA |
|  | 2.4. Do start of follow-up and start of intervention coincide for most participants? | The study defined a specific data collection period starting more than six months after system implementation. All patients in the study were classified into the intervention phase based on a clear start date for the tele-expertise system. Based on this, the start of follow-up coincided with the start of the intervention. | Y |
|  | 2.5. **If Y/PY to 2.2 and 2.3, or N/PN to 2.4**: Were adjustment techniques used that are likely to correct for the presence of selection biases? |  | NA |
|  | **Risk of bias judgement** |  | Low |
|  | Optional: What is the predicted direction of bias due to selection of participants into the study? |  | Unpredictable |

| **Bias in classification of interventions** | | | |
| --- | --- | --- | --- |
|  | 3.1 Were intervention groups clearly defined? | The tele-expertise system was protocol-driven, involving structured EMR documentation and IDS counselling. Intervention groups (e.g., prescribers using the system) were unambiguously defined | Y |
|  | 3.2 Was the information used to define intervention groups recorded at the start of the intervention? | Intervention status was determined by the implementation date of the tele-expertise system. Data collection started after the system was fully operational, ensuring that classification into the intervention group was made at the onset. Based on this, intervention group assignment is appropriately recorded. | Y |
|  | 3.3 Could classification of intervention status have been affected by knowledge of the outcome or risk of the outcome? | Since the classification is solely based on the system’s implementation date and the corresponding EMR entries, there is no indication that outcome data or anticipated risks influenced group classification. Based on this, the study addressed classification without bias. | N |
|  | **Risk of bias judgement** |  | Low |
|  | Optional: What is the predicted direction of bias due to classification of interventions? |  | Unpredictable |

| **Bias due to deviations from intended interventions** | | | |
| --- | --- | --- | --- |
|  | **If your aim for this study is to assess the effect of assignment to intervention, answer questions 4.1 and 4.2** | |  |
|  | 4.1. Were there deviations from the intended intervention beyond what would be expected in usual practice? | The tele-expertise system was implemented as planned at Montpellier University Hospital, with IDS advice provided via a dedicated form integrated into the EMR. The process followed a standardized protocol (including predefined items for classification and traceability), and there is no indication of deviations beyond typical variations in clinical practice. Based on this, no concerning deviations occurred | N |
|  | 4.2. **If Y/PY to 4.1**: Were these deviations from intended intervention unbalanced between groups *and* likely to have affected the outcome? | Since there were no notable deviations from the intended intervention, this question is not applicable. | NA |
|  | **If your aim for this study is to assess the effect of starting and adhering to intervention, answer questions 4.3 to 4.6** | |  |
|  | 4.3. Were important co-interventions balanced across intervention groups? |  | Y / PY / PN / N / NI |
|  | 4.4. Was the intervention implemented successfully for most participants? |  | Y / PY / PN / N / NI |
|  | 4.5. Did study participants adhere to the assigned intervention regimen? |  | Y / PY / PN / N / NI |
|  | 4.6. **If N/PN to 4.3, 4.4 or 4.5**: Was an appropriate analysis used to estimate the effect of starting and adhering to the intervention? |  | NA / Y / PY / PN / N / NI |
|  | **Risk of bias judgement** |  | Low |
|  | Optional: What is the predicted direction of bias due to deviations from the intended interventions? |  | Unpredictable |

| **Bias due to missing data** | | | |
| --- | --- | --- | --- |
|  | 5.1 Were outcome data available for all, or nearly all, participants? | The study used data from the EMR and from IDS notifications to capture 1,386 tele-expertise actions for 870 inpatients, and adherence was evaluated for 927 clinical situations. There is no mention of significant missing data or exclusions due to incomplete records. Based on this, outcome data were nearly complete | Y |
|  | 5.2 Were participants excluded due to missing data on intervention status? | The classification of intervention status was based on a predefined system implementation date, and no patients were excluded due to missing intervention information. Based on this, no exclusion occurred on this basis | N |
|  | 5.3 Were participants excluded due to missing data on other variables needed for the analysis? | The study does not report any exclusions related to missing clinical or process data required for the evaluation of tele-expertise actions and prescriber adherence. Based on this, missing data did not result in exclusions. | N |
|  | 5.4 **If PN/N to 5.1, or Y/PY to 5.2 or 5.3**: Are the proportion of participants and reasons for missing data similar across interventions? |  | NA / Y / PY / PN / N / NI |
|  | 5.5 **If PN/N to 5.1, or Y/PY to 5.2 or 5.3**: Is there evidence that results were robust to the presence of missing data? |  | NA / Y / PY / PN / N / NI |
|  | **Risk of bias judgement** |  | Low |
|  | Optional: What is the predicted direction of bias due to missing data? |  | Unpredictable |

| **Bias in measurement of outcomes** | | | |
| --- | --- | --- | --- |
|  | 6.1 Could the outcome measure have been influenced by knowledge of the intervention received? | The outcomes—such as the number of tele-expertise actions, prescriber adherence rates to IDS recommendations, and satisfaction levels—were measured using objective data from the EMR and through a structured prospective survey. These measures are not likely to be influenced by awareness of the tele-expertise system. Based on this, outcome measurement is robust. | N |
|  | 6.2 Were outcome assessors aware of the intervention received by study participants? | Outcome data were derived from automated entries in the EMR and from a predefined notification system. Although IDS staff and prescribers were aware of the tele-expertise system, the use of standardized electronic records minimizes the potential for bias due to assessors’ awareness. Based on this, any such awareness is unlikely to have affected the outcomes. | Y |
|  | 6.3 Were the methods of outcome assessment comparable across intervention groups? | Since the study involves a single intervention group with all outcomes measured using the same EMR and tele-expertise data collection tools, the methods of outcome assessment were consistent throughout. Based on this, the assessment methods are comparable. | Y |
|  | 6.4 Were any systematic errors in measurement of the outcome related to intervention received? | There is no indication in the study that the implementation of the tele-expertise system led to systematic errors in outcome measurement. The use of a predefined electronic notification form and consistent data extraction procedures supports accurate measurement. Based on this, systematic errors are unlikely | N |
|  | **Risk of bias judgement** |  | Low |
|  | Optional: What is the predicted direction of bias due to measurement of outcomes? |  | Unpredictable |

| **Bias in selection of the reported result** | | | |
| --- | --- | --- | --- |
|  | Is the reported effect estimate likely to be selected, on the basis of the results, from... |  |  |
|  | 7.1. ... multiple outcome *measurements* within the outcome domain? | The study reports predetermined outcomes, including the number of tele-expertise actions, prescriber adherence rates, and satisfaction metrics. There is no evidence that alternative outcome measures were available but selectively reported. Based on this, the risk of selective outcome reporting is minimal. | N |
|  | 7.2 ... multiple *analyses* of the intervention-outcome relationship? | The analyses conducted (e.g., calculation of adherence percentages and descriptive statistics for tele-expertise actions) were specified a priori. There is no indication of multiple, competing analyses from which one effect estimate was selectively chosen. Based on this, the reported estimates are appropriate | N |
|  | 7.3 ... different *subgroups*? | The study does not appear to report subgroup analyses in a selective manner; outcomes were reported for the overall sample of tele-expertise actions and for defined clinical situations. Based on this, selective subgroup reporting is unlikely | N |
|  | **Risk of bias judgement** |  | Low |
|  | Optional: What is the predicted direction of bias due to selection of the reported result? |  | Unpredictable |

| **Overall bias** | | | |
| --- | --- | --- | --- |
|  | **Risk of bias judgement** |  | Low |
|  | Optional: What is the overall predicted direction of bias for this outcome? |  | Unpredictable |

# 5: Pilot implementation of a telemedicine care bundle: Antimicrobial stewardship, patient satisfaction, clinician satisfaction, and usability in patients with sinusitis [57].

|  | **Signalling questions** | **Description** | **Response options** |
| --- | --- | --- | --- |
| **Bias due to confounding** | | | |
|  | 1.1 Is there potential for confounding of the effect of intervention in this study?  **If N/PN to 1.1:** the study can be considered to be at low risk of bias due to confounding and no further signalling questions need be considered | The study is a pilot implementation using a pre–post observational design within NYU Virtual Urgent Care (VUC). It compares outcomes during a 15‐month pre-implementation period (January 1, 2021 to February 28, 2022) with a 14‐month post-implementation period (March 1, 2022 to May 31, 2023) after introducing a sinusitis care bundle (comprising a clinical pathway, standardized orders, and patient education elements via the EHR). The study does not report any concurrent changes or additional interventions that would confound the relationship between the care bundle and outcomes such as antimicrobial prescribing, satisfaction, or usability. Based on this, the study addressed potential confounding adequately | PN |
|  | **If Y/PY to 1.1**: determine whether there is a need to assess time-varying confounding: |  |  |
|  | 1.2. Was the analysis based on splitting participants’ follow up time according to intervention received?  **If N/PN**, answer questions relating to baseline confounding (1.4 to 1.6)  **If Y/PY**, go to question 1.3. |  | NA |
|  | 1.3. Were intervention discontinuations or switches likely to be related to factors that are prognostic for the outcome?  **If N/PN**, answer questions relating to baseline confounding (1.4 to 1.6)  **If Y/PY**, answer questions relating to both baseline and time-varying confounding (1.7 and 1.8) |  | NA |

|  | **Questions relating to baseline confounding only** | | |
| --- | --- | --- | --- |
|  | 1.4. Did the authors use an appropriate analysis method that controlled for all the important confounding domains? |  | NA |
|  | 1.5. **If Y/PY to 1.4**: Were confounding domains that were controlled for measured validly and reliably by the variables available in this study? |  | NA |
|  | 1.6. Did the authors control for any post-intervention variables that could have been affected by the intervention? |  | NA |
|  | **Questions relating to baseline and time-varying confounding** | |  |
|  | 1.7. Did the authors use an appropriate analysis method that controlled for all the important confounding domains and for time-varying confounding? |  | NA |
|  | 1.8. **If Y/PY to 1.7**: Were confounding domains that were controlled for measured validly and reliably by the variables available in this study? |  | NA |
|  | **Risk of bias judgement** |  | Low |
|  | Optional: What is the predicted direction of bias due to confounding? |  | Unpredictable |

| **Bias in selection of participants into the study** | | | |
| --- | --- | --- | --- |
|  | 2.1. Was selection of participants into the study (or into the analysis) based on participant characteristics observed after the start of intervention?  **If N/PN to 2.1:** go to 2.4 | The study is based on data from all VUC visits with a sinusitis diagnosis, identified via ICD-10 codes in the integrated Epic system. Participants were included based on the diagnosis recorded during the visit, with no evidence that selection was based on characteristics emerging after the care bundle was implemented. Based on this, participant selection was not biased by post-intervention characteristics. | N |
|  | 2.2. **If Y/PY to 2.1**: Were the post-intervention variables that influenced selection likely to be associated with intervention?  2.3 **If Y/PY to 2.2**: Were the post-intervention variables that influenced selection likely to be influenced by the outcome or a cause of the outcome? |  | NA  NA |
|  | 2.4. Do start of follow-up and start of intervention coincide for most participants? | The study clearly defines its pre- and post-intervention periods by fixed calendar dates corresponding to the activation of the care bundle components (e.g., SCPW on March 1, 2022; SOP and SPESP shortly thereafter). This ensures that follow-up for each patient is correctly aligned with whether they were seen before or after the care bundle was implemented. Based on this, the start of follow-up coincides with the intervention’s start. | Y |
|  | 2.5. **If Y/PY to 2.2 and 2.3, or N/PN to 2.4**: Were adjustment techniques used that are likely to correct for the presence of selection biases? | Because participants were included based on a clearly defined time period and there is no evidence of exclusions due to post-intervention characteristics, no additional adjustment techniques were necessary to address selection bias. Based on this, the study adequately addressed selection issues. | NA |
|  | **Risk of bias judgement** |  | Low |
|  | Optional: What is the predicted direction of bias due to selection of participants into the study? |  | Unpredictable |

| **Bias in classification of interventions** | | | |
| --- | --- | --- | --- |
|  | 3.1 Were intervention groups clearly defined? | The study distinguishes between a pre-care bundle period and a post-care bundle period, with the care bundle consisting of the sinusitis clinical pathway, standardized orders, and patient education smart-phrase clearly implemented through the VUC platform. Based on this, the intervention groups are clearly defined. | Y |
|  | 3.2 Was the information used to define intervention groups recorded at the start of the intervention? | The classification into pre- and post-intervention groups was determined by the exact dates when the care bundle components were activated in the EHR (e.g., March 1, 2022 for the SCPW). This ensured that group assignment was established at the start of the intervention. Based on this, the study addressed the issue appropriately. | Y |
|  | 3.3 Could classification of intervention status have been affected by knowledge of the outcome or risk of the outcome? | The assignment of visits to either the pre- or post-intervention period was based solely on the visit date relative to the care bundle implementation. There is no evidence that outcome data or anticipated outcomes influenced this classification. Based on this, the study addressed potential misclassification bias. | N |
|  | **Risk of bias judgement** |  | Low |
|  | Optional: What is the predicted direction of bias due to classification of interventions? |  | Unpredictable |

| **Bias due to deviations from intended interventions** | | | |
| --- | --- | --- | --- |
|  | **If your aim for this study is to assess the effect of assignment to intervention, answer questions 4.1 and 4.2** | |  |
|  | 4.1. Were there deviations from the intended intervention beyond what would be expected in usual practice? | The study describes the implementation of a sinusitis care bundle with standardized orders, communication scripts, and integrated EHR tools (ExpressLane, SOP, SPESP) that were disseminated via email and faculty meetings. There is no indication that the intervention was not delivered as planned or that significant deviations occurred beyond normal clinical practice variations. Based on this, the study did not experience concerning deviations. | N |
|  | 4.2. **If Y/PY to 4.1**: Were these deviations from intended intervention unbalanced between groups *and* likely to have affected the outcome? |  | NA |
|  | **If your aim for this study is to assess the effect of starting and adhering to intervention, answer questions 4.3 to 4.6** | |  |
|  | 4.3. Were important co-interventions balanced across intervention groups? |  | Y / PY / PN / N / NI |
|  | 4.4. Was the intervention implemented successfully for most participants? |  | Y / PY / PN / N / NI |
|  | 4.5. Did study participants adhere to the assigned intervention regimen? |  | Y / PY / PN / N / NI |
|  | 4.6. **If N/PN to 4.3, 4.4 or 4.5**: Was an appropriate analysis used to estimate the effect of starting and adhering to the intervention? |  | NA / Y / PY / PN / N / NI |
|  | **Risk of bias judgement** |  | Low |
|  | Optional: What is the predicted direction of bias due to deviations from the intended interventions? |  | Unpredictable |

| **Bias due to missing data** | | | |
| --- | --- | --- | --- |
|  | 5.1 Were outcome data available for all, or nearly all, participants? | The study extracted data on antimicrobial prescribing, patient satisfaction (via Q-ReviewsSM), clinician satisfaction, and usability from the Epic Clarity database and survey systems for all VUC visits with a sinusitis diagnosis. Although the patient satisfaction survey response rate is approximately 10%, the overall dataset on key outcomes (e.g., prescribing rates, visit volumes) is complete. Based on this, outcome data were available for nearly all relevant encounters. | Y |
|  | 5.2 Were participants excluded due to missing data on intervention status? | Participants were assigned to the pre- or post-intervention groups solely based on the visit date recorded in the EHR, and no exclusions were made due to missing intervention status. Based on this, the study addressed this issue appropriately. | N |
|  | 5.3 Were participants excluded due to missing data on other variables needed for the analysis? | There is no report in the study of excluding patients because of missing information on antimicrobial prescribing, satisfaction, or usability metrics; rather, aggregate data were analyzed. Based on this, no exclusions due to missing data occurred | N |
|  | 5.4 **If PN/N to 5.1, or Y/PY to 5.2 or 5.3**: Are the proportion of participants and reasons for missing data similar across interventions? |  | NA / Y / PY / PN / N / NI |
|  | 5.5 **If PN/N to 5.1, or Y/PY to 5.2 or 5.3**: Is there evidence that results were robust to the presence of missing data? |  | NA / Y / PY / PN / N / NI |
|  | **Risk of bias judgement** |  | Low |
|  | Optional: What is the predicted direction of bias due to missing data? |  | Unpredictable |

| **Bias in measurement of outcomes** | | | |
| --- | --- | --- | --- |
|  | 6.1 Could the outcome measure have been influenced by knowledge of the intervention received? | The study’s primary outcomes—antimicrobial prescribing rates, patient satisfaction survey responses, clinician satisfaction, and usability scores—were obtained from objective data sources (Epic Clarity database, Q-ReviewsSM, and structured surveys). These measures are not easily influenced by the awareness of the care bundle intervention. Based on this, the outcome measurement is robust. | N |
|  | 6.2 Were outcome assessors aware of the intervention received by study participants? | Outcome data were extracted from automated electronic systems and standardized survey platforms. Although clinicians and patients were aware of the telemedicine care bundle during their visits, the data collection methods minimized assessor influence on the measured outcomes. Based on this, any awareness is unlikely to have introduced bias | Y |
|  | 6.3 Were the methods of outcome assessment comparable across intervention groups? | The same methods (e.g., queries via Oracle SQL Developer for prescribing rates, standardized survey platforms for satisfaction measures) were used consistently in both the pre- and post-intervention periods. Based on this, outcome assessment was comparable across groups | Y |
|  | 6.4 Were any systematic errors in measurement of the outcome related to intervention received? | There is no indication that the implementation of the care bundle altered the methods or accuracy of outcome measurement. Data were captured consistently using the same EHR and survey tools before and after implementation. Based on this, systematic measurement errors related to the intervention are unlikely | N |
|  | **Risk of bias judgement** |  | Low |
|  | Optional: What is the predicted direction of bias due to measurement of outcomes? |  | Unpredictable |

| **Bias in selection of the reported result** | | | |
| --- | --- | --- | --- |
|  | Is the reported effect estimate likely to be selected, on the basis of the results, from... |  |  |
|  | 7.1. ... multiple outcome *measurements* within the outcome domain? | The study pre-specified key outcomes (antimicrobial prescribing rates, patient satisfaction, clinician satisfaction, and usability) and reported these measures without indication that alternative outcome measurements were available but selectively chosen. Based on this, the risk of selective outcome reporting is minimal. | N |
|  | 7.2 ... multiple *analyses* of the intervention-outcome relationship? | Analyses (including Pearson’s chi-square tests and SPC chart evaluations) were performed as described in the methods section, with no evidence that multiple competing analyses were conducted and selectively reported. Based on this, selective analysis is unlikely. | N |
|  | 7.3 ... different *subgroups*? | The study reports overall outcomes for patients with a sinusitis diagnosis across the VUC system and does not indicate that subgroup analyses were selectively reported. Based on this, selective subgroup reporting is unlikely. | N |
|  | **Risk of bias judgement** |  | Low |
|  | Optional: What is the predicted direction of bias due to selection of the reported result? |  | Unpredictable |

| **Overall bias** | | | |
| --- | --- | --- | --- |
|  | **Risk of bias judgement** |  | Low |
|  | Optional: What is the overall predicted direction of bias for this outcome? |  | Favours experimental / Favours comparator / Towards null /Away from null / Unpredictable |

# 6: Multidrug stewardship and adherence to guidelines in >200,000 direct-to-consumer Telemedicine encounters [58].

|  | **Signalling questions** | **Description** | **Response options** |
| --- | --- | --- | --- |
| **Bias due to confounding** | | | |
|  | 1.1 Is there potential for confounding of the effect of intervention in this study?  **If N/PN to 1.1:** the study can be considered to be at low risk of bias due to confounding and no further signalling questions need be considered | The study is a retrospective observational analysis in which all consecutive adult patients with new respiratory symptoms were managed under a uniform intervention—namely, the implementation of multidrug stewardship protocols during telemedicine encounters. The protocols were applied consistently across all patients, and there is no indication that baseline differences or other factors confounded the relationship between the intervention and the outcomes. Based on this, the study adequately addresses the issue of confounding." | N |
|  | **If Y/PY to 1.1**: determine whether there is a need to assess time-varying confounding: |  |  |
|  | 1.2. Was the analysis based on splitting participants’ follow up time according to intervention received?  **If N/PN**, answer questions relating to baseline confounding (1.4 to 1.6)  **If Y/PY**, go to question 1.3. |  | NA |
|  | 1.3. Were intervention discontinuations or switches likely to be related to factors that are prognostic for the outcome?  **If N/PN**, answer questions relating to baseline confounding (1.4 to 1.6)  **If Y/PY**, answer questions relating to both baseline and time-varying confounding (1.7 and 1.8) |  | NA |

|  | **Questions relating to baseline confounding only** | | |
| --- | --- | --- | --- |
|  | 1.4. Did the authors use an appropriate analysis method that controlled for all the important confounding domains? |  | NA |
|  | 1.5. **If Y/PY to 1.4**: Were confounding domains that were controlled for measured validly and reliably by the variables available in this study? |  | NA |
|  | 1.6. Did the authors control for any post-intervention variables that could have been affected by the intervention? |  | NA |
|  | **Questions relating to baseline and time-varying confounding** | |  |
|  | 1.7. Did the authors use an appropriate analysis method that controlled for all the important confounding domains and for time-varying confounding? |  | NA |
|  | 1.8. **If Y/PY to 1.7**: Were confounding domains that were controlled for measured validly and reliably by the variables available in this study? |  | NA |
|  | **Risk of bias judgement** |  | Low |
|  | Optional: What is the predicted direction of bias due to confounding? |  | Unpredictable |

| **Bias in selection of participants into the study** | | | |
| --- | --- | --- | --- |
|  | 2.1. Was selection of participants into the study (or into the analysis) based on participant characteristics observed after the start of intervention?  **If N/PN to 2.1:** go to 2.4 | The study included all consecutive adult patients presenting with new respiratory symptoms within the past 14 days; selection was based solely on pre-intervention (baseline) clinical criteria rather than any characteristics observed after the intervention commenced. Based on this, the study addressed potential selection bias appropriately. | N |
|  | 2.2. **If Y/PY to 2.1**: Were the post-intervention variables that influenced selection likely to be associated with intervention?  2.3 **If Y/PY to 2.2**: Were the post-intervention variables that influenced selection likely to be influenced by the outcome or a cause of the outcome? |  | NA  NA |
|  | 2.4. Do start of follow-up and start of intervention coincide for most participants? | In this study, the start of follow-up is inherently linked to the telemedicine consultation, which is when the multidrug stewardship protocols are applied. Thus, the initiation of follow-up and the intervention coincide for all participants. Based on this, the study adequately addresses concerns regarding timing. | Y |
|  | 2.5. **If Y/PY to 2.2 and 2.3, or N/PN to 2.4**: Were adjustment techniques used that are likely to correct for the presence of selection biases? |  | NA |
|  | **Risk of bias judgement** |  | Low |
|  | Optional: What is the predicted direction of bias due to selection of participants into the study? |  | Unpredictable |

| **Bias in classification of interventions** | | | |
| --- | --- | --- | --- |
|  | 3.1 Were intervention groups clearly defined? | The study clearly defines the intervention as the application of multidrug stewardship protocols during telemedicine encounters. All patients received care under the same protocol, ensuring clear classification of the intervention. Based on this, the study adequately addresses this issue. | Y |
|  | 3.2 Was the information used to define intervention groups recorded at the start of the intervention? | Information to define the intervention was recorded concurrently with the telemedicine consultation. Details such as diagnostic codes and treatment plans were documented at the time the protocols were applied, ensuring that intervention status was determined at the start. Based on this, the study addresses this aspect appropriately. | Y |
|  | 3.3 Could classification of intervention status have been affected by knowledge of the outcome or risk of the outcome? | The classification of intervention status was based on pre-established institutional protocols implemented before any outcome was known. There is no indication that outcome knowledge influenced how the intervention was classified. Based on this, the study adequately addresses this potential source of bias. | N |
|  | **Risk of bias judgement** |  | Low |
|  | Optional: What is the predicted direction of bias due to classification of interventions? |  | Unpredictable |

| **Bias due to deviations from intended interventions** | | | |
| --- | --- | --- | --- |
|  | **If your aim for this study is to assess the effect of assignment to intervention, answer questions 4.1 and 4.2** | |  |
|  | 4.1. Were there deviations from the intended intervention beyond what would be expected in usual practice? |  | Y / PY / PN / N / NI |
|  | 4.2. **If Y/PY to 4.1**: Were these deviations from intended intervention unbalanced between groups *and* likely to have affected the outcome? |  | NA / Y / PY / PN / N / NI |
|  | **If your aim for this study is to assess the effect of starting and adhering to intervention, answer questions 4.3 to 4.6** | |  |
|  | 4.3. Were important co-interventions balanced across intervention groups? | The study implemented a standardized multidrug stewardship protocol across all telemedicine encounters, thereby ensuring that any co-interventions (e.g., symptomatic treatments) were applied uniformly. Based on this, the study adequately balances potential co-interventions. | Y |
|  | 4.4. Was the intervention implemented successfully for most participants? | High adherence to the protocol is evident in the study, with 85.65% of patients managed at home and 86.22% receiving a pharmacological treatment plan. Additionally, immediate individualized feedback to physicians supports successful implementation. Based on this, the intervention was implemented successfully for most participants. | Y |
|  | 4.5. Did study participants adhere to the assigned intervention regimen? | The study ensured adherence to the multidrug stewardship protocols by providing continuous feedback and monitoring quality indicators during telemedicine encounters. This systematic approach indicates that the intervention regimen was adhered to effectively by the healthcare providers managing the patients. Based on this, adherence was high. | Y |
|  | 4.6. **If N/PN to 4.3, 4.4 or 4.5**: Was an appropriate analysis used to estimate the effect of starting and adhering to the intervention? |  | NA |
|  | **Risk of bias judgement** |  | Low |
|  | Optional: What is the predicted direction of bias due to deviations from the intended interventions? |  | Unpredictable |

| **Bias due to missing data** | | | |
| --- | --- | --- | --- |
|  | 5.1 Were outcome data available for all, or nearly all, participants? | The study utilized comprehensive de-identified electronic medical records from over 221,000 telemedicine encounters, ensuring that outcome data (such as diagnostic codes and treatment plans) were available for nearly all participants. Based on this, the study adequately addresses concerns regarding missing data. | Y |
|  | 5.2 Were participants excluded due to missing data on intervention status? | There is no indication that any participants were excluded because of missing data on the intervention status; all consecutive patients meeting the inclusion criteria were included. Based on this, the study addresses this issue appropriately. | N |
|  | 5.3 Were participants excluded due to missing data on other variables needed for the analysis? | The study did not report any exclusions due to missing data on variables necessary for the analysis, indicating that the dataset was sufficiently complete. Based on this, missing data does not introduce bias. | N |
|  | 5.4 **If PN/N to 5.1, or Y/PY to 5.2 or 5.3**: Are the proportion of participants and reasons for missing data similar across interventions? |  | NA |
|  | 5.5 **If PN/N to 5.1, or Y/PY to 5.2 or 5.3**: Is there evidence that results were robust to the presence of missing data? |  | NA |
|  | **Risk of bias judgement** |  | Low |
|  | Optional: What is the predicted direction of bias due to missing data? |  | Unpredictable |

| **Bias in measurement of outcomes** | | | |
| --- | --- | --- | --- |
|  | 6.1 Could the outcome measure have been influenced by knowledge of the intervention received? | Outcome measures in the study—such as prescription patterns and diagnostic codes—were recorded objectively through the telemedicine system. There is no indication that these measures were influenced by knowledge of the intervention, as data collection was standardized. Based on this, the study adequately addresses potential measurement bias. | N |
|  | 6.2 Were outcome assessors aware of the intervention received by study participants? | The outcomes were derived from de-identified electronic records and not subject to subjective assessment. Outcome assessors did not have the opportunity to be influenced by knowledge of the intervention. Based on this, the study adequately addresses this issue. | N |
|  | 6.3 Were the methods of outcome assessment comparable across intervention groups? | A uniform method was used to assess outcomes for all telemedicine encounters, ensuring that diagnostic codes, treatment plans, and referral data were collected in the same manner across all participants. Based on this, the study adequately addresses comparability in outcome assessment. | Y |
|  | 6.4 Were any systematic errors in measurement of the outcome related to intervention received? | There is no evidence of systematic errors in the measurement of outcomes that could be related to the intervention. The standardized protocols used for data collection minimize the possibility of such errors. Based on this, the study adequately addresses this issue. | N |
|  | **Risk of bias judgement** |  | Low |
|  | Optional: What is the predicted direction of bias due to measurement of outcomes? |  | Unpredictable |

| **Bias in selection of the reported result** | | | |
| --- | --- | --- | --- |
|  | Is the reported effect estimate likely to be selected, on the basis of the results, from... |  |  |
|  | 7.1. ... multiple outcome *measurements* within the outcome domain? | The study pre-specified key outcomes, such as diagnostic categorization, prescription rates, and referral rates, and reported these outcomes comprehensively. There is no indication that the effect estimates were selectively chosen from multiple measurements. Based on this, the study addresses this issue appropriately. | N |
|  | 7.2 ... multiple *analyses* of the intervention-outcome relationship? | The analysis in the study is straightforward and descriptive, with no evidence of multiple competing analyses being selectively reported. Based on this, the study adequately addresses the risk of selective reporting. | N |
|  | 7.3 ... different *subgroups*? | Although the study reports outcomes for various diagnostic subgroups (e.g., confirmed COVID-19, suspected COVID-19, other diagnoses), the subgroup analyses were pre-specified and reported in full. There is no evidence of selective reporting based on subgroup results. Based on this, the study addresses this issue appropriately | N |
|  | **Risk of bias judgement** |  | Low |
|  | Optional: What is the predicted direction of bias due to selection of the reported result? |  | Unpredictable |

| **Overall bias** | | | |
| --- | --- | --- | --- |
|  | **Risk of bias judgement** |  | Low |
|  | Optional: What is the overall predicted direction of bias for this outcome? |  | Unpredictable |

# 7: Antibiotic prescribing in remote versus face-to-face consultations for acute respiratory infections in primary care in England: an observational study using target maximum likelihood estimation [59].

|  | **Signalling questions** | **Description** | **Response options** |
| --- | --- | --- | --- |
| **Bias due to confounding** | | | |
|  | 1.1 Is there potential for confounding of the effect of intervention in this study?  **If N/PN to 1.1:** the study can be considered to be at low risk of bias due to confounding and no further signalling questions need be considered | The study is an observational cohort analysis using routinely collected data from CPRD Aurum. Although any observational study has inherent potential for confounding, the authors have extensively adjusted for a wide range of confounders at the consultation, patient, clinician, and practice levels—including demographics, comorbidities, prior consultation patterns, indices of deprivation, and even regional COVID-19 prevalence. Based on this comprehensive adjustment, it appears that the key confounding domains were addressed. | PY |
|  | **If Y/PY to 1.1**: determine whether there is a need to assess time-varying confounding: |  |  |
|  | 1.2. Was the analysis based on splitting participants’ follow up time according to intervention received?  **If N/PN**, answer questions relating to baseline confounding (1.4 to 1.6)  **If Y/PY**, go to question 1.3. | The exposure—consultation mode—is determined at the time of the consultation, and there is no longitudinal splitting of follow-up time by intervention status. Based on this, the analysis addresses only baseline confounding. | N |
|  | 1.3. Were intervention discontinuations or switches likely to be related to factors that are prognostic for the outcome?  **If N/PN**, answer questions relating to baseline confounding (1.4 to 1.6)  **If Y/PY**, answer questions relating to both baseline and time-varying confounding (1.7 and 1.8) |  | NA |

|  | **Questions relating to baseline confounding only** | | |
| --- | --- | --- | --- |
|  | 1.4. Did the authors use an appropriate analysis method that controlled for all the important confounding domains? | The study employed targeted maximum likelihood estimation (TMLE) combined with an ensemble (super learner) approach, incorporating an extensive list of covariates. This robust methodology is well accepted for minimizing bias from confounding in observational data. Based on this, the study appropriately controlled for key confounders | Y |
|  | 1.5. **If Y/PY to 1.4**: Were confounding domains that were controlled for measured validly and reliably by the variables available in this study? | The data source (CPRD Aurum) is a well-established, high-quality repository of routinely collected primary care data. The covariates—including clinical diagnoses, demographic details, and practice-level characteristics—are measured reliably. Based on this, the study validly and reliably measured the confounding domains | Y |
|  | 1.6. Did the authors control for any post-intervention variables that could have been affected by the intervention? | There is no indication that the analysis adjusted for any variables that might lie on the causal pathway (i.e., post-intervention) since the exposure (consultation mode) and outcome (antibiotic prescribing) are contemporaneously recorded. Based on this, the study avoids introducing bias through adjustment of post-intervention variables. | N |
|  | **Questions relating to baseline and time-varying confounding** | |  |
|  | 1.7. Did the authors use an appropriate analysis method that controlled for all the important confounding domains and for time-varying confounding? |  | NA |
|  | 1.8. **If Y/PY to 1.7**: Were confounding domains that were controlled for measured validly and reliably by the variables available in this study? |  | NA |
|  | **Risk of bias judgement** |  | Low |
|  | Optional: What is the predicted direction of bias due to confounding? |  | Unpredictable |

| **Bias in selection of participants into the study** | | | |
| --- | --- | --- | --- |
|  | 2.1. Was selection of participants into the study (or into the analysis) based on participant characteristics observed after the start of intervention?  **If N/PN to 2.1:** go to 2.4 | The study cohort was defined by pre-specified eligibility criteria (e.g., acceptable data quality, registration within the sampled practices, available IMD linkage) applied before the determination of consultation mode. There is no indication that post-intervention characteristics drove participant selection. Based on this, selection bias related to post-intervention variables is minimized. | N |
|  | 2.2. **If Y/PY to 2.1**: Were the post-intervention variables that influenced selection likely to be associated with intervention?  2.3 **If Y/PY to 2.2**: Were the post-intervention variables that influenced selection likely to be influenced by the outcome or a cause of the outcome? |  | NA  NA |
|  | 2.4. Do start of follow-up and start of intervention coincide for most participants? | For each consultation, the recorded mode (remote or face-to-face) simultaneously defines the exposure and the start of follow-up for that episode. Based on this, the study design ensures that follow-up and intervention start coincide. | Y |
|  | 2.5. **If Y/PY to 2.2 and 2.3, or N/PN to 2.4**: Were adjustment techniques used that are likely to correct for the presence of selection biases? |  | NA |
|  | **Risk of bias judgement** |  | Low |
|  | Optional: What is the predicted direction of bias due to selection of participants into the study? |  | Unpredictable |

| **Bias in classification of interventions** | | | |
| --- | --- | --- | --- |
|  | 3.1 Were intervention groups clearly defined? | The study clearly defines the intervention as the mode of consultation, with remote consultations (via telephone, video, SMS, internet) distinguished from face-to-face (including in-practice, at-home, and mixed consultations, where mixed were conservatively classified as face-to-face in the main analysis). Based on this, the classification is explicit and clear | Y |
|  | 3.2 Was the information used to define intervention groups recorded at the start of the intervention? | Consultation mode was recorded in the consultation table and associated observations at the time of the encounter. Based on this, the intervention classification was determined prospectively at the time of the consultation | Y |
|  | 3.3 Could classification of intervention status have been affected by knowledge of the outcome or risk of the outcome? | The mode of consultation is recorded independently of the subsequent antibiotic prescription decision, and the classification was based on standard coding within CPRD. There is no indication that knowledge of the outcome influenced the classification. Based on this, the study adequately prevents differential misclassification. | N |
|  | **Risk of bias judgement** |  | Low |
|  | Optional: What is the predicted direction of bias due to classification of interventions? |  | Unpredictable |

| **Bias due to deviations from intended interventions** | | | |
| --- | --- | --- | --- |
|  | **If your aim for this study is to assess the effect of assignment to intervention, answer questions 4.1 and 4.2** | |  |
|  | 4.1. Were there deviations from the intended intervention beyond what would be expected in usual practice? |  | Y / PY / PN / N / NI |
|  | 4.2. **If Y/PY to 4.1**: Were these deviations from intended intervention unbalanced between groups *and* likely to have affected the outcome? |  | NA / Y / PY / PN / N / NI |
|  | **If your aim for this study is to assess the effect of starting and adhering to intervention, answer questions 4.3 to 4.6** | |  |
|  | 4.3. Were important co-interventions balanced across intervention groups? | Given that the study is based on routinely recorded data from general practice and that all consultations by GPs were included, any co-interventions (such as additional clinical advice or supportive measures) are assumed to be delivered as part of usual care. The extensive adjustment for patient, clinician, and practice-level factors further supports balance. Based on this, important co-interventions are considered balanced. | Y |
|  | 4.4. Was the intervention implemented successfully for most participants? | The study records consultation mode for all ARI consultations, with only a small number having ambiguous mode (which were defaulted to face-to-face). This consistent recording indicates that the intervention (consultation mode) was implemented successfully. Based on this, the study demonstrates successful implementation. | Y |
|  | 4.5. Did study participants adhere to the assigned intervention regimen? | In this context, ‘adherence’ reflects that the consultation mode as recorded was the mode in which the patient was seen. Since there is no active switching or deviation reported (the consultation mode is fixed at the time of the encounter), it is reasonable to conclude that participants received the intervention as classified. Based on this, adherence is confirmed. | Y |
|  | 4.6. **If N/PN to 4.3, 4.4 or 4.5**: Was an appropriate analysis used to estimate the effect of starting and adhering to the intervention? |  | NA |
|  | **Risk of bias judgement** |  | Low |
|  | Optional: What is the predicted direction of bias due to deviations from the intended interventions? |  | Unpredictable |

| **Bias due to missing data** | | | |
| --- | --- | --- | --- |
|  | 5.1 Were outcome data available for all, or nearly all, participants? | The study utilizes a large, high-quality dataset from CPRD Aurum, and outcome data (antibiotic prescribing) are routinely recorded for each consultation. Only a very small number of patients (fewer than 15 missing IMD data) were excluded. Based on this, outcome data are nearly complete. | Y |
|  | 5.2 Were participants excluded due to missing data on intervention status? | There is no indication that participants were excluded based on missing data regarding consultation mode. Based on this, the study minimizes bias related to missing intervention data. | N |
|  | 5.3 Were participants excluded due to missing data on other variables needed for the analysis? | Apart from the negligible exclusions for missing IMD data, the dataset is comprehensive with regard to the covariates required for adjustment. Based on this, missing data are unlikely to bias the results | N |
|  | 5.4 **If PN/N to 5.1, or Y/PY to 5.2 or 5.3**: Are the proportion of participants and reasons for missing data similar across interventions? |  | NA |
|  | 5.5 **If PN/N to 5.1, or Y/PY to 5.2 or 5.3**: Is there evidence that results were robust to the presence of missing data? |  | NA |
|  | **Risk of bias judgement** |  | Low |
|  | Optional: What is the predicted direction of bias due to missing data? |  | Unpredictable |

| **Bias in measurement of outcomes** | | | |
| --- | --- | --- | --- |
|  | 6.1 Could the outcome measure have been influenced by knowledge of the intervention received? | The outcome—antibiotic prescribing—is recorded objectively in the electronic health records and is not subject to subjective interpretation influenced by consultation mode. Based on this, there is no evidence that outcome measurement was affected by knowledge of the exposure | N |
|  | 6.2 Were outcome assessors aware of the intervention received by study participants? | Outcome data are extracted from routinely recorded electronic records, and those recording the data (or later extracting them) are not influenced by knowledge of the consultation mode at the time of analysis. Based on this, assessor awareness is unlikely to have biased the measurement. | N |
|  | 6.3 Were the methods of outcome assessment comparable across intervention groups? | The methods for recording antibiotic prescriptions are uniform across all consultations, regardless of mode. Based on this, the assessment methods are comparable across groups. | Y |
|  | 6.4 Were any systematic errors in measurement of the outcome related to intervention received? | There is no indication of systematic errors in how antibiotic prescriptions were recorded that could be related to whether a consultation was remote or face-to-face. Based on this, the study appropriately minimizes measurement bias. | N |
|  | **Risk of bias judgement** |  | Low |
|  | Optional: What is the predicted direction of bias due to measurement of outcomes? |  | Unpredictable |

| **Bias in selection of the reported result** | | | |
| --- | --- | --- | --- |
|  | Is the reported effect estimate likely to be selected, on the basis of the results, from... |  |  |
|  | 7.1. ... multiple outcome *measurements* within the outcome domain? | The study clearly pre-specifies antibiotic prescribing as the primary outcome and reports this outcome systematically for both adults and children. There is no indication of selective choice among multiple measurements. Based on this, the risk of selective reporting within the outcome domain is minimal. | N |
|  | 7.2 ... multiple *analyses* of the intervention-outcome relationship? | The authors report both the average treatment effect and the odds ratio using TMLE, and the analytical approach is thoroughly described. There is no evidence that multiple competing analyses were conducted with selective reporting. Based on this, the study avoids this source of bias. | N |
|  | 7.3 ... different *subgroups*? | Subgroup analyses (adults versus children) were pre-specified and reported in full. There is no indication that results were selectively reported from among multiple subgroups. Based on this, selective reporting bias is unlikely. | N |
|  | **Risk of bias judgement** |  | Low |
|  | Optional: What is the predicted direction of bias due to selection of the reported result? |  | Unpredictable |

| **Overall bias** | | | |
| --- | --- | --- | --- |
|  | **Risk of bias judgement** |  | Low |
|  | Optional: What is the overall predicted direction of bias for this outcome? |  | Unpredictable |

# 8: Moving to telehealth antimicrobial stewardship during the Covid-19 pandemic – impact on activity and adherence [60].

|  | **Signalling questions** | **Description** | **Response options** |
| --- | --- | --- | --- |
| **Bias due to confounding** | | | |
|  | 1.1 Is there potential for confounding of the effect of intervention in this study?  **If N/PN to 1.1:** the study can be considered to be at low risk of bias due to confounding and no further signalling questions need be considered | The study is a retrospective audit comparing periods when antimicrobial stewardship (AMS) rounds were delivered face-to-face versus via telehealth (telestewardship). Although the authors report similar hospital activity (measured in occupied bed days) and provide the number of patients referred for PAF review, the before‐and‐after design is inherently susceptible to time‐dependent confounding. Factors such as subtle changes in patient case mix, evolving clinical practices, or other temporal influences (for example, differences in elective surgery volumes and staffing) may not have been fully captured or adjusted for. Based on this, while many key contextual variables were measured, residual confounding remains a concern. | PY |
|  | **If Y/PY to 1.1**: determine whether there is a need to assess time-varying confounding: |  |  |
|  | 1.2. Was the analysis based on splitting participants’ follow up time according to intervention received?  **If N/PN**, answer questions relating to baseline confounding (1.4 to 1.6)  **If Y/PY**, go to question 1.3. |  | NA |
|  | 1.3. Were intervention discontinuations or switches likely to be related to factors that are prognostic for the outcome?  **If N/PN**, answer questions relating to baseline confounding (1.4 to 1.6)  **If Y/PY**, answer questions relating to both baseline and time-varying confounding (1.7 and 1.8) |  | NA |

|  | **Questions relating to baseline confounding only** | | |
| --- | --- | --- | --- |
|  | 1.4. Did the authors use an appropriate analysis method that controlled for all the important confounding domains? | The study compared pre-defined audit metrics (activity and adherence) across two different time periods using standard statistical comparisons (mean ± SD, p-values). Although this approach provides useful insights, it does not employ advanced adjustment methods (e.g., multivariable regression or interrupted time series analysis with adjustment for potential confounders) to fully account for time-dependent changes. Thus, while the analysis is appropriate for a rapid audit, some residual confounding may persist. | Y |
|  | 1.5. **If Y/PY to 1.4**: Were confounding domains that were controlled for measured validly and reliably by the variables available in this study? | Key contextual variables—including the number of patients referred for review and hospital occupied bed days—were routinely recorded and appear to have been measured reliably. However, the possibility of unmeasured confounders (such as differences in patient complexity or subtle changes in care processes over time) remains. Based on this, the measured confounders are valid, but not exhaustive. | Y |
|  | 1.6. Did the authors control for any post-intervention variables that could have been affected by the intervention? | The outcomes (activity and adherence) were measured as part of the same audit process that documented the mode of AMS rounds. There is no evidence that variables measured after the intervention were used for adjustment. Based on this, the study avoids bias from adjusting for post-intervention variables | N |
|  | **Questions relating to baseline and time-varying confounding** | |  |
|  | 1.7. Did the authors use an appropriate analysis method that controlled for all the important confounding domains and for time-varying confounding? |  | NA |
|  | 1.8. **If Y/PY to 1.7**: Were confounding domains that were controlled for measured validly and reliably by the variables available in this study? |  | NA |
|  | **Risk of bias judgement** |  | Moderate |
|  | Optional: What is the predicted direction of bias due to confounding? |  | Unpredictable |

| **Bias in selection of participants into the study** | | | |
| --- | --- | --- | --- |
|  | 2.1. Was selection of participants into the study (or into the analysis) based on participant characteristics observed after the start of intervention?  **If N/PN to 2.1:** go to 2.4 | The audit included all patients referred for Prospective Audit and Feedback (PAF) rounds through the established electronic referral system during the specified time periods. These referrals were generated before the AMS rounds were conducted, and there is no indication that post-intervention characteristics influenced selection. Based on this, the study minimizes selection bias from this source. | N |
|  | 2.2. **If Y/PY to 2.1**: Were the post-intervention variables that influenced selection likely to be associated with intervention?  2.3 **If Y/PY to 2.2**: Were the post-intervention variables that influenced selection likely to be influenced by the outcome or a cause of the outcome? |  | NA  NA |
|  | 2.4. Do start of follow-up and start of intervention coincide for most participants? | For every referral, the timing of the AMS review (whether face-to-face or via telestewardship) marks both the start of the intervention and the subsequent measurement of outcomes (activity and adherence). Based on this, follow-up and intervention start coincide for all cases. | Y |
|  | 2.5. **If Y/PY to 2.2 and 2.3, or N/PN to 2.4**: Were adjustment techniques used that are likely to correct for the presence of selection biases? |  | NA |
|  | **Risk of bias judgement** |  | Low |
|  | Optional: What is the predicted direction of bias due to selection of participants into the study? |  | Unpredictable |

| **Bias in classification of interventions** | | | |
| --- | --- | --- | --- |
|  | 3.1 Were intervention groups clearly defined? | The study clearly distinguishes between two modes of delivering AMS rounds: traditional face-to-face rounds and telestewardship rounds (conducted via telehealth using the EMR and associated applications). The time periods corresponding to each mode are explicitly described. Based on this, the intervention groups are clearly defined. | Y |
|  | 3.2 Was the information used to define intervention groups recorded at the start of the intervention? | The mode of AMS rounds was recorded at the time of each round (using the electronic referral system and EMR documentation), ensuring that the classification of intervention was determined prospectively. Based on this, the recording is appropriately timed. | Y |
|  | 3.3 Could classification of intervention status have been affected by knowledge of the outcome or risk of the outcome? | The decision to use face-to-face or telestewardship was made operationally based on external factors (e.g., COVID-19 incidence, elective surgery volume) and was documented independently of the outcome measures (activity and adherence). Based on this, the risk of bias from outcome knowledge influencing intervention classification is minimal. | N |
|  | **Risk of bias judgement** |  | Low |
|  | Optional: What is the predicted direction of bias due to classification of interventions? |  | Unpredictable |

| **Bias due to deviations from intended interventions** | | | |
| --- | --- | --- | --- |
|  | **If your aim for this study is to assess the effect of assignment to intervention, answer questions 4.1 and 4.2** | |  |
|  | 4.1. Were there deviations from the intended intervention beyond what would be expected in usual practice? |  | Y / PY / PN / N / NI |
|  | 4.2. **If Y/PY to 4.1**: Were these deviations from intended intervention unbalanced between groups *and* likely to have affected the outcome? |  | NA / Y / PY / PN / N / NI |
|  | **If your aim for this study is to assess the effect of starting and adhering to intervention, answer questions 4.3 to 4.6** | |  |
|  | 4.3. Were important co-interventions balanced across intervention groups? | Both face-to-face and telestewardship rounds followed the core protocol for Prospective Audit and Feedback (PAF) rounds, including review of patient charts, laboratory results, radiology, and documentation in the EMR. Although telestewardship rounds included an additional step of making a phone call when management was likely to be significantly affected, this was part of the established protocol. Based on this, co-interventions are systematically applied in both modes. | Y |
|  | 4.4. Was the intervention implemented successfully for most participants? | The study reports that AMS rounds were conducted daily during both face-to-face and telestewardship periods, with clear documentation of advice provided. The adherence to AMS advice remained consistently high (79.1% vs. 80.4%), indicating that the intervention was implemented successfully. Based on this, implementation was effective for most rounds. | Y |
|  | 4.5. Did study participants adhere to the assigned intervention regimen? | Adherence’ in this context is measured as the proportion of treating teams following the AMS advice provided during the rounds. The similar adherence percentages between face-to-face and telestewardship periods indicate that the clinical teams followed the advice as intended. Based on this, adherence to the intervention was high. | Y |
|  | 4.6. **If N/PN to 4.3, 4.4 or 4.5**: Was an appropriate analysis used to estimate the effect of starting and adhering to the intervention? |  | NA |
|  | **Risk of bias judgement** |  | Low |
|  | Optional: What is the predicted direction of bias due to deviations from the intended interventions? |  | Unpredictable |

| **Bias due to missing data** | | | |
| --- | --- | --- | --- |
|  | 5.1 Were outcome data available for all, or nearly all, participants? | The audit includes all eligible AMS rounds during the specified periods, and key outcomes (number of patients reviewed and adherence to advice) are reported with complete summary statistics. There is no indication that substantial outcome data were missing. Based on this, outcome data are available for nearly all rounds | Y |
|  | 5.2 Were participants excluded due to missing data on intervention status? | There is no indication that any AMS rounds were excluded because the mode of delivery (face-to-face vs. telestewardship) was missing; this information is part of the routine documentation. Based on this, exclusion due to missing intervention data is unlikely. | N |
|  | 5.3 Were participants excluded due to missing data on other variables needed for the analysis? | The study does not report any exclusions based on missing data for the key outcomes or contextual variables. Based on this, missing data do not appear to bias the results. | N |
|  | 5.4 **If PN/N to 5.1, or Y/PY to 5.2 or 5.3**: Are the proportion of participants and reasons for missing data similar across interventions? |  | NA |
|  | 5.5 **If PN/N to 5.1, or Y/PY to 5.2 or 5.3**: Is there evidence that results were robust to the presence of missing data? |  | NA |
|  | **Risk of bias judgement** |  | Low |
|  | Optional: What is the predicted direction of bias due to missing data? |  | Unpredictable |

| **Bias in measurement of outcomes** | | | |
| --- | --- | --- | --- |
|  | 6.1 Could the outcome measure have been influenced by knowledge of the intervention received? | The outcomes—namely, the number of patients reviewed during PAF rounds and the adherence to AMS advice—are recorded objectively as part of routine documentation in the EMR and associated systems. There is no evidence that knowledge of the mode of delivery influenced these measurements. Based on this, the outcome measurement is not affected by intervention awareness. | N |
|  | 6.2 Were outcome assessors aware of the intervention received by study participants? | Outcome data were collected through standard audit procedures using routinely recorded electronic systems. Although the AMS team both delivered the intervention and documented outcomes, the objective nature of the data (counts and percentages) minimizes the risk of bias due to assessor awareness. Based on this, the potential for such bias is minimal. | N |
|  | 6.3 Were the methods of outcome assessment comparable across intervention groups? | Both face-to-face and telestewardship rounds employed the same underlying systems (EMR, eReferrals, laboratory applications) and used the same criteria to record activity and adherence. Based on this, the outcome assessment methods were uniformly applied across both modes. | Y |
|  | 6.4 Were any systematic errors in measurement of the outcome related to intervention received? | There is no evidence that the method of recording outcomes systematically differed between the two modes of AMS rounds. Minor procedural differences (such as verifying medication charts via phone when needed) are part of the telestewardship protocol and do not introduce systematic error. Based on this, systematic measurement error is unlikely. | N |
|  | **Risk of bias judgement** |  | Low |
|  | Optional: What is the predicted direction of bias due to measurement of outcomes? |  | Unpredictable |

| **Bias in selection of the reported result** | | | |
| --- | --- | --- | --- |
|  | Is the reported effect estimate likely to be selected, on the basis of the results, from... |  |  |
|  | 7.1. ... multiple outcome *measurements* within the outcome domain? | The study pre-specified its primary audit outcomes—namely, the number of patients reviewed during AMS rounds and the adherence to advice—and reported these outcomes comprehensively for the defined time periods. There is no evidence that effect estimates were selected from among multiple outcome measurements. Based on this, the risk of selective reporting within the outcome domain is minimal. | N |
|  | 7.2 ... multiple *analyses* of the intervention-outcome relationship? | The analysis is straightforward, with direct comparisons of the two periods (face-to-face versus telestewardship) using mean values, standard deviations, and p-values. There is no indication that multiple competing analyses were conducted with selective reporting. Based on this, this source of bias is unlikely. | N |
|  | 7.3 ... different *subgroups*? | The study does not report multiple subgroup analyses; outcomes are aggregated for the entire audit periods. Based on this, selective reporting based on subgroup analyses is unlikely. | N |
|  | **Risk of bias judgement** |  | Low |
|  | Optional: What is the predicted direction of bias due to selection of the reported result? |  | Unpredictable |

| **Overall bias** | | | |
| --- | --- | --- | --- |
|  | **Risk of bias judgement** |  | Moderate |
|  | Optional: What is the overall predicted direction of bias for this outcome? |  | Unpredictable |

# 9: Effect of tele‐COVID rounds and a tele‐stewardship intervention on antibiotic use in COVID‐19 patients admitted to 17 small community hospitals [61].

|  | **Signalling questions** | **Description** | **Response options** |
| --- | --- | --- | --- |
| **Bias due to confounding** | | | |
|  | 1.1 Is there potential for confounding of the effect of intervention in this study?  **If N/PN to 1.1:** the study can be considered to be at low risk of bias due to confounding and no further signalling questions need be considered | The study is a pre–post intervention comparison that examines antibiotic use before and after implementation of tele‐COVID rounds plus a tele‐stewardship intervention. Although the intervention was implemented system-wide, there are notable differences in baseline characteristics between the preintervention and postintervention groups (e.g., preintervention patients were younger, had fewer comorbidities, and differed by race/ethnicity). Moreover, the study design did not include a multivariable adjustment to account for these baseline differences, and the interrupted time-series (ITS) analysis—while useful—relies on a small preintervention sample and makes assumptions that may not fully capture evolving temporal trends. Based on these considerations, there remains a moderate concern for residual confounding. | PY |
|  | **If Y/PY to 1.1**: determine whether there is a need to assess time-varying confounding: |  |  |
|  | 1.2. Was the analysis based on splitting participants’ follow up time according to intervention received?  **If N/PN**, answer questions relating to baseline confounding (1.4 to 1.6)  **If Y/PY**, go to question 1.3. |  | NA |
|  | 1.3. Were intervention discontinuations or switches likely to be related to factors that are prognostic for the outcome?  **If N/PN**, answer questions relating to baseline confounding (1.4 to 1.6)  **If Y/PY**, answer questions relating to both baseline and time-varying confounding (1.7 and 1.8) |  | NA |

|  | **Questions relating to baseline confounding only** | | |
| --- | --- | --- | --- |
|  | 1.4. Did the authors use an appropriate analysis method that controlled for all the important confounding domains? | The study employed univariate comparisons and an ITS analysis to estimate changes in antibiotic use. Although ITS analysis is an accepted method for evaluating interventions over time, the lack of multivariable adjustment to account for marked baseline differences (such as age, comorbidity burden, and other demographic variables) means that not all important confounding domains were fully controlled. Based on this, the methods partly address confounding but leave residual uncertainty. | PY |
|  | 1.5. **If Y/PY to 1.4**: Were confounding domains that were controlled for measured validly and reliably by the variables available in this study? | Key patient characteristics (age, comorbidities, race/ethnicity, and severity measures) and antibiotic use metrics were abstracted from the enterprise data warehouse using standard definitions and are reported with appropriate summary statistics. Although the measurement of these confounders appears valid and reliable, the possibility of unmeasured confounders (e.g., changes in clinical practice over time or other system-level factors) persists | Y |
|  | 1.6. Did the authors control for any post-intervention variables that could have been affected by the intervention? | The outcomes and covariates were measured based on data available from the enterprise data warehouse, and there is no indication that any post-intervention variables (those potentially lying on the causal pathway) were inappropriately adjusted for. Based on this, the study avoids bias due to adjustment for post-intervention variables. | N |
|  | **Questions relating to baseline and time-varying confounding** | |  |
|  | 1.7. Did the authors use an appropriate analysis method that controlled for all the important confounding domains and for time-varying confounding? |  | NA |
|  | 1.8. **If Y/PY to 1.7**: Were confounding domains that were controlled for measured validly and reliably by the variables available in this study? |  | NA |
|  | **Risk of bias judgement** |  | Moderate |
|  | Optional: What is the predicted direction of bias due to confounding? |  | Unpredictable |

| **Bias in selection of participants into the study** | | | |
| --- | --- | --- | --- |
|  | 2.1. Was selection of participants into the study (or into the analysis) based on participant characteristics observed after the start of intervention?  **If N/PN to 2.1:** go to 2.4 | The study included all adult, PCR-positive COVID-19 admissions meeting predefined eligibility criteria (with exclusions limited to routine screening encounters such as maternity and behavioral health) during the two time periods. Selection was based on preintervention criteria as recorded in the enterprise data warehouse, with no evidence that post-intervention characteristics drove selection. Based on this, the risk of bias from participant selection is minimal. | N |
|  | 2.2. **If Y/PY to 2.1**: Were the post-intervention variables that influenced selection likely to be associated with intervention?  2.3 **If Y/PY to 2.2**: Were the post-intervention variables that influenced selection likely to be influenced by the outcome or a cause of the outcome? |  | NA  NA |
|  | 2.4. Do start of follow-up and start of intervention coincide for most participants? | For every included patient, the index date (admission for COVID-19) aligns with the period in which the intervention (tele‐COVID rounds and teleASP surveillance) was or was not active. Thus, the start of follow-up coincides with the intervention period, ensuring that exposure assignment is timely. | Y |
|  | 2.5. **If Y/PY to 2.2 and 2.3, or N/PN to 2.4**: Were adjustment techniques used that are likely to correct for the presence of selection biases? |  | NA |
|  | **Risk of bias judgement** |  | Low |
|  | Optional: What is the predicted direction of bias due to selection of participants into the study? |  | Unpredictable |

| **Bias in classification of interventions** | | | |
| --- | --- | --- | --- |
|  | 3.1 Were intervention groups clearly defined? | The intervention is clearly defined as the implementation of tele‐COVID rounds plus an expanded tele‐stewardship intervention, which was initiated on a specific date and continued thereafter. The preintervention period (March 2020–June 2020) and the postintervention period (July 2020–April 2021) are explicitly delineated. Based on this, the classification of the intervention is clear and unambiguous. | Y |
|  | 3.2 Was the information used to define intervention groups recorded at the start of the intervention? | The intervention status (pre- vs. postimplementation) was assigned according to the admission date and documented changes in the mode of AMS delivery as recorded in the enterprise data warehouse and study protocol. Based on this, the information was recorded at the start of the intervention period. | Y |
|  | 3.3 Could classification of intervention status have been affected by knowledge of the outcome or risk of the outcome? | The timing and method of delivering tele‐COVID rounds and tele‐stewardship are operational decisions made independent of patient outcomes. The classification relies on predetermined dates and institutional protocols rather than on any outcome-related information. Based on this, the risk of differential misclassification is minimal. | N |
|  | **Risk of bias judgement** |  | Low |
|  | Optional: What is the predicted direction of bias due to classification of interventions? |  | Unpredictable |

| **Bias due to deviations from intended interventions** | | | |
| --- | --- | --- | --- |
|  | **If your aim for this study is to assess the effect of assignment to intervention, answer questions 4.1 and 4.2** | |  |
|  | 4.1. Were there deviations from the intended intervention beyond what would be expected in usual practice? |  | Y / PY / PN / N / NI |
|  | 4.2. **If Y/PY to 4.1**: Were these deviations from intended intervention unbalanced between groups *and* likely to have affected the outcome? |  | NA / Y / PY / PN / N / NI |
|  | **If your aim for this study is to assess the effect of starting and adhering to intervention, answer questions 4.3 to 4.6** | |  |
|  | 4.3. Were important co-interventions balanced across intervention groups? | Both preintervention and postintervention periods reflect a consistent operational context in which antimicrobial stewardship (AMS) activities were conducted, with the major difference being the mode of delivery (in-person vs. telehealth). Although the postintervention period incorporated additional teleASP surveillance tools (such as targeted alerts for antibiotic use), these were implemented as part of the intervention package. Based on this, any co-interventions were systematically applied across the study periods. | Y |
|  | 4.4. Was the intervention implemented successfully for most participants? | The study reports detailed workflow descriptions indicating that tele‐COVID rounds and teleASP surveillance were conducted daily and incorporated into the patient review process. Despite some workflow evolution (e.g., the later introduction of a targeted teleASP alert), the intervention was implemented across all 17 small community hospitals. Based on this, the intervention appears to have been implemented successfully. | Y |
|  | 4.5. Did study participants adhere to the assigned intervention regimen? | In this context, ‘adherence’ pertains to the consistent application of the telehealth intervention and the subsequent actions (e.g., early antibiotic discontinuation) recommended by the telehealth team. The study demonstrates significant reductions in antibiotic use and changes in related outcomes that suggest that local care teams largely followed the tele‐COVID rounds’ recommendations. Based on this, adherence is considered high. | Y |
|  | 4.6. **If N/PN to 4.3, 4.4 or 4.5**: Was an appropriate analysis used to estimate the effect of starting and adhering to the intervention? |  | NA |
|  | **Risk of bias judgement** |  | Low |
|  | Optional: What is the predicted direction of bias due to deviations from the intended interventions? |  | Unpredictable |

| **Bias due to missing data** | | | |
| --- | --- | --- | --- |
|  | 5.1 Were outcome data available for all, or nearly all, participants? | Antibiotic use data and other outcomes (e.g., days of therapy per 1000 COVID‐19 patient days, patient transfers, and 30‐day mortality) were abstracted from the Intermountain Enterprise Data Warehouse. Although the preintervention group is notably smaller (n = 120) than the postintervention group (n = 1695), the study does not indicate that data were missing for eligible participants. Based on this, outcome data appear to be nearly complete. | Y |
|  | 5.2 Were participants excluded due to missing data on intervention status? | The mode of intervention (pre- vs. postintervention) was determined by the admission date and documented in the data warehouse; there is no indication that any patients were excluded because of missing information regarding intervention status. Based on this, exclusion due to missing intervention data is unlikely. | N |
|  | 5.3 Were participants excluded due to missing data on other variables needed for the analysis? | The study reports key baseline characteristics and outcomes for both groups, and there is no mention of exclusions based on missing data for these variables. Based on this, missing data are unlikely to have introduced bias. | N |
|  | 5.4 **If PN/N to 5.1, or Y/PY to 5.2 or 5.3**: Are the proportion of participants and reasons for missing data similar across interventions? |  | NA |
|  | 5.5 **If PN/N to 5.1, or Y/PY to 5.2 or 5.3**: Is there evidence that results were robust to the presence of missing data? |  | NA |
|  | **Risk of bias judgement** |  | Low |
|  | Optional: What is the predicted direction of bias due to missing data? |  | Unpredictable |

| **Bias in measurement of outcomes** | | | |
| --- | --- | --- | --- |
|  | 6.1 Could the outcome measure have been influenced by knowledge of the intervention received? | The primary outcome—early antibiotic use measured as mean monthly days of therapy per 1000 COVID‐19 patient days—is derived from objective pharmacy and admission records in the enterprise data warehouse. This measurement is not likely to be influenced by knowledge of whether a patient was admitted pre- or postintervention. Based on this, the risk of bias due to knowledge of the intervention affecting outcome measurement is minimal. | N |
|  | 6.2 Were outcome assessors aware of the intervention received by study participants? | Outcome data were collected from the electronic data warehouse using routine processes. The assessors of these outcomes are not influenced by intervention status because the data collection is automated and objective. | N |
|  | 6.3 Were the methods of outcome assessment comparable across intervention groups? | Both preintervention and postintervention periods used the same data abstraction methods from the enterprise data warehouse and applied the same criteria for defining early antibiotic use. Based on this, the outcome assessment methods were uniformly applied across groups. | Y |
|  | 6.4 Were any systematic errors in measurement of the outcome related to intervention received? | There is no evidence to suggest that the methods of measuring antibiotic use differed systematically between the two periods. Although the ITS analysis had to model a counterfactual trend using a small preintervention sample, the measurement of the outcome itself is consistent. Based on this, systematic measurement error is unlikely. | N |
|  | **Risk of bias judgement** |  | Low |
|  | Optional: What is the predicted direction of bias due to measurement of outcomes? |  | Unpredictable |

| **Bias in selection of the reported result** | | | |
| --- | --- | --- | --- |
|  | Is the reported effect estimate likely to be selected, on the basis of the results, from... |  |  |
|  | 7.1. ... multiple outcome *measurements* within the outcome domain? | The study clearly pre-specified the primary outcome (early antibiotic use for pneumonia, expressed as DOT per 1000 COVID‐19 patient days) and secondary outcomes (e.g., early antibiotic use for any indication, percent of patients receiving no antibiotics, estimated antibiotic days avoided, 30‐day mortality, and hospital transfers). There is no indication that multiple alternative outcome measurements were available from which a favorable effect was selectively reported. | N |
|  | 7.2 ... multiple *analyses* of the intervention-outcome relationship? | The analysis includes both univariate comparisons and an ITS analysis to assess the intervention’s impact. Although the ITS analysis relies on assumptions due to the small preintervention sample, there is no evidence that the investigators conducted multiple competing analyses with selective reporting. | N |
|  | 7.3 ... different *subgroups*? | Subgroup analyses were pre-specified for patients meeting criteria for antibiotic discontinuation and for those with severe pneumonia. The outcomes are reported for these subgroups in addition to the overall analysis, with no indication that results were selectively reported from a large array of subgroup comparisons. | N |
|  | **Risk of bias judgement** |  | Low |
|  | Optional: What is the predicted direction of bias due to selection of the reported result? |  | Unpredictable |

| **Overall bias** | | | |
| --- | --- | --- | --- |
|  | **Risk of bias judgement** |  | Moderate |
|  | Optional: What is the overall predicted direction of bias for this outcome? |  | Unpredictable |

# 10: The effect of Telehealth Antimicrobial Stewardship Program (Tele-ASP) on antimicrobial use in a pediatric intensive care unit: Pre- and post-implementation single center study [62].

|  | **Signalling questions** | **Description** | **Response options** |
| --- | --- | --- | --- |
| **Bias due to confounding** | | | |
|  | 1.1 Is there potential for confounding of the effect of intervention in this study?  **If N/PN to 1.1:** the study can be considered to be at low risk of bias due to confounding and no further signalling questions need be considered | This retrospective cohort study compares antimicrobial use before and after implementation of a Tele-ASP in a pediatric intensive care unit. The investigators included all PICU patients receiving systemic antimicrobials within well-defined time periods and reported key baseline characteristics such as age, sex, PICU length of stay, and mortality. Although there was a statistically significant difference in the need for respiratory support (with a higher proportion in the post-intervention group), other critical clinical parameters were comparable. The authors also performed sensitivity analyses (e.g., seasonal comparisons) that support the stability of the measured outcomes. Based on these factors, potential confounding does not appear to undermine the validity of the intervention effect. | N |
|  | **If Y/PY to 1.1**: determine whether there is a need to assess time-varying confounding: |  |  |
|  | 1.2. Was the analysis based on splitting participants’ follow up time according to intervention received?  **If N/PN**, answer questions relating to baseline confounding (1.4 to 1.6)  **If Y/PY**, go to question 1.3. |  | NA |
|  | 1.3. Were intervention discontinuations or switches likely to be related to factors that are prognostic for the outcome?  **If N/PN**, answer questions relating to baseline confounding (1.4 to 1.6)  **If Y/PY**, answer questions relating to both baseline and time-varying confounding (1.7 and 1.8) |  | NA |

|  | **Questions relating to baseline confounding only** | | |
| --- | --- | --- | --- |
|  | 1.4. Did the authors use an appropriate analysis method that controlled for all the important confounding domains? | The analysis involved appropriate statistical comparisons (Chi-square, t-test, and Mann–Whitney test as applicable) and a sensitivity analysis for seasonal variation. These methods, coupled with the comprehensive reporting of baseline characteristics, indicate that the study adequately addressed and controlled for the most pertinent confounding factors | Y |
|  | 1.5. **If Y/PY to 1.4**: Were confounding domains that were controlled for measured validly and reliably by the variables available in this study? | Patient demographics, clinical parameters, and laboratory data were extracted from detailed medical records and administrative databases. The measurement of these variables appears to be both valid and reliable. Although the retrospective design always leaves some room for unmeasured confounding, the thoroughness of the data collection supports a robust control of the confounding domains. | Y |
|  | 1.6. Did the authors control for any post-intervention variables that could have been affected by the intervention? | Outcomes and covariates were ascertained based on information recorded during routine clinical care and prior to any changes resulting from the intervention. There is no indication that variables influenced by the Tele-ASP were inappropriately adjusted for. This approach minimizes the risk of bias from post-intervention adjustments. | N |
|  | **Questions relating to baseline and time-varying confounding** | |  |
|  | 1.7. Did the authors use an appropriate analysis method that controlled for all the important confounding domains and for time-varying confounding? |  | NA |
|  | 1.8. **If Y/PY to 1.7**: Were confounding domains that were controlled for measured validly and reliably by the variables available in this study? |  | NA |
|  | **Risk of bias judgement** |  | Low |
|  | Optional: What is the predicted direction of bias due to confounding? |  | Unpredictable |

| **Bias in selection of participants into the study** | | | |
| --- | --- | --- | --- |
|  | 2.1. Was selection of participants into the study (or into the analysis) based on participant characteristics observed after the start of intervention?  **If N/PN to 2.1:** go to 2.4 | All pediatric patients admitted to the PICU and receiving systemic antimicrobials during the study period were included based on clear, pre-defined eligibility criteria. Selection was determined solely by admission and antimicrobial use records available from the unit’s archives, with no evidence that post-intervention outcomes influenced the inclusion process | N |
|  | 2.2. **If Y/PY to 2.1**: Were the post-intervention variables that influenced selection likely to be associated with intervention?  2.3 **If Y/PY to 2.2**: Were the post-intervention variables that influenced selection likely to be influenced by the outcome or a cause of the outcome? |  | NA  NA |
|  | 2.4. Do start of follow-up and start of intervention coincide for most participants? | The study design clearly delineates the pre-implementation period from the post-implementation period using fixed calendar dates. As a result, the timing of patient follow-up aligns precisely with the corresponding intervention status, ensuring that exposure assignment is appropriately synchronized with outcome measurement | Y |
|  | 2.5. **If Y/PY to 2.2 and 2.3, or N/PN to 2.4**: Were adjustment techniques used that are likely to correct for the presence of selection biases? |  | NA |
|  | **Risk of bias judgement** |  | Low |
|  | Optional: What is the predicted direction of bias due to selection of participants into the study? |  | Unpredictable |

| **Bias in classification of interventions** | | | |
| --- | --- | --- | --- |
|  | 3.1 Were intervention groups clearly defined? | The intervention (implementation of Tele-ASP) is clearly defined by the initiation date of the ASP rounds (October 8, 2019) with the pre- and post-implementation periods explicitly outlined. The study design distinguishes between the two periods unambiguously. | Y |
|  | 3.2 Was the information used to define intervention groups recorded at the start of the intervention? | Intervention status was assigned based on admission dates and the corresponding operational changes in the ASP, which were documented as part of the hospital’s standard administrative process. This prospective documentation ensures accurate classification. | Y |
|  | 3.3 Could classification of intervention status have been affected by knowledge of the outcome or risk of the outcome? | The classification of patients into pre- and post-Tele-ASP groups was determined solely by the timing of the intervention implementation. There is no indication that outcome data influenced the assignment of intervention status, thereby minimizing the potential for differential misclassification. | N |
|  | **Risk of bias judgement** |  | Low |
|  | Optional: What is the predicted direction of bias due to classification of interventions? |  | Unpredictable |

| **Bias due to deviations from intended interventions** | | | |
| --- | --- | --- | --- |
|  | **If your aim for this study is to assess the effect of assignment to intervention, answer questions 4.1 and 4.2** | |  |
|  | 4.1. Were there deviations from the intended intervention beyond what would be expected in usual practice? |  | Y / PY / PN / N / NI |
|  | 4.2. **If Y/PY to 4.1**: Were these deviations from intended intervention unbalanced between groups *and* likely to have affected the outcome? |  | NA / Y / PY / PN / N / NI |
|  | **If your aim for this study is to assess the effect of starting and adhering to intervention, answer questions 4.3 to 4.6** | |  |
|  | 4.3. Were important co-interventions balanced across intervention groups? | Both study periods reflect the same institutional environment, with the only major difference being the introduction of Tele-ASP. The standard care processes remained largely unchanged, and any co-interventions (such as educational sessions before Tele-ASP launch) were implemented uniformly. This balanced approach helps ensure that differences in outcomes are attributable to the ASP intervention. | Y |
|  | 4.4. Was the intervention implemented successfully for most participants? | The Tele-ASP was instituted according to a well-documented protocol that involved remote participation by infectious diseases specialists, pharmacists, and the PICU team. The high rate of compliance with ASP recommendations (approximately 85.8%) and the significant reduction in antimicrobial use indicate that the intervention was effectively and consistently implemented. | Y |
|  | 4.5. Did study participants adhere to the assigned intervention regimen? | Adherence was assessed by comparing ASP recommendations with subsequent changes in the patient care plans. With full compliance observed in nearly 80% of cases and partial compliance in the remainder, it is evident that the clinical teams adhered closely to the Tele-ASP guidance. | Y |
|  | 4.6. **If N/PN to 4.3, 4.4 or 4.5**: Was an appropriate analysis used to estimate the effect of starting and adhering to the intervention? |  | NA |
|  | **Risk of bias judgement** |  | Low |
|  | Optional: What is the predicted direction of bias due to deviations from the intended interventions? |  | Unpredictable |

| **Bias due to missing data** | | | |
| --- | --- | --- | --- |
|  | 5.1 Were outcome data available for all, or nearly all, participants? | Data on antimicrobial use (expressed as DOT per 1000 patient-days), compliance with ASP recommendations, cost, and relevant clinical outcomes were extracted from comprehensive hospital records and patient charts. The study reports complete data for all 428 patients included, suggesting a negligible impact of missing data. | Y |
|  | 5.2 Were participants excluded due to missing data on intervention status? | The mode of intervention (pre-Tele-ASP versus post-Tele-ASP) was clearly documented based on admission dates and standard operational records. There is no evidence that any patient was excluded because of missing information regarding intervention classification. | N |
|  | 5.3 Were participants excluded due to missing data on other variables needed for the analysis? | Key variables such as demographic details, clinical parameters, and antimicrobial use were available for all participants. No systematic exclusions based on missing data were reported. | N |
|  | 5.4 **If PN/N to 5.1, or Y/PY to 5.2 or 5.3**: Are the proportion of participants and reasons for missing data similar across interventions? |  | NA |
|  | 5.5 **If PN/N to 5.1, or Y/PY to 5.2 or 5.3**: Is there evidence that results were robust to the presence of missing data? |  | NA |
|  | **Risk of bias judgement** |  | Low |
|  | Optional: What is the predicted direction of bias due to missing data? |  | Unpredictable |

| **Bias in measurement of outcomes** | | | |
| --- | --- | --- | --- |
|  | 6.1 Could the outcome measure have been influenced by knowledge of the intervention received? | The primary outcome—antimicrobial use quantified as DOT per 1000 patient-days—is an objective measure derived from prescription records and patient-days. This measurement is unlikely to be affected by knowledge of whether a patient was in the pre- or post-Tele-ASP period | N |
|  | 6.2 Were outcome assessors aware of the intervention received by study participants? | Data extraction was performed retrospectively from hospital records using clearly defined criteria. Outcome assessment was based on objective data, and there is no indication that assessors were influenced by awareness of intervention status. | N |
|  | 6.3 Were the methods of outcome assessment comparable across intervention groups? | The same data collection methods and criteria were applied consistently in both the pre-Tele-ASP and post-Tele-ASP periods. This uniformity ensures that outcome assessments are directly comparable across groups. | Y |
|  | 6.4 Were any systematic errors in measurement of the outcome related to intervention received? | There is no evidence that the procedures for measuring antimicrobial use differed between study periods. The objective nature of the data sources minimizes the risk of systematic error. | N |
|  | **Risk of bias judgement** |  | Low |
|  | Optional: What is the predicted direction of bias due to measurement of outcomes? |  | Unpredictable |

| **Bias in selection of the reported result** | | | |
| --- | --- | --- | --- |
|  | Is the reported effect estimate likely to be selected, on the basis of the results, from... |  |  |
|  | 7.1. ... multiple outcome *measurements* within the outcome domain? | The study pre-specified its primary outcome (change in DOT per 1000 patient-days) and several secondary outcomes (e.g., antimicrobial cost, compliance with ASP recommendations) and reported them in a transparent manner. There is no indication that effect estimates were selectively chosen from among multiple measurements. | N |
|  | 7.2 ... multiple *analyses* of the intervention-outcome relationship? | The analytical approach (including univariate comparisons and sensitivity analyses) is straightforward and clearly described. There is no evidence of multiple competing analyses that might have led to selective reporting of favorable results. | N |
|  | 7.3 ... different *subgroups*? | Although the study reports outcomes by various antimicrobial classes and by admission diagnosis, these subgroup analyses were pre-specified and presented comprehensively, thereby minimizing the risk of selective reporting based on subgroup findings | N |
|  | **Risk of bias judgement** |  | Low |
|  | Optional: What is the predicted direction of bias due to selection of the reported result? |  | Unpredictable |

| **Overall bias** | | | |
| --- | --- | --- | --- |
|  | **Risk of bias judgement** |  | Low |
|  | Optional: What is the overall predicted direction of bias for this outcome? |  | Unpredictable |

# 11: Evaluating the impact of the ICNET® clinical decision support system for antimicrobial stewardship [63].

|  | **Signalling questions** | **Description** | **Response options** |
| --- | --- | --- | --- |
| **Bias due to confounding** | | | |
|  | 1.1 Is there potential for confounding of the effect of intervention in this study?  **If N/PN to 1.1:** the study can be considered to be at low risk of bias due to confounding and no further signalling questions need be considered | The study is a retrospective service evaluation comparing AMS activity before and after the implementation of the ICNET® CDSS. The pre-intervention data (from 2013 and 2014) and the post-intervention data (from a 3-month period in 2016) are separated by several years, during which there were notable changes in AMS staffing, workflow, and IT integration. These temporal differences and system-level changes introduce a substantial risk of confounding that is not fully addressed by the design. | Y |
|  | **If Y/PY to 1.1**: determine whether there is a need to assess time-varying confounding: |  |  |
|  | 1.2. Was the analysis based on splitting participants’ follow up time according to intervention received?  **If N/PN**, answer questions relating to baseline confounding (1.4 to 1.6)  **If Y/PY**, go to question 1.3. |  | NA |
|  | 1.3. Were intervention discontinuations or switches likely to be related to factors that are prognostic for the outcome?  **If N/PN**, answer questions relating to baseline confounding (1.4 to 1.6)  **If Y/PY**, answer questions relating to both baseline and time-varying confounding (1.7 and 1.8) |  | NA |

|  | **Questions relating to baseline confounding only** | | |
| --- | --- | --- | --- |
|  | 1.4. Did the authors use an appropriate analysis method that controlled for all the important confounding domains? | While the authors compared outcomes adjusted for total daily defined doses (DDD) of intravenous antimicrobials, the analysis does not appear to employ advanced multivariable adjustment or methods (e.g., interrupted time-series with robust adjustment for evolving practice patterns) to account for the significant differences in baseline conditions and contextual changes over the several-year interval. Consequently, residual confounding remains a serious concern. | PN |
|  | 1.5. **If Y/PY to 1.4**: Were confounding domains that were controlled for measured validly and reliably by the variables available in this study? | Key variables such as numbers of case reviews, types and numbers of interventions, and antimicrobial consumption (expressed as DDDs/1000 occupied bed days) were extracted from established electronic systems. Although these variables are measured reliably, they may not capture all the factors that changed over time (for example, differences in AMS team composition and clinical practices), leaving important residual confounding unaddressed. | Y |
|  | 1.6. Did the authors control for any post-intervention variables that could have been affected by the intervention? | The outcomes and operational data were collected based on routine logs and reports. There is no indication that variables measured after the intervention were inappropriately adjusted for; however, the inherent differences in the data collection systems between the pre- and post-intervention periods contribute indirectly to confounding | N |
|  | **Questions relating to baseline and time-varying confounding** | |  |
|  | 1.7. Did the authors use an appropriate analysis method that controlled for all the important confounding domains and for time-varying confounding? |  | NA |
|  | 1.8. **If Y/PY to 1.7**: Were confounding domains that were controlled for measured validly and reliably by the variables available in this study? |  | NA |
|  | **Risk of bias judgement** |  | Serious |
|  | Optional: What is the predicted direction of bias due to confounding? |  | Unpredictable |

| **Bias in selection of participants into the study** | | | |
| --- | --- | --- | --- |
|  | 2.1. Was selection of participants into the study (or into the analysis) based on participant characteristics observed after the start of intervention?  **If N/PN to 2.1:** go to 2.4 | The study included all patients subject to AMS review according to predetermined criteria in both the pre-intervention (2013/2014) and post-intervention (2016) periods. Selection was based on standard hospital records and prescribing data, with no evidence that post-intervention outcomes influenced inclusion. | N |
|  | 2.2. **If Y/PY to 2.1**: Were the post-intervention variables that influenced selection likely to be associated with intervention?  2.3 **If Y/PY to 2.2**: Were the post-intervention variables that influenced selection likely to be influenced by the outcome or a cause of the outcome? |  | NA  NA |
|  | 2.4. Do start of follow-up and start of intervention coincide for most participants? | In both study periods, the index for inclusion (i.e., a case being reviewed for AMS purposes) was defined by consistent criteria based on available dispensing and clinical data. The timing of case review was appropriately aligned with the period in which the respective AMS process (pre-CDSS versus post-CDSS) was operational. | Y |
|  | 2.5. **If Y/PY to 2.2 and 2.3, or N/PN to 2.4**: Were adjustment techniques used that are likely to correct for the presence of selection biases? |  | NA |
|  | **Risk of bias judgement** |  | Low |
|  | Optional: What is the predicted direction of bias due to selection of participants into the study? |  | Unpredictable |

| **Bias in classification of interventions** | | | |
| --- | --- | --- | --- |
|  | 3.1 Were intervention groups clearly defined? | The intervention is clearly defined as the implementation of the ICNET® clinical decision support system (CDSS) for antimicrobial stewardship, with the pre-intervention period based on the previous office-based AMS model and the post-intervention period characterized by the CDSS-enabled workflow. The time periods used for comparison are explicitly stated. | Y |
|  | 3.2 Was the information used to define intervention groups recorded at the start of the intervention? | Intervention classification was based on calendar periods and documented changes in the AMS workflow. Pre-intervention data were sourced from the JAC® dispensing system and office-based reviews, whereas post-intervention data were logged automatically via the CDSS. Although the data sources differ, the classification itself was determined prospectively by the institutional change. | Y |
|  | 3.3 Could classification of intervention status have been affected by knowledge of the outcome or risk of the outcome? | The assignment to pre- or post-intervention groups was solely based on the date of the AMS process change. There is no indication that outcome information influenced this classification, minimizing the risk of differential misclassification | N |
|  | **Risk of bias judgement** |  | Low |
|  | Optional: What is the predicted direction of bias due to classification of interventions? |  | Unpredictable |

| **Bias due to deviations from intended interventions** | | | |
| --- | --- | --- | --- |
|  | **If your aim for this study is to assess the effect of assignment to intervention, answer questions 4.1 and 4.2** | |  |
|  | 4.1. Were there deviations from the intended intervention beyond what would be expected in usual practice? |  | Y / PY / PN / N / NI |
|  | 4.2. **If Y/PY to 4.1**: Were these deviations from intended intervention unbalanced between groups *and* likely to have affected the outcome? |  | NA / Y / PY / PN / N / NI |
|  | **If your aim for this study is to assess the effect of starting and adhering to intervention, answer questions 4.3 to 4.6** | |  |
|  | 4.3. Were important co-interventions balanced across intervention groups? | Both the pre- and post-intervention periods were subject to routine AMS activities; however, the post-intervention period benefited from the integration of the CDSS, which altered workflow and enabled bedside reviews. While this represents a change in practice, it was implemented as an integral part of the intervention package. There is no indication that extraneous co-interventions were differentially applied. | Y |
|  | 4.4. Was the intervention implemented successfully for most participants? | The post-intervention period shows clear evidence of CDSS utilization with daily use (mean of 2 h 19 min per day), an increased number of case reviews, and a higher number of recorded clinical interventions. These data suggest that the CDSS was integrated into AMS practice; however, the observed improvements must be interpreted with caution given the concurrent system and staffing changes. | Y |
|  | 4.5. Did study participants adhere to the assigned intervention regimen? | The AMS team’s workflow shifted from an office-based model to a bedside review process supported by the CDSS, and the system logs confirm that patient reviews and interventions were performed as intended. The documentation within the CDSS reflects adherence to the new process. | Y |
|  | 4.6. **If N/PN to 4.3, 4.4 or 4.5**: Was an appropriate analysis used to estimate the effect of starting and adhering to the intervention? |  | NA |
|  | **Risk of bias judgement** |  | Low |
|  | Optional: What is the predicted direction of bias due to deviations from the intended interventions? |  | Unpredictable |

| **Bias due to missing data** | | | |
| --- | --- | --- | --- |
|  | 5.1 Were outcome data available for all, or nearly all, participants? | Data on the number of case reviews, interventions, types of interventions, and antimicrobial consumption (adjusted for DDD) were extracted from established electronic systems. The study does not report any substantial missing data, and the outcomes appear to be comprehensively captured for both periods | Y |
|  | 5.2 Were participants excluded due to missing data on intervention status? | Intervention status was determined by fixed time periods (pre- vs. post-CDSS implementation), and there is no indication that any cases were excluded because of missing information regarding this classification | N |
|  | 5.3 Were participants excluded due to missing data on other variables needed for the analysis? | All necessary variables for the evaluation—such as the number of interventions, patient reviews, and antimicrobial consumption—were available, and no systematic exclusions based on missing data were reported | N |
|  | 5.4 **If PN/N to 5.1, or Y/PY to 5.2 or 5.3**: Are the proportion of participants and reasons for missing data similar across interventions? |  | NA |
|  | 5.5 **If PN/N to 5.1, or Y/PY to 5.2 or 5.3**: Is there evidence that results were robust to the presence of missing data? |  | NA |
|  | **Risk of bias judgement** |  | Low |
|  | Optional: What is the predicted direction of bias due to missing data? |  | Unpredictable |

| **Bias in measurement of outcomes** | | | |
| --- | --- | --- | --- |
|  | 6.1 Could the outcome measure have been influenced by knowledge of the intervention received? | The primary outcomes—including the number of case reviews, interventions, and antimicrobial consumption (DDD/1000 occupied bed days)—are objectively recorded through electronic systems. These measures are unlikely to be influenced by awareness of whether the data originated from the pre- or post-CDSS period. | N |
|  | 6.2 Were outcome assessors aware of the intervention received by study participants? | Data extraction was performed retrospectively from electronic records using predefined criteria. The objective nature of the data collection minimizes the potential for assessor bias related to intervention status. | N |
|  | 6.3 Were the methods of outcome assessment comparable across intervention groups? | Although the pre-intervention period relied on data from the JAC® dispensing system and office-based reviews, and the post-intervention period used the CDSS, the outcomes were standardized (e.g., interventions per 1000 DDDs) to facilitate comparison. However, differences in data capture methods may contribute to residual measurement bias, which is considered in the overall judgment. | Y |
|  | 6.4 Were any systematic errors in measurement of the outcome related to intervention received? | There is no clear evidence of systematic errors in outcome measurement that are directly attributable to the intervention, although the transition from a manual to an automated system might have led to differences in recording practices. This possibility is acknowledged but does not appear to have fundamentally biased the outcome data. | N |
|  | **Risk of bias judgement** |  | Low |
|  | Optional: What is the predicted direction of bias due to measurement of outcomes? |  | Unpredictable |

| **Bias in selection of the reported result** | | | |
| --- | --- | --- | --- |
|  | Is the reported effect estimate likely to be selected, on the basis of the results, from... |  |  |
|  | 7.1. ... multiple outcome *measurements* within the outcome domain? | The study pre-specified a range of outcomes (e.g., number of patients reviewed, total interventions, types of intervention, time burden, and antimicrobial consumption) and reports these outcomes comprehensively. There is no indication that the effect estimates were selectively chosen from among multiple available measurements. | N |
|  | 7.2 ... multiple *analyses* of the intervention-outcome relationship? | The analytical approach is clearly described and consistent with the study’s objectives. Although the evaluation involves comparisons across different time periods, there is no evidence of selective reporting based on multiple analyses of the intervention–outcome relationship. | N |
|  | 7.3 ... different *subgroups*? | Subgroup data (e.g., types of intervention) are reported as part of the overall service evaluation without selective emphasis on any particular subgroup. The reporting is comprehensive and transparent. | N |
|  | **Risk of bias judgement** |  | Low |
|  | Optional: What is the predicted direction of bias due to selection of the reported result? |  | Unpredictable |

| **Overall bias** | | | |
| --- | --- | --- | --- |
|  | **Risk of bias judgement** |  | Serious |
|  | Optional: What is the overall predicted direction of bias for this outcome? |  | Unpredictable |

# 12: The use of telehealth-supported stewardship activities in acute-care and long-term care settings: An implementation effectiveness trial [66].

|  | **Signalling questions** | **Description** | **Response options** |
| --- | --- | --- | --- |
| **Bias due to confounding** | | | |
|  | 1.1 Is there potential for confounding of the effect of intervention in this study?  **If N/PN to 1.1:** the study can be considered to be at low risk of bias due to confounding and no further signalling questions need be considered | The study employs a pre–post design comparing a baseline period (2019–2020) with an intervention period (2021) and uses an interrupted time-series (ITS) analysis with ARIMA models to adjust for secular trends. The evaluation was guided by the RE-AIM framework, and key factors such as patient-days and antimicrobial consumption were measured consistently across periods. Although quasi-experimental designs are inherently at risk for confounding, the rigorous ITS analysis and adjustment for total daily defined doses (DDD) help ensure that differences over time are attributable primarily to the intervention rather than to extraneous temporal factors. Based on this, the study has adequately addressed confounding. | N |
|  | **If Y/PY to 1.1**: determine whether there is a need to assess time-varying confounding: |  |  |
|  | 1.2. Was the analysis based on splitting participants’ follow up time according to intervention received?  **If N/PN**, answer questions relating to baseline confounding (1.4 to 1.6)  **If Y/PY**, go to question 1.3. |  | NA |
|  | 1.3. Were intervention discontinuations or switches likely to be related to factors that are prognostic for the outcome?  **If N/PN**, answer questions relating to baseline confounding (1.4 to 1.6)  **If Y/PY**, answer questions relating to both baseline and time-varying confounding (1.7 and 1.8) |  | NA |

|  | **Questions relating to baseline confounding only** | | |
| --- | --- | --- | --- |
|  | 1.4. Did the authors use an appropriate analysis method that controlled for all the important confounding domains? | The authors utilized ARIMA models within an ITS framework to account for baseline trends and seasonal variations in antibiotic use. This method, along with the standardized measurement of outcomes (e.g., DOT and DASC per 1,000 days present), supports effective control of confounding factors. The consistent application of the RE-AIM framework further confirms that relevant contextual factors were considered, thereby minimizing the risk of confounding. | Y |
|  | 1.5. **If Y/PY to 1.4**: Were confounding domains that were controlled for measured validly and reliably by the variables available in this study? | Outcome and exposure data were extracted from established electronic systems (the Corporate Data Warehouse, VINCI, and barcoding medication administration systems), ensuring valid and reliable measurement of antimicrobial use and patient-days. The use of objective measures (DOT and DASC) minimizes measurement error, and the stable operational context across the two periods supports the reliability of the confounder assessment. | Y |
|  | 1.6. Did the authors control for any post-intervention variables that could have been affected by the intervention? | Intervention status was determined by fixed calendar periods (baseline vs intervention), and outcomes were measured using predefined criteria. There is no indication that variables affected by the intervention were inappropriately adjusted for. The analysis focused on pre-specified outcomes without introducing post-intervention variables into the adjustment model. | N |
|  | **Questions relating to baseline and time-varying confounding** | |  |
|  | 1.7. Did the authors use an appropriate analysis method that controlled for all the important confounding domains and for time-varying confounding? |  | NA |
|  | 1.8. **If Y/PY to 1.7**: Were confounding domains that were controlled for measured validly and reliably by the variables available in this study? |  | NA |
|  | **Risk of bias judgement** |  | Low |
|  | Optional: What is the predicted direction of bias due to confounding? |  | Unpredictable |

| **Bias in selection of participants into the study** | | | |
| --- | --- | --- | --- |
|  | 2.1. Was selection of participants into the study (or into the analysis) based on participant characteristics observed after the start of intervention?  **If N/PN to 2.1:** go to 2.4 | Eligible Veterans Affairs Medical Centers (VAMCs) were identified based on predefined criteria (rural designation, lack of onsite ID support, and absence of local stewardship expertise) from a mandatory antibiotic stewardship survey. All three VAMCs that met these criteria were invited and agreed to participate. The inclusion of all eligible sites and the uniform application of eligibility criteria indicate that selection was not influenced by post-intervention characteristics | N |
|  | 2.2. **If Y/PY to 2.1**: Were the post-intervention variables that influenced selection likely to be associated with intervention?  2.3 **If Y/PY to 2.2**: Were the post-intervention variables that influenced selection likely to be influenced by the outcome or a cause of the outcome? |  | NA  NA |
|  | 2.4. Do start of follow-up and start of intervention coincide for most participants? | Each site’s follow-up was clearly defined by fixed periods: the baseline period (2019–2020) and the intervention period (2021). The timing of data collection for outcomes (e.g., DOT and DASC) directly corresponds to these periods, ensuring that follow-up aligns with the intervention status. | Y |
|  | 2.5. **If Y/PY to 2.2 and 2.3, or N/PN to 2.4**: Were adjustment techniques used that are likely to correct for the presence of selection biases? |  | NA |
|  | **Risk of bias judgement** |  | Low |
|  | Optional: What is the predicted direction of bias due to selection of participants into the study? |  | Unpredictable |

| **Bias in classification of interventions** | | | |
| --- | --- | --- | --- |
|  | 3.1 Were intervention groups clearly defined? | The intervention is defined as the implementation of telehealth-supported stewardship activities (tele-PAF, education, quality monitoring, and remote ID consultation) during 2021, compared with a baseline period (2019–2020) when these activities were not implemented. The use of the RE-AIM framework further clarifies the intervention’s components, ensuring clear group classification. | Y |
|  | 3.2 Was the information used to define intervention groups recorded at the start of the intervention? | Intervention status was assigned by calendar period and documented through institutional records and electronic data systems. The use of the same EMR systems and standardized data extraction methods across both periods ensures that intervention classification was made prospectively and objectively. | Y |
|  | 3.3 Could classification of intervention status have been affected by knowledge of the outcome or risk of the outcome? | The assignment to the baseline or intervention period was solely determined by the date of implementation. There is no indication that outcome information influenced the classification process, minimizing the risk of differential misclassification. | N |
|  | **Risk of bias judgement** |  | Low |
|  | Optional: What is the predicted direction of bias due to classification of interventions? |  | Unpredictable |

| **Bias due to deviations from intended interventions** | | | |
| --- | --- | --- | --- |
|  | **If your aim for this study is to assess the effect of assignment to intervention, answer questions 4.1 and 4.2** | |  |
|  | 4.1. Were there deviations from the intended intervention beyond what would be expected in usual practice? |  | Y / PY / PN / N / NI |
|  | 4.2. **If Y/PY to 4.1**: Were these deviations from intended intervention unbalanced between groups *and* likely to have affected the outcome? |  | NA / Y / PY / PN / N / NI |
|  | **If your aim for this study is to assess the effect of starting and adhering to intervention, answer questions 4.3 to 4.6** | |  |
|  | 4.3. Were important co-interventions balanced across intervention groups? | Both the baseline and intervention periods reflect the same organizational environment within the VAMCs, with the primary difference being the addition of telehealth-supported stewardship activities. There is no indication that other care processes changed differentially between the periods, and the study design (guided by the RE-AIM framework) ensured that co-interventions were monitored and reported consistently. | Y |
|  | 4.4. Was the intervention implemented successfully for most participants? | The intervention was implemented using structured strategies (virtual stakeholder meetings, regular tele-PAF sessions, monthly educational materials, and quality monitoring via REDCap surveys). The fidelity of the implementation was further supported by documented process measures (e.g., frequency of recommendations, time spent, and stakeholder feedback). These elements indicate that the intervention was delivered as intended. | Y |
|  | 4.5. Did study participants adhere to the assigned intervention regimen? | Adherence was assessed through multiple process measures (e.g., number and acceptance of tele-PAF recommendations, documentation of interventions in the EMR, and qualitative feedback from stakeholders). High engagement is reflected in consistent recommendation rates and favorable provider feedback, indicating good adherence to the telehealth-supported stewardship protocol. | Y |
|  | 4.6. **If N/PN to 4.3, 4.4 or 4.5**: Was an appropriate analysis used to estimate the effect of starting and adhering to the intervention? |  | NA |
|  | **Risk of bias judgement** |  | Low |
|  | Optional: What is the predicted direction of bias due to deviations from the intended interventions? |  | Unpredictable |

| **Bias due to missing data** | | | |
| --- | --- | --- | --- |
|  | 5.1 Were outcome data available for all, or nearly all, participants? | Outcome data—including inpatient antibiotic DOT, DASC, and postdischarge DOT—were extracted from the Corporate Data Warehouse using the VA Informatics and Computing Infrastructure (VINCI). Data collection was performed uniformly across the three VAMCs, and no substantial missing data issues were reported. This supports high data completeness | Y |
|  | 5.2 Were participants excluded due to missing data on intervention status? | Intervention status was determined by fixed calendar periods and was recorded as part of the routine data extraction process. There is no evidence that any facility or patient was excluded due to missing information regarding intervention status. | N |
|  | 5.3 Were participants excluded due to missing data on other variables needed for the analysis? | All necessary variables for outcome measurement and adjustment (e.g., patient-days, antibiotic use, discharge data) were available from the CDW and related sources. There is no indication of systematic exclusions related to missing data. | N |
|  | 5.4 **If PN/N to 5.1, or Y/PY to 5.2 or 5.3**: Are the proportion of participants and reasons for missing data similar across interventions? |  | NA |
|  | 5.5 **If PN/N to 5.1, or Y/PY to 5.2 or 5.3**: Is there evidence that results were robust to the presence of missing data? |  | NA |
|  | **Risk of bias judgement** |  | Low |
|  | Optional: What is the predicted direction of bias due to missing data? |  | Unpredictable |

| **Bias in measurement of outcomes** | | | |
| --- | --- | --- | --- |
|  | 6.1 Could the outcome measure have been influenced by knowledge of the intervention received? | Primary outcomes (DOT and DASC per 1,000 days present, as well as postdischarge DOT) were objectively measured using electronic data from standardized systems. The outcomes are based on quantifiable medication administration data and are not subject to subjective interpretation. Thus, outcome measurement is unlikely to have been influenced by knowledge of the intervention. | N |
|  | 6.2 Were outcome assessors aware of the intervention received by study participants? | Outcome data were extracted automatically from electronic databases (e.g., CDW, VINCI) using predefined criteria. Assessors were not involved in data collection in a manner that would reveal intervention status, thereby minimizing the risk of bias due to assessor awareness. | N |
|  | 6.3 Were the methods of outcome assessment comparable across intervention groups? | The same electronic data systems and definitions were used to measure outcomes during both the baseline and intervention periods. This consistency ensures that outcome assessments are comparable across the two periods and across all participating VAMCs | Y |
|  | 6.4 Were any systematic errors in measurement of the outcome related to intervention received? | There is no evidence of systematic differences in how outcomes were measured between the baseline and intervention periods. Although the intervention involved a change in stewardship processes, the data on antibiotic use were captured through automated systems, which minimizes the potential for systematic measurement error. | N |
|  | **Risk of bias judgement** |  | Low |
|  | Optional: What is the predicted direction of bias due to measurement of outcomes? |  | Unpredictable |

| **Bias in selection of the reported result** | | | |
| --- | --- | --- | --- |
|  | Is the reported effect estimate likely to be selected, on the basis of the results, from... |  |  |
|  | 7.1. ... multiple outcome *measurements* within the outcome domain? | The study pre-specified its primary outcome (inpatient antibiotic DOT per 1,000 days present) and secondary outcomes (inpatient DASC and postdischarge DOT per 100 discharges). These outcomes were comprehensively reported, and there is no indication that results were selectively chosen from multiple measurements. | N |
|  | 7.2 ... multiple *analyses* of the intervention-outcome relationship? | The analytical approach was clearly described, employing interrupted time-series analysis with ARIMA models to assess changes over time. There is no evidence that the investigators performed multiple competing analyses with selective outcome reporting. | N |
|  | 7.3 ... different *subgroups*? | Although the study conducted subgroup analyses (e.g., for acute-care versus long-term care settings) as part of its comprehensive evaluation guided by the RE-AIM framework, these were pre-specified and reported transparently without selective emphasis on favorable subgroups | N |
|  | **Risk of bias judgement** |  | Low |
|  | Optional: What is the predicted direction of bias due to selection of the reported result? |  | Unpredictable |

| **Overall bias** | | | |
| --- | --- | --- | --- |
|  | **Risk of bias judgement** |  | Low |
|  | Optional: What is the overall predicted direction of bias for this outcome? |  | Unpredictable |

# 13: Successful Use of Telemedicine Infectious Diseases Consultation With an Antimicrobial Stewardship-Led Staphylococcus aureus Bacteremia Care Bundle [67].

|  | **Signalling questions** | **Description** | **Response options** |
| --- | --- | --- | --- |
| **Bias due to confounding** | | | |
|  | 1.1 Is there potential for confounding of the effect of intervention in this study?  **If N/PN to 1.1:** the study can be considered to be at low risk of bias due to confounding and no further signalling questions need be considered | The study is a retrospective observational evaluation comparing outcomes in patients with Staphylococcus aureus bacteremia who received either standard-of-care (SOC) in-person infectious diseases (ID) consultation or telemedicine (TM) ID consultation. Although there were some baseline differences between the groups—for example, patients in the TM group were older, more likely to be white, and had a lower proportion of complicated bacteremia—the investigators performed multivariable logistic regression to adjust for these differences (including age, comorbidity, bacteremia source, MRSA status, and physiological parameters). Based on these adjustments, potential confounding is adequately controlled. | N |
|  | **If Y/PY to 1.1**: determine whether there is a need to assess time-varying confounding: |  |  |
|  | 1.2. Was the analysis based on splitting participants’ follow up time according to intervention received?  **If N/PN**, answer questions relating to baseline confounding (1.4 to 1.6)  **If Y/PY**, go to question 1.3. |  | NA |
|  | 1.3. Were intervention discontinuations or switches likely to be related to factors that are prognostic for the outcome?  **If N/PN**, answer questions relating to baseline confounding (1.4 to 1.6)  **If Y/PY**, answer questions relating to both baseline and time-varying confounding (1.7 and 1.8) |  | NA |

|  | **Questions relating to baseline confounding only** | | |
| --- | --- | --- | --- |
|  | 1.4. Did the authors use an appropriate analysis method that controlled for all the important confounding domains? | The analysis utilized standard descriptive statistics and multivariable logistic regression models to adjust for baseline demographic, clinical, and laboratory differences. By reporting adjusted odds ratios for 30-day mortality and showing no significant association between the method of ID consultation and clinical outcomes, the study demonstrates that confounding was appropriately addressed. | Y |
|  | 1.5. **If Y/PY to 1.4**: Were confounding domains that were controlled for measured validly and reliably by the variables available in this study? | Key variables—including age, race, Charlson comorbidity index, bacteremia source, MRSA status, time to ID consult, and laboratory measures—were systematically collected from electronic medical records. These variables are standard, objective measures used in clinical research and were measured reliably. Their use in the multivariable analysis supports a robust control for potential confounding. | Y |
|  | 1.6. Did the authors control for any post-intervention variables that could have been affected by the intervention? | Intervention status (SOC versus TM) was determined by the hospital’s existing service model and remained fixed for each patient. Outcome data (bundle adherence and clinical endpoints) were collected independently of any post-intervention changes. There is no evidence that adjustments were made for variables on the causal pathway; therefore, this potential source of bias is minimized. | N |
|  | **Questions relating to baseline and time-varying confounding** | |  |
|  | 1.7. Did the authors use an appropriate analysis method that controlled for all the important confounding domains and for time-varying confounding? |  | NA |
|  | 1.8. **If Y/PY to 1.7**: Were confounding domains that were controlled for measured validly and reliably by the variables available in this study? |  | NA |
|  | **Risk of bias judgement** |  | Low |
|  | Optional: What is the predicted direction of bias due to confounding? |  | Unpredictable |

| **Bias in selection of participants into the study** | | | |
| --- | --- | --- | --- |
|  | 2.1. Was selection of participants into the study (or into the analysis) based on participant characteristics observed after the start of intervention?  **If N/PN to 2.1:** go to 2.4 | Participants were selected based on clear, pre-specified eligibility criteria (i.e., first occurrence of SAB during the study period, with defined exclusions such as early death, leaving against medical advice, or transfer prior to definitive treatment). These criteria were applied uniformly regardless of the mode of ID consultation. There is no indication that post-intervention characteristics influenced the selection process | N |
|  | 2.2. **If Y/PY to 2.1**: Were the post-intervention variables that influenced selection likely to be associated with intervention?  2.3 **If Y/PY to 2.2**: Were the post-intervention variables that influenced selection likely to be influenced by the outcome or a cause of the outcome? |  | NA  NA |
|  | 2.4. Do start of follow-up and start of intervention coincide for most participants? | For every patient, the index blood culture triggering the SAB bundle was used to define the start of follow-up. All patients received the care bundle as part of routine clinical practice according to hospital protocols, ensuring that the measurement of outcomes is appropriately aligned with the intervention period. | Y |
|  | 2.5. **If Y/PY to 2.2 and 2.3, or N/PN to 2.4**: Were adjustment techniques used that are likely to correct for the presence of selection biases? |  | NA |
|  | **Risk of bias judgement** |  | Low |
|  | Optional: What is the predicted direction of bias due to selection of participants into the study? |  | Unpredictable |

| **Bias in classification of interventions** | | | |
| --- | --- | --- | --- |
|  | 3.1 Were intervention groups clearly defined? | The two groups are clearly defined by the mode of ID consultation delivery. The SOC group comprises patients receiving in-person ID consultation at hospitals with onsite consultants, whereas the TM group comprises patients from hospitals without onsite ID consultants who received telemedicine-based ID consultations. This clear delineation ensures that intervention classification is unambiguous | Y |
|  | 3.2 Was the information used to define intervention groups recorded at the start of the intervention? | Intervention status was determined based on hospital assignment and recorded prospectively through the clinical and administrative systems. The study’s design and flow diagram (CONSORT diagram) confirm that patients were allocated to groups based on the available service model at the time of admission. | Y |
|  | 3.3 Could classification of intervention status have been affected by knowledge of the outcome or risk of the outcome? | The classification was made based on the hospital’s operational model (onsite vs. telemedicine) and was not influenced by subsequent outcomes. There is no evidence that outcome knowledge affected the assignment of patients to either group. | N |
|  | **Risk of bias judgement** |  | Low |
|  | Optional: What is the predicted direction of bias due to classification of interventions? |  | Unpredictable |

| **Bias due to deviations from intended interventions** | | | |
| --- | --- | --- | --- |
|  | **If your aim for this study is to assess the effect of assignment to intervention, answer questions 4.1 and 4.2** | |  |
|  | 4.1. Were there deviations from the intended intervention beyond what would be expected in usual practice? |  | Y / PY / PN / N / NI |
|  | 4.2. **If Y/PY to 4.1**: Were these deviations from intended intervention unbalanced between groups *and* likely to have affected the outcome? |  | NA / Y / PY / PN / N / NI |
|  | **If your aim for this study is to assess the effect of starting and adhering to intervention, answer questions 4.3 to 4.6** | |  |
|  | 4.3. Were important co-interventions balanced across intervention groups? | Both groups received the same SAB care bundle, and the only difference was the method of ID consultation (in-person vs. telemedicine). All patients, regardless of consultation modality, were managed under the same bundle protocol. This uniformity minimizes the risk that co-interventions would differentially affect the outcomes. | Y |
|  | 4.4. Was the intervention implemented successfully for most participants? | The SAB bundle was implemented consistently across all participating hospitals, with documented processes for both SOC and TM ID consultation. Process measures (such as the time to consult and adherence to bundle components) were collected, and overall bundle adherence rates were high and similar between groups. This indicates successful implementation. | Y |
|  | 4.5. Did study participants adhere to the assigned intervention regimen? | Patients received the SAB bundle according to their hospital’s standard protocol based on the available consultation modality. There is no indication of deviations from the assigned method of ID consultation; patients were managed as intended within each group. | Y |
|  | 4.6. **If N/PN to 4.3, 4.4 or 4.5**: Was an appropriate analysis used to estimate the effect of starting and adhering to the intervention? |  | NA |
|  | **Risk of bias judgement** |  | Low |
|  | Optional: What is the predicted direction of bias due to deviations from the intended interventions? |  | Unpredictable |

| **Bias due to missing data** | | | |
| --- | --- | --- | --- |
|  | 5.1 Were outcome data available for all, or nearly all, participants? | The study reports outcomes for 738 patients with SAB, and data on bundle adherence, mortality, readmission rates, persistent bacteremia, and time to culture clearance were obtained from comprehensive electronic medical records. Exclusions were clearly described and applied consistently. Data completeness is high | Y |
|  | 5.2 Were participants excluded due to missing data on intervention status? | Intervention status (SOC vs. TM) was determined based on hospital assignment and was documented for all patients. There is no evidence that missing data on intervention status led to exclusions. | N |
|  | 5.3 Were participants excluded due to missing data on other variables needed for the analysis? | All essential variables (demographic, clinical, microbiological, and outcome data) were available for the patients included in the analysis. Exclusions were limited to predetermined criteria (e.g., SAB recurrence, early death) and not due to missing data | N |
|  | 5.4 **If PN/N to 5.1, or Y/PY to 5.2 or 5.3**: Are the proportion of participants and reasons for missing data similar across interventions? |  | NA |
|  | 5.5 **If PN/N to 5.1, or Y/PY to 5.2 or 5.3**: Is there evidence that results were robust to the presence of missing data? |  | NA |
|  | **Risk of bias judgement** |  | Low |
|  | Optional: What is the predicted direction of bias due to missing data? |  | Unpredictable |

| **Bias in measurement of outcomes** | | | |
| --- | --- | --- | --- |
|  | 6.1 Could the outcome measure have been influenced by knowledge of the intervention received? | Primary outcomes, including overall bundle adherence, individual bundle component adherence, hospital mortality, 30-day mortality, 30-day SAB-related readmission, persistent bacteremia, and days to culture clearance, were based on objective data extracted from electronic medical records and standardized quality assurance systems. These outcomes are not subject to subjective interpretation, and there is no indication that knowledge of the intervention mode influenced their measurement. | N |
|  | 6.2 Were outcome assessors aware of the intervention received by study participants? | Outcome data were collected as part of routine clinical care and quality monitoring. The review and adjudication of bundle nonadherence and SAB-related readmissions were performed by independent ID providers, using majority vote when needed, which minimizes bias from assessor awareness. | N |
|  | 6.3 Were the methods of outcome assessment comparable across intervention groups? | Both the SOC and TM groups had their outcomes measured using the same SAB bundle criteria and definitions. Data extraction methods and adjudication processes were consistent across groups, ensuring that outcome assessment was directly comparable | Y |
|  | 6.4 Were any systematic errors in measurement of the outcome related to intervention received? | There is no evidence of systematic differences in how outcomes were measured between the SOC and TM groups. The use of standardized definitions for bundle adherence and objective clinical endpoints minimizes the risk of systematic measurement error. | N |
|  | **Risk of bias judgement** |  | Low |
|  | Optional: What is the predicted direction of bias due to measurement of outcomes? |  | Unpredictable |

| **Bias in selection of the reported result** | | | |
| --- | --- | --- | --- |
|  | Is the reported effect estimate likely to be selected, on the basis of the results, from... |  |  |
|  | 7.1. ... multiple outcome *measurements* within the outcome domain? | The study pre-specified its primary outcome (overall SAB bundle adherence) and key secondary outcomes (individual bundle component adherence, hospital mortality, 30-day mortality, readmission rates, persistent bacteremia, and time to culture clearance). All these outcomes were reported comprehensively, and there is no indication that selective reporting occurred | N |
|  | 7.2 ... multiple *analyses* of the intervention-outcome relationship? | The analytical approach was clearly described, with both univariate comparisons and multivariable logistic regression used to assess the association between consultation method and 30-day mortality, among other outcomes. There is no evidence that multiple competing analyses were performed with selective reporting. | N |
|  | 7.3 ... different *subgroups*? | Subgroup analyses (e.g., separate evaluations of bundle component adherence) were pre-specified and reported for transparency. There is no indication that outcomes were selectively reported from among various subgroup analyses. | N |
|  | **Risk of bias judgement** |  | Low |
|  | Optional: What is the predicted direction of bias due to selection of the reported result? |  | Unpredictable |

| **Overall bias** | | | |
| --- | --- | --- | --- |
|  | **Risk of bias judgement** |  | Low |
|  | Optional: What is the overall predicted direction of bias for this outcome? |  | Unpredictable |

# 14: Antibiotic Prescribing for Acute Respiratory Tract Infections During Telemedicine Visits Within a Pediatric Primary Care Network [68].

|  | **Signalling questions** | **Description** | **Response options** |
| --- | --- | --- | --- |
| **Bias due to confounding** | | | |
|  | 1.1 Is there potential for confounding of the effect of intervention in this study?  **If N/PN to 1.1:** the study can be considered to be at low risk of bias due to confounding and no further signalling questions need be considered | The study compares ARTI visits managed via telemedicine versus in-person care during a 6-month period in 2020 and also includes historical data from 2018–2019 for context. Although all practices share a common EHR and inclusion criteria were applied uniformly, the study design is observational and not randomized. Patients who choose telemedicine may differ systematically from those who attend in-person visits (for example, in severity of illness, parental preference, or accessibility), and external factors such as the stay-at-home order and gradual reopening may have influenced these patterns. While the authors report high rates of guideline-concordant management in both settings, residual confounding due to differences in patient populations and secular trends remains a concern. | PY |
|  | **If Y/PY to 1.1**: determine whether there is a need to assess time-varying confounding: |  |  |
|  | 1.2. Was the analysis based on splitting participants’ follow up time according to intervention received?  **If N/PN**, answer questions relating to baseline confounding (1.4 to 1.6)  **If Y/PY**, go to question 1.3. |  | NA |
|  | 1.3. Were intervention discontinuations or switches likely to be related to factors that are prognostic for the outcome?  **If N/PN**, answer questions relating to baseline confounding (1.4 to 1.6)  **If Y/PY**, answer questions relating to both baseline and time-varying confounding (1.7 and 1.8) |  | NA |

|  | **Questions relating to baseline confounding only** | | |
| --- | --- | --- | --- |
|  | 1.4. Did the authors use an appropriate analysis method that controlled for all the important confounding domains? | The primary analyses rely on chi-square tests and descriptive statistics to compare antibiotic prescribing and diagnostic patterns. Although these methods are useful for summarizing trends, there is limited adjustment for potential confounders (such as patient demographics or clinical severity) in the reported analyses. This limited adjustment suggests that not all important confounding factors have been fully controlled. | PN |
|  | 1.5. **If Y/PY to 1.4**: Were confounding domains that were controlled for measured validly and reliably by the variables available in this study? | Key variables (e.g., visit type, diagnosis codes, and antibiotic prescription status) were extracted from the shared EHR, which is a reliable data source. However, while the measurement of these factors is valid, the observational design and absence of detailed multivariable adjustment mean that residual confounding may persist. | Y |
|  | 1.6. Did the authors control for any post-intervention variables that could have been affected by the intervention? | Intervention status was determined solely by the mode of visit (telemedicine vs. in-person) and by fixed calendar periods. Outcome measures were derived from objective EHR data and were not adjusted based on variables that lie on the causal pathway. Thus, there is no evidence that post-intervention variables were inappropriately adjusted for | N |
|  | **Questions relating to baseline and time-varying confounding** | |  |
|  | 1.7. Did the authors use an appropriate analysis method that controlled for all the important confounding domains and for time-varying confounding? |  | NA |
|  | 1.8. **If Y/PY to 1.7**: Were confounding domains that were controlled for measured validly and reliably by the variables available in this study? |  | NA |
|  | **Risk of bias judgement** |  | Moderate |
|  | Optional: What is the predicted direction of bias due to confounding? |  | Unpredictable |

| **Bias in selection of participants into the study** | | | |
| --- | --- | --- | --- |
|  | 2.1. Was selection of participants into the study (or into the analysis) based on participant characteristics observed after the start of intervention?  **If N/PN to 2.1:** go to 2.4 | The study includes all ARTI visits from 47 practices in a large pediatric primary care network during the specified time periods. Eligibility was based on objective, pre-specified ICD-10 codes and EHR data, and there is no indication that participant selection was influenced by post-intervention characteristics. | N |
|  | 2.2. **If Y/PY to 2.1**: Were the post-intervention variables that influenced selection likely to be associated with intervention?  2.3 **If Y/PY to 2.2**: Were the post-intervention variables that influenced selection likely to be influenced by the outcome or a cause of the outcome? |  | NA  NA |
|  | 2.4. Do start of follow-up and start of intervention coincide for most participants? | For the 2020 data, the follow-up period (April 1–September 30) is clearly defined, and the mode of the visit (telemedicine or in-person) is recorded at the time of the encounter. This ensures that the measurement of outcomes is correctly aligned with the intervention (i.e., the mode of care delivery). | Y |
|  | 2.5. **If Y/PY to 2.2 and 2.3, or N/PN to 2.4**: Were adjustment techniques used that are likely to correct for the presence of selection biases? |  | NA |
|  | **Risk of bias judgement** |  | Low |
|  | Optional: What is the predicted direction of bias due to selection of participants into the study? |  | Unpredictable |

| **Bias in classification of interventions** | | | |
| --- | --- | --- | --- |
|  | 3.1 Were intervention groups clearly defined? | Intervention groups are defined by the mode of visit—telemedicine versus in-person. The study clearly reports the proportion of ARTI visits conducted via telemedicine each month and provides detailed descriptive statistics, ensuring clear classification of the exposure. | Y |
|  | 3.2 Was the information used to define intervention groups recorded at the start of the intervention? | The mode of visit is recorded in the EHR at the time of each encounter, ensuring that classification is made prospectively and objectively. | Y |
|  | 3.3 Could classification of intervention status have been affected by knowledge of the outcome or risk of the outcome? | The classification into telemedicine or in-person groups is based solely on the visit modality as recorded in the EHR and is not influenced by subsequent outcomes, thereby minimizing the potential for differential misclassification. | N |
|  | **Risk of bias judgement** |  | Low |
|  | Optional: What is the predicted direction of bias due to classification of interventions? |  | Unpredictable |

| **Bias due to deviations from intended interventions** | | | |
| --- | --- | --- | --- |
|  | **If your aim for this study is to assess the effect of assignment to intervention, answer questions 4.1 and 4.2** | |  |
|  | 4.1. Were there deviations from the intended intervention beyond what would be expected in usual practice? |  | Y / PY / PN / N / NI |
|  | 4.2. **If Y/PY to 4.1**: Were these deviations from intended intervention unbalanced between groups *and* likely to have affected the outcome? |  | NA / Y / PY / PN / N / NI |
|  | **If your aim for this study is to assess the effect of starting and adhering to intervention, answer questions 4.3 to 4.6** | |  |
|  | 4.3. Were important co-interventions balanced across intervention groups? | Both telemedicine and in-person visits occurred within the same primary care network and under similar operational and clinical conditions. Although the pandemic context (e.g., stay-at-home orders) may have influenced overall visit volumes, there is no evidence that additional co-interventions were applied differentially between the two groups. | Y |
|  | 4.4. Was the intervention implemented successfully for most participants? | The study reports comprehensive data on ARTI visits and antibiotic prescribing across 47 practices, with clear documentation of visit modality and associated antibiotic management. This indicates that the ‘intervention’ (i.e., the use of telemedicine for ARTI visits) was implemented as part of routine care in a manner that was successfully captured by the EHR. | Y |
|  | 4.5. Did study participants adhere to the assigned intervention regimen? | Patients received care by telemedicine or in-person as determined by the practice setting and scheduling. There is no evidence of deviation from the recorded mode of visit. Thus, adherence to the assigned ‘intervention’ is ascertained by the EHR records | Y |
|  | 4.6. **If N/PN to 4.3, 4.4 or 4.5**: Was an appropriate analysis used to estimate the effect of starting and adhering to the intervention? |  | NA |
|  | **Risk of bias judgement** |  | Low |
|  | Optional: What is the predicted direction of bias due to deviations from the intended interventions? |  | Unpredictable |

| **Bias due to missing data** | | | |
| --- | --- | --- | --- |
|  | 5.1 Were outcome data available for all, or nearly all, participants? | Data were extracted from a shared EHR system across 47 practices, and outcome variables (e.g., antibiotic prescribing, diagnostic codes) were available for nearly all ARTI visits. There is no indication that missing data significantly affected the analyses. | Y |
|  | 5.2 Were participants excluded due to missing data on intervention status? | Intervention status (telemedicine vs. in-person) was recorded as part of the routine EHR documentation, and no exclusions were reported due to missing data on this variable. | N |
|  | 5.3 Were participants excluded due to missing data on other variables needed for the analysis? | Exclusion criteria were applied uniformly, and key variables (e.g., diagnosis, antibiotic prescription) were available from the EHR for the vast majority of visits | N |
|  | 5.4 **If PN/N to 5.1, or Y/PY to 5.2 or 5.3**: Are the proportion of participants and reasons for missing data similar across interventions? |  | NA |
|  | 5.5 **If PN/N to 5.1, or Y/PY to 5.2 or 5.3**: Is there evidence that results were robust to the presence of missing data? |  | NA |
|  | **Risk of bias judgement** |  | Low |
|  | Optional: What is the predicted direction of bias due to missing data? |  | Unpredictable |

| **Bias in measurement of outcomes** | | | |
| --- | --- | --- | --- |
|  | 6.1 Could the outcome measure have been influenced by knowledge of the intervention received? | The primary outcomes—antibiotic prescribing and guideline-concordant management—are objectively captured through EHR data. These outcomes are determined by established coding systems (ICD-10) and pharmacy records and are not subject to subjective interpretation influenced by awareness of visit modality. | N |
|  | 6.2 Were outcome assessors aware of the intervention received by study participants? | Outcome data were extracted automatically from the shared EHR without manual review that could be biased by knowledge of the intervention. Thus, outcome assessors were effectively blinded. | N |
|  | 6.3 Were the methods of outcome assessment comparable across intervention groups? | Both telemedicine and in-person visits were assessed using the same diagnostic codes and prescription data from the shared EHR system. This ensures that outcome measurement is consistent and comparable across groups. | Y |
|  | 6.4 Were any systematic errors in measurement of the outcome related to intervention received? | There is no evidence that the change in care delivery modality (telemedicine versus in-person) introduced systematic differences in the measurement of outcomes. The objective nature of EHR data minimizes the risk of such errors. | N |
|  | **Risk of bias judgement** |  | Low |
|  | Optional: What is the predicted direction of bias due to measurement of outcomes? |  | Unpredictable |

| **Bias in selection of the reported result** | | | |
| --- | --- | --- | --- |
|  | Is the reported effect estimate likely to be selected, on the basis of the results, from... |  |  |
|  | 7.1. ... multiple outcome *measurements* within the outcome domain? | The study pre-specified primary and secondary outcomes (guideline-concordant antibiotic management, diagnostic patterns, and antibiotic prescribing rates) and reported these outcomes comprehensively. There is no indication of selective outcome reporting. | N |
|  | 7.2 ... multiple *analyses* of the intervention-outcome relationship? | The analyses were conducted using chi-square tests and descriptive statistics with clearly defined comparisons over time and between visit types. There is no evidence that multiple competing analyses were performed with selective emphasis on favorable results. | N |
|  | 7.3 ... different *subgroups*? | Although the study provides descriptive trends over time (e.g., month-by-month changes), subgroup analyses were pre-specified (for instance, for visits with sinusitis and viral ARTIs) and reported transparently. There is no indication of selective reporting from among multiple subgroup analyses. | N |
|  | **Risk of bias judgement** |  | Low |
|  | Optional: What is the predicted direction of bias due to selection of the reported result? |  | Unpredictable |

| **Overall bias** | | | |
| --- | --- | --- | --- |
|  | **Risk of bias judgement** |  | Moderate |
|  | Optional: What is the overall predicted direction of bias for this outcome? |  | Unpredictable |

# 15: Antibiotic Prescribing Patterns for Sinusitis Within a Direct-to-Consumer Virtual Urgent Care [69].

|  | **Signalling questions** | **Description** | **Response options** |
| --- | --- | --- | --- |
| **Bias due to confounding** | | | |
|  | 1.1 Is there potential for confounding of the effect of intervention in this study?  **If N/PN to 1.1:** the study can be considered to be at low risk of bias due to confounding and no further signalling questions need be considered | This retrospective cohort study compares antibiotic prescribing for acute sinusitis between patients seen in a virtual urgent care (VUC) setting and a control group of sequential patients seen in a traditional urgent care (TUC). Although inclusion criteria were clearly defined using ICD-10 codes, provider heterogeneity is present—VUC visits were managed by academic emergency physicians, whereas the TUC group was primarily managed by community-based nurse practitioners or physician assistants with some family practice physicians. Such differences in provider type and potential differences in patient characteristics (e.g., severity of illness or healthcare-seeking behavior) may act as confounders. Moreover, the control group was selected as a block of sequential encounters, which may not fully capture the variability in the TUC population. Although the study reports descriptive comparisons, limited multivariable adjustment is provided. These factors contribute to a residual risk of confounding. | PY |
|  | **If Y/PY to 1.1**: determine whether there is a need to assess time-varying confounding: |  |  |
|  | 1.2. Was the analysis based on splitting participants’ follow up time according to intervention received?  **If N/PN**, answer questions relating to baseline confounding (1.4 to 1.6)  **If Y/PY**, go to question 1.3. |  | NA |
|  | 1.3. Were intervention discontinuations or switches likely to be related to factors that are prognostic for the outcome?  **If N/PN**, answer questions relating to baseline confounding (1.4 to 1.6)  **If Y/PY**, answer questions relating to both baseline and time-varying confounding (1.7 and 1.8) |  | NA |

|  | **Questions relating to baseline confounding only** | | |
| --- | --- | --- | --- |
|  | 1.4. Did the authors use an appropriate analysis method that controlled for all the important confounding domains? | The analysis primarily relies on Fisher’s exact tests and descriptive statistics to compare antibiotic prescribing rates between VUC and TUC groups. There is no indication that multivariable methods were employed to adjust for differences in provider type or patient characteristics beyond the basic inclusion/exclusion criteria. Thus, while objective data were used, the analysis does not fully account for potential confounding factors, leaving some residual confounding unaddressed. | PN |
|  | 1.5. **If Y/PY to 1.4**: Were confounding domains that were controlled for measured validly and reliably by the variables available in this study? | Key variables such as diagnosis (acute sinusitis), antibiotic prescription status, age, and sex were extracted from the electronic health record (EHR) and are likely to have been measured accurately. However, important confounders—such as provider type and patient clinical severity—were not fully adjusted for, which contributes to the overall residual confounding. | Y |
|  | 1.6. Did the authors control for any post-intervention variables that could have been affected by the intervention? | The classification into VUC versus TUC groups was made based on the care delivery model as recorded in the EHR at the time of the visit. There is no evidence that outcome data or variables lying on the causal pathway were used for adjustment | N |
|  | **Questions relating to baseline and time-varying confounding** | |  |
|  | 1.7. Did the authors use an appropriate analysis method that controlled for all the important confounding domains and for time-varying confounding? |  | NA |
|  | 1.8. **If Y/PY to 1.7**: Were confounding domains that were controlled for measured validly and reliably by the variables available in this study? |  | NA |
|  | **Risk of bias judgement** |  | Moderate |
|  | Optional: What is the predicted direction of bias due to confounding? |  | Unpredictable |

| **Bias in selection of participants into the study** | | | |
| --- | --- | --- | --- |
|  | 2.1. Was selection of participants into the study (or into the analysis) based on participant characteristics observed after the start of intervention?  **If N/PN to 2.1:** go to 2.4 | All adult patients with a discharge diagnosis of acute sinusitis from the virtual urgent care (VUC) were included, and a block of 100 sequential encounters from traditional urgent care (TUC) was selected as the control group. Although the control group was chosen as a convenience sample, the selection was based on pre-specified diagnostic criteria, and there is no indication that participants were selected based on outcomes or post-visit characteristics. | N |
|  | 2.2. **If Y/PY to 2.1**: Were the post-intervention variables that influenced selection likely to be associated with intervention?  2.3 **If Y/PY to 2.2**: Were the post-intervention variables that influenced selection likely to be influenced by the outcome or a cause of the outcome? |  | NA  NA |
|  | 2.4. Do start of follow-up and start of intervention coincide for most participants? | For each encounter, the visit modality (telemedicine or in-person) was recorded at the time of the encounter in the EHR. This ensures that the timing of outcome measurement coincides with the mode of care delivery. | Y |
|  | 2.5. **If Y/PY to 2.2 and 2.3, or N/PN to 2.4**: Were adjustment techniques used that are likely to correct for the presence of selection biases? |  | NA |
|  | **Risk of bias judgement** |  | Low |
|  | Optional: What is the predicted direction of bias due to selection of participants into the study? |  | Unpredictable |

| **Bias in classification of interventions** | | | |
| --- | --- | --- | --- |
|  | 3.1 Were intervention groups clearly defined? | The study clearly distinguishes between virtual urgent care (VUC) visits and traditional urgent care (TUC) visits based on the delivery modality recorded in the EHR. This classification is unambiguous and directly reflects the intended exposure. | Y |
|  | 3.2 Was the information used to define intervention groups recorded at the start of the intervention? | Visit modality was recorded as part of routine clinical documentation in the shared EHR system. This information was captured contemporaneously at the time of the encounter, ensuring accurate classification. | Y |
|  | 3.3 Could classification of intervention status have been affected by knowledge of the outcome or risk of the outcome? | Classification into VUC and TUC groups was based solely on the method of encounter (video/telephone vs. in-person), which is objectively documented. There is no evidence that knowledge of the outcome influenced how visits were classified. | N |
|  | **Risk of bias judgement** |  | Low |
|  | Optional: What is the predicted direction of bias due to classification of interventions? |  | Unpredictable |

| **Bias due to deviations from intended interventions** | | | |
| --- | --- | --- | --- |
|  | **If your aim for this study is to assess the effect of assignment to intervention, answer questions 4.1 and 4.2** | |  |
|  | 4.1. Were there deviations from the intended intervention beyond what would be expected in usual practice? |  | Y / PY / PN / N / NI |
|  | 4.2. **If Y/PY to 4.1**: Were these deviations from intended intervention unbalanced between groups *and* likely to have affected the outcome? |  | NA / Y / PY / PN / N / NI |
|  | **If your aim for this study is to assess the effect of starting and adhering to intervention, answer questions 4.3 to 4.6** | |  |
|  | 4.3. Were important co-interventions balanced across intervention groups? | Both VUC and TUC groups were managed within the same pediatric primary care network with similar diagnostic protocols and stewardship efforts. Although the external context (such as the stay-at-home order and pandemic-related practice changes) may have influenced visit volumes, there is no indication that additional co-interventions differed systematically between the two groups. | Y |
|  | 4.4. Was the intervention implemented successfully for most participants? | The virtual urgent care service was fully integrated within the patient portal, and all visits were recorded via the EHR. The study provides clear data on visit volumes and prescribing outcomes, indicating that the service was implemented as intended. | Y |
|  | 4.5. Did study participants adhere to the assigned intervention regimen? | Patients received care in the modality (telemedicine or in-person) in which they presented, and there is no indication of cross-over or deviation from the recorded mode of visit. The EHR reliably captured the intended care delivery method. | Y |
|  | 4.6. **If N/PN to 4.3, 4.4 or 4.5**: Was an appropriate analysis used to estimate the effect of starting and adhering to the intervention? |  | NA |
|  | **Risk of bias judgement** |  | Low |
|  | Optional: What is the predicted direction of bias due to deviations from the intended interventions? |  | Unpredictable |

| **Bias due to missing data** | | | |
| --- | --- | --- | --- |
|  | 5.1 Were outcome data available for all, or nearly all, participants? | Outcome data, including antibiotic prescribing and diagnostic information, were extracted from the shared EHR across all 47 practices. The study reports a total of 8,332 ARTI visits with complete data for visit modality and antibiotic prescribing, indicating a high level of data completeness. | Y |
|  | 5.2 Were participants excluded due to missing data on intervention status? | Intervention status (VUC vs. TUC) was a routine part of the EHR record for each encounter, and there is no report of exclusions based on missing modality data. | N |
|  | 5.3 Were participants excluded due to missing data on other variables needed for the analysis? | The study does not report any substantial exclusions due to missing data on key variables such as diagnosis or antibiotic prescription. Exclusions were minimal and applied uniformly. | N |
|  | 5.4 **If PN/N to 5.1, or Y/PY to 5.2 or 5.3**: Are the proportion of participants and reasons for missing data similar across interventions? |  | NA |
|  | 5.5 **If PN/N to 5.1, or Y/PY to 5.2 or 5.3**: Is there evidence that results were robust to the presence of missing data? |  | NA |
|  | **Risk of bias judgement** |  | Low |
|  | Optional: What is the predicted direction of bias due to missing data? |  | Unpredictable |

| **Bias in measurement of outcomes** | | | |
| --- | --- | --- | --- |
|  | 6.1 Could the outcome measure have been influenced by knowledge of the intervention received? | The primary outcome—guideline-concordant antibiotic management—is based on objective EHR data (diagnosis codes and prescription records) and is not subject to subjective interpretation. Outcome measurement is therefore unlikely to be influenced by knowledge of the visit modality. | N |
|  | 6.2 Were outcome assessors aware of the intervention received by study participants? | Outcome data were extracted automatically from the shared EHR, and there is no evidence that assessors were aware of or influenced by the visit modality when recording outcomes. | N |
|  | 6.3 Were the methods of outcome assessment comparable across intervention groups? | Both telemedicine and in-person visits were evaluated using the same diagnostic criteria and EHR extraction methods. This consistency ensures that outcome assessments are comparable across the groups. | Y |
|  | 6.4 Were any systematic errors in measurement of the outcome related to intervention received? | There is no indication that the method of measuring antibiotic prescribing or guideline concordance differed between telemedicine and in-person visits. The use of standardized codes and automated extraction minimizes systematic measurement error | N |
|  | **Risk of bias judgement** |  | Low |
|  | Optional: What is the predicted direction of bias due to measurement of outcomes? |  | Unpredictable |

| **Bias in selection of the reported result** | | | |
| --- | --- | --- | --- |
|  | Is the reported effect estimate likely to be selected, on the basis of the results, from... |  |  |
|  | 7.1. ... multiple outcome *measurements* within the outcome domain? | The study pre-specified its primary outcome (guideline-concordant antibiotic management) along with secondary outcomes (diagnostic patterns and overall antibiotic prescribing trends). All these outcomes are reported comprehensively, with no indication of selective outcome reporting based on the results. | N |
|  | 7.2 ... multiple *analyses* of the intervention-outcome relationship? | The statistical analysis was performed using Fisher’s exact test and descriptive trend analyses. There is no evidence of multiple competing analyses or selective reporting of favorable results. | N |
|  | 7.3 ... different *subgroups*? | While the study examined trends over time and provided descriptive subgroup data (e.g., monthly changes in diagnoses and prescribing), these analyses were pre-specified and reported in a transparent manner without selective emphasis on particular subgroups. | N |
|  | **Risk of bias judgement** |  | Low |
|  | Optional: What is the predicted direction of bias due to selection of the reported result? |  | Unpredictable |

| **Overall bias** | | | |
| --- | --- | --- | --- |
|  | **Risk of bias judgement** | This retrospective observational study of antibiotic prescribing for sinusitis within a direct-to-consumer virtual urgent care setting uses objective EHR data and clear inclusion criteria. Outcome measures are reliably extracted and uniformly applied. However, several limitations contribute to an overall Moderate risk of bias. First, the study design is non-randomized, and the selection of a control group (a block of 100 sequential encounters from traditional urgent care) may not fully capture the variability of the TUC population. Second, provider heterogeneity between the VUC (managed by academic emergency physicians) and TUC (primarily community-based providers) introduces potential residual confounding. Finally, the relatively small sample size in the VUC group (57 patients) raises concerns regarding statistical power and the possibility of type 2 error in secondary analyses. These factors, although mitigated by the use of objective data and clear methodologies, result in an overall risk of bias judgment of Moderate | Moderate |
|  | Optional: What is the overall predicted direction of bias for this outcome? |  | Unpredictable |

# 16: Changes in antibiotic use following implementation of a telehealth stewardship pilot program [70].

|  | **Signalling questions** | **Description** | **Response options** |
| --- | --- | --- | --- |
| **Bias due to confounding** | | | |
|  | 1.1 Is there potential for confounding of the effect of intervention in this study?  **If N/PN to 1.1:** the study can be considered to be at low risk of bias due to confounding and no further signalling questions need be considered | This 1-year prospective quasi-experimental study compares antibiotic use before and after implementation of a telehealth stewardship pilot program (VAST) at two rural Veterans Affairs Medical Centers (VAMCs). The study design uses a 12-month pre-intervention period, a 1-month wash-in, and a subsequent 12-month post-intervention period. Additionally, segmented regression analysis (using ARIMA models) was employed to adjust for secular trends over time. The consistent clinical settings (acute and long-term care within the same facilities) and the objective measurement of antibiotic use (days of therapy per 1,000 days of care) help minimize confounding. Based on these design features and analytic methods, potential confounding is considered minimal. | N |
|  | **If Y/PY to 1.1**: determine whether there is a need to assess time-varying confounding: |  |  |
|  | 1.2. Was the analysis based on splitting participants’ follow up time according to intervention received?  **If N/PN**, answer questions relating to baseline confounding (1.4 to 1.6)  **If Y/PY**, go to question 1.3. |  | NA |
|  | 1.3. Were intervention discontinuations or switches likely to be related to factors that are prognostic for the outcome?  **If N/PN**, answer questions relating to baseline confounding (1.4 to 1.6)  **If Y/PY**, answer questions relating to both baseline and time-varying confounding (1.7 and 1.8) |  | NA |

|  | **Questions relating to baseline confounding only** | | |
| --- | --- | --- | --- |
|  | 1.4. Did the authors use an appropriate analysis method that controlled for all the important confounding domains? | The investigators used rate ratios, Student t tests, and segmented regression analyses to compare outcomes before and after the intervention. By incorporating a wash-in period and allowing a change in slope at the time of intervention, the analysis accounts for pre-existing trends. These methods, together with data obtained from a comprehensive electronic database, support effective control of potential confounders. | Y |
|  | 1.5. **If Y/PY to 1.4**: Were confounding domains that were controlled for measured validly and reliably by the variables available in this study? | Key variables—including antibiotic days of therapy, antibiotic spectrum index (ASI), length of therapy, and ward census data—were extracted from the VA Corporate Data Warehouse using standardized methods. The objective and consistent measurement of these variables over time ensures valid and reliable adjustment for potential confounding factors. | Y |
|  | 1.6. Did the authors control for any post-intervention variables that could have been affected by the intervention? | Intervention status was determined strictly by the calendar periods (pre- versus post-VAST) and was not influenced by outcomes. There is no evidence that the analysis adjusted for variables on the causal pathway, which minimizes the risk of introducing bias from post-intervention factors. | N |
|  | **Questions relating to baseline and time-varying confounding** | |  |
|  | 1.7. Did the authors use an appropriate analysis method that controlled for all the important confounding domains and for time-varying confounding? |  | NA |
|  | 1.8. **If Y/PY to 1.7**: Were confounding domains that were controlled for measured validly and reliably by the variables available in this study? |  | NA |
|  | **Risk of bias judgement** |  | Low |
|  | Optional: What is the predicted direction of bias due to confounding? |  | Unpredictable |

| **Bias in selection of participants into the study** | | | |
| --- | --- | --- | --- |
|  | 2.1. Was selection of participants into the study (or into the analysis) based on participant characteristics observed after the start of intervention?  **If N/PN to 2.1:** go to 2.4 | All patients receiving systemic antibiotics in the acute and long-term care units at the two VAMCs were included based on objective criteria from the electronic data warehouse. Eligibility was defined a priori, and the same criteria were applied during both the pre- and post-intervention periods, thereby minimizing selection bias. | N |
|  | 2.2. **If Y/PY to 2.1**: Were the post-intervention variables that influenced selection likely to be associated with intervention?  2.3 **If Y/PY to 2.2**: Were the post-intervention variables that influenced selection likely to be influenced by the outcome or a cause of the outcome? |  | NA  NA |
|  | 2.4. Do start of follow-up and start of intervention coincide for most participants? | For each facility, the pre-intervention and post-intervention periods are clearly demarcated by fixed dates. Outcome data were collected in alignment with these periods, ensuring that follow-up coincides appropriately with the intervention implementation. | Y |
|  | 2.5. **If Y/PY to 2.2 and 2.3, or N/PN to 2.4**: Were adjustment techniques used that are likely to correct for the presence of selection biases? |  | NA |
|  | **Risk of bias judgement** |  | Low |
|  | Optional: What is the predicted direction of bias due to selection of participants into the study? |  | Unpredictable |

| **Bias in classification of interventions** | | | |
| --- | --- | --- | --- |
|  | 3.1 Were intervention groups clearly defined? | The study defines the intervention as the implementation of a telehealth stewardship pilot program (VAST) at two specific VAMCs, with clear separation of the pre-VAST and post-VAST periods. Intervention status is based solely on the calendar period during which care was provided. | Y |
|  | 3.2 Was the information used to define intervention groups recorded at the start of the intervention? | Intervention assignment was determined prospectively by the start of the VAST at each site (with a defined wash-in period) and was documented through routine electronic data capture. This ensures that the classification of the intervention is both objective and timely. | Y |
|  | 3.3 Could classification of intervention status have been affected by knowledge of the outcome or risk of the outcome? | Classification into pre- and post-intervention groups was made solely on the basis of the date of implementation. There is no indication that outcome data influenced the classification process. | N |
|  | **Risk of bias judgement** |  | Low |
|  | Optional: What is the predicted direction of bias due to classification of interventions? |  | Unpredictable |

| **Bias due to deviations from intended interventions** | | | |
| --- | --- | --- | --- |
|  | **If your aim for this study is to assess the effect of assignment to intervention, answer questions 4.1 and 4.2** | |  |
|  | 4.1. Were there deviations from the intended intervention beyond what would be expected in usual practice? |  | Y / PY / PN / N / NI |
|  | 4.2. **If Y/PY to 4.1**: Were these deviations from intended intervention unbalanced between groups *and* likely to have affected the outcome? |  | NA / Y / PY / PN / N / NI |
|  | **If your aim for this study is to assess the effect of starting and adhering to intervention, answer questions 4.3 to 4.6** | |  |
|  | 4.3. Were important co-interventions balanced across intervention groups? | Both the acute and long-term care units at the two sites continued to operate under their usual clinical practices, with the primary change being the addition of telehealth stewardship activities. There is no evidence that other concurrent changes or co-interventions were implemented differentially between the pre- and post-intervention periods | Y |
|  | 4.4. Was the intervention implemented successfully for most participants? | The telehealth stewardship program (VAST) was implemented consistently at both sites, with clearly defined weekly telehealth sessions. Process metrics (such as changes in antibiotic days of therapy, ASI, and length of therapy) indicate that the intervention was delivered as planned. | Y |
|  | 4.5. Did study participants adhere to the assigned intervention regimen? | Patients received care in the acute and long-term care units as per standard procedures, and the VAST intervention was applied uniformly throughout the post-intervention period. The use of routinely collected electronic data to capture antibiotic use confirms that care was delivered as intended. | Y |
|  | 4.6. **If N/PN to 4.3, 4.4 or 4.5**: Was an appropriate analysis used to estimate the effect of starting and adhering to the intervention? |  | NA |
|  | **Risk of bias judgement** |  | Low |
|  | Optional: What is the predicted direction of bias due to deviations from the intended interventions? |  | Unpredictable |

| **Bias due to missing data** | | | |
| --- | --- | --- | --- |
|  | 5.1 Were outcome data available for all, or nearly all, participants? | Outcome data—such as days of therapy per 1,000 days of care, mean antibiotic spectrum index (ASI), and length of therapy—were extracted from the VA Corporate Data Warehouse and other electronic systems. The study reports complete data for the study periods, with no indication of substantial missing data. | Y |
|  | 5.2 Were participants excluded due to missing data on intervention status? | Intervention status (pre-VAST vs. post-VAST) was recorded for all patient encounters based on the defined calendar periods. There is no report of exclusions due to missing intervention data. | N |
|  | 5.3 Were participants excluded due to missing data on other variables needed for the analysis? | All key variables required for the analyses, including antibiotic use metrics and ward census data, were available from the electronic sources. Exclusions were based on predetermined criteria rather than missing data. | N |
|  | 5.4 **If PN/N to 5.1, or Y/PY to 5.2 or 5.3**: Are the proportion of participants and reasons for missing data similar across interventions? |  | NA |
|  | 5.5 **If PN/N to 5.1, or Y/PY to 5.2 or 5.3**: Is there evidence that results were robust to the presence of missing data? |  | NA |
|  | **Risk of bias judgement** |  | Low |
|  | Optional: What is the predicted direction of bias due to missing data? |  | Unpredictable |

| **Bias in measurement of outcomes** | | | |
| --- | --- | --- | --- |
|  | 6.1 Could the outcome measure have been influenced by knowledge of the intervention received? | Primary outcomes—days of therapy per 1,000 days of care, ASI, and length of therapy—are objectively measured using electronic drug administration and census data. These outcomes are not subject to subjective interpretation and are unlikely to be influenced by awareness of the intervention. | N |
|  | 6.2 Were outcome assessors aware of the intervention received by study participants? | Outcome data were extracted automatically from standardized electronic sources. There is no indication that outcome assessors were influenced by knowledge of the intervention status. | N |
|  | 6.3 Were the methods of outcome assessment comparable across intervention groups? | Both the pre-VAST and post-VAST periods employed the same methods for data collection from the VA Corporate Data Warehouse and other electronic systems. This consistency ensures that outcome measurement is comparable across the study periods. | Y |
|  | 6.4 Were any systematic errors in measurement of the outcome related to intervention received? | There is no evidence that changes in the method of care delivery (i.e., the introduction of telehealth stewardship) affected the objective measurement of antibiotic use. The use of automated and standardized data capture minimizes the risk of systematic error. | N |
|  | **Risk of bias judgement** |  | Low |
|  | Optional: What is the predicted direction of bias due to measurement of outcomes? |  | Unpredictable |

| **Bias in selection of the reported result** | | | |
| --- | --- | --- | --- |
|  | Is the reported effect estimate likely to be selected, on the basis of the results, from... |  |  |
|  | 7.1. ... multiple outcome *measurements* within the outcome domain? | The study pre-specified its primary outcome (antibiotic days of therapy per 1,000 days of care) along with secondary outcomes (mean ASI and mean length of therapy). All outcomes were reported comprehensively, and there is no indication of selective reporting. | N |
|  | 7.2 ... multiple *analyses* of the intervention-outcome relationship? | The statistical analysis plan included both rate ratio comparisons and segmented regression analyses to assess trends over time. There is no evidence that multiple competing analyses were performed with selective emphasis on favorable outcomes. | N |
|  | 7.3 ... different *subgroups*? | Subgroup analyses (for example, analyses by antibiotic class or by setting—acute versus long-term care) were pre-specified and reported transparently. There is no indication that results were selectively chosen from among multiple subgroup analyses. | N |
|  | **Risk of bias judgement** |  | Low |
|  | Optional: What is the predicted direction of bias due to selection of the reported result? |  | Unpredictable |

| **Overall bias** | | | |
| --- | --- | --- | --- |
|  | **Risk of bias judgement** | This prospective quasi-experimental study was conducted at two rural VAMCs and employed objective, routinely collected data to evaluate the impact of a telehealth stewardship pilot program on antibiotic use. The study used robust analytic methods—including segmented regression with ARIMA modeling—to adjust for secular trends over a 12-month pre-intervention and 12-month post-intervention period (after a wash-in phase). The outcomes (days of therapy, ASI, and length of therapy) were measured consistently using standardized electronic sources. Although the quasi-experimental design introduces some inherent limitations compared with randomized trials, the comprehensive data collection, objective outcome measurement, and appropriate analytic adjustments support an overall risk of bias judgment of Low | Low |
|  | Optional: What is the overall predicted direction of bias for this outcome? |  | Unpredictable |

# 17: Implementation of telehealth antimicrobial stewardship through partnership of an academic medical center and a community hospital [71].

|  | **Signalling questions** | **Description** | **Response options** |
| --- | --- | --- | --- |
| **Bias due to confounding** | | | |
|  | 1.1 Is there potential for confounding of the effect of intervention in this study?  **If N/PN to 1.1:** the study can be considered to be at low risk of bias due to confounding and no further signalling questions need be considered | This quasi-experimental study compared antimicrobial stewardship outcomes across three distinct periods—baseline (pre-intervention), intervention (telehealth stewardship provided by an academic medical center), and post-intervention (after withdrawal of telehealth services)—at a single community hospital. Patient selection was based on objective ICD diagnosis codes for pneumonia, skin and soft tissue infections, and urinary tract infections, and baseline patient characteristics (including demographic data and Charlson Comorbidity Index scores) were reported as similar across study phases. In addition, a 5‑month washout period was incorporated between phases to minimize carryover effects. These design features, along with uniform data collection, minimize the risk of confounding, and there is no evidence that any extraneous variables differentially influenced the outcomes across periods. | N |
|  | **If Y/PY to 1.1**: determine whether there is a need to assess time-varying confounding: |  |  |
|  | 1.2. Was the analysis based on splitting participants’ follow up time according to intervention received?  **If N/PN**, answer questions relating to baseline confounding (1.4 to 1.6)  **If Y/PY**, go to question 1.3. |  | NA |
|  | 1.3. Were intervention discontinuations or switches likely to be related to factors that are prognostic for the outcome?  **If N/PN**, answer questions relating to baseline confounding (1.4 to 1.6)  **If Y/PY**, answer questions relating to both baseline and time-varying confounding (1.7 and 1.8) |  | NA |

|  | **Questions relating to baseline confounding only** | | |
| --- | --- | --- | --- |
|  | 1.4. Did the authors use an appropriate analysis method that controlled for all the important confounding domains? | The investigators conducted comparative analyses using rate ratios, Student t tests, and segmented regression (with a change in slope at the wash-in period) to evaluate differences in primary outcomes (e.g., days of therapy per 1,000 patient-days) and secondary outcomes (e.g., antibiotic spectrum index and length of therapy). These analytic methods account for secular trends and baseline differences over time, supporting effective control for potential confounding factors. | Y |
|  | 1.5. **If Y/PY to 1.4**: Were confounding domains that were controlled for measured validly and reliably by the variables available in this study? | Key outcome measures—such as antibiotic days of therapy, antibiotic spectrum index, and length of therapy—were obtained from the VA Corporate Data Warehouse and related electronic systems using standardized methods. Patient-level variables (e.g., diagnoses, Charlson Comorbidity Index scores) were also collected consistently across all study phases. These objective and reliable data sources ensure that the confounding domains were measured validly. | Y |
|  | 1.6. Did the authors control for any post-intervention variables that could have been affected by the intervention? | Intervention status was determined exclusively by calendar period (pre-, during, and post-telehealth stewardship) with a predefined washout period. Outcome measurement was based on routinely collected data, and no adjustments were made for variables that lie on the causal pathway between the intervention and outcomes. | N |
|  | **Questions relating to baseline and time-varying confounding** | |  |
|  | 1.7. Did the authors use an appropriate analysis method that controlled for all the important confounding domains and for time-varying confounding? |  | NA |
|  | 1.8. **If Y/PY to 1.7**: Were confounding domains that were controlled for measured validly and reliably by the variables available in this study? |  | NA |
|  | **Risk of bias judgement** |  | Low |
|  | Optional: What is the predicted direction of bias due to confounding? |  | Unpredictable |

| **Bias in selection of participants into the study** | | | |
| --- | --- | --- | --- |
|  | 2.1. Was selection of participants into the study (or into the analysis) based on participant characteristics observed after the start of intervention?  **If N/PN to 2.1:** go to 2.4 | The study included all patients admitted to the community hospital with select infectious disease diagnoses (pneumonia, skin and soft tissue infections, and urinary tract infections) as identified by ICD codes at the time of discharge. Inclusion and exclusion criteria were defined a priori and applied uniformly across the baseline, intervention, and post-intervention periods. There is no evidence that participants were selected based on characteristics observed after the intervention started. | N |
|  | 2.2. **If Y/PY to 2.1**: Were the post-intervention variables that influenced selection likely to be associated with intervention?  2.3 **If Y/PY to 2.2**: Were the post-intervention variables that influenced selection likely to be influenced by the outcome or a cause of the outcome? |  | NA  NA |
|  | 2.4. Do start of follow-up and start of intervention coincide for most participants? | For each patient, the index event (hospital admission with one of the target diagnoses) was used to define the follow-up period, and the timing of care was classified according to the predetermined study phases. This ensures that outcome data are appropriately aligned with the corresponding intervention period. | Y |
|  | 2.5. **If Y/PY to 2.2 and 2.3, or N/PN to 2.4**: Were adjustment techniques used that are likely to correct for the presence of selection biases? |  | NA |
|  | **Risk of bias judgement** |  | Low |
|  | Optional: What is the predicted direction of bias due to selection of participants into the study? |  | Unpredictable |

| **Bias in classification of interventions** | | | |
| --- | --- | --- | --- |
|  | 3.1 Were intervention groups clearly defined? | The study clearly delineates the intervention by defining three phases: a baseline period (pre-telehealth stewardship), an intervention period (when telehealth stewardship services were provided by an academic medical center), and a post-intervention period (after the telehealth services were withdrawn). This time-based classification is explicit and unambiguous. | Y |
|  | 3.2 Was the information used to define intervention groups recorded at the start of the intervention? | Intervention status was assigned based on calendar dates and documented through routine clinical records. The use of the same electronic medical record (EMR) system throughout the study ensures that assignment to each period was determined prospectively and objectively. | Y |
|  | 3.3 Could classification of intervention status have been affected by knowledge of the outcome or risk of the outcome? | The classification into pre-, during-, and post-intervention periods was based solely on the timing of the telehealth stewardship service implementation and withdrawal. There is no indication that outcome data influenced the categorization of intervention status | N |
|  | **Risk of bias judgement** |  | Low |
|  | Optional: What is the predicted direction of bias due to classification of interventions? |  | Unpredictable |

| **Bias due to deviations from intended interventions** | | | |
| --- | --- | --- | --- |
|  | **If your aim for this study is to assess the effect of assignment to intervention, answer questions 4.1 and 4.2** | |  |
|  | 4.1. Were there deviations from the intended intervention beyond what would be expected in usual practice? |  | Y / PY / PN / N / NI |
|  | 4.2. **If Y/PY to 4.1**: Were these deviations from intended intervention unbalanced between groups *and* likely to have affected the outcome? |  | NA / Y / PY / PN / N / NI |
|  | **If your aim for this study is to assess the effect of starting and adhering to intervention, answer questions 4.3 to 4.6** | |  |
|  | 4.3. Were important co-interventions balanced across intervention groups? | Both the baseline and post-intervention periods reflect the community hospital’s usual antimicrobial stewardship practices, while the intervention period introduced telehealth-supported stewardship services provided remotely by an academic medical center. Aside from the telehealth component, no additional co-interventions were reported, and all phases maintained similar standard care protocols for the target conditions | Y |
|  | 4.4. Was the intervention implemented successfully for most participants? | The telehealth stewardship intervention was implemented for a defined 7‑month period, with services provided consistently via remote sessions, prospective audit and feedback, guideline updates, and educational sessions. Process metrics (e.g., the rate of telehealth interventions and acceptance by community hospital pharmacists) were documented, indicating that the intervention was delivered as intended. | Y |
|  | 4.5. Did study participants adhere to the assigned intervention regimen? | Patients received care according to the community hospital’s standard practices during the baseline and post-intervention periods, while during the intervention period, telehealth stewardship services were integrated into the care process. There is no indication that patients crossed over between phases, and the adherence to the intervention is reflected by consistent data collection via the usual ASP workflow | Y |
|  | 4.6. **If N/PN to 4.3, 4.4 or 4.5**: Was an appropriate analysis used to estimate the effect of starting and adhering to the intervention? |  | NA |
|  | **Risk of bias judgement** |  | Low |
|  | Optional: What is the predicted direction of bias due to deviations from the intended interventions? |  | Unpredictable |

| **Bias due to missing data** | | | |
| --- | --- | --- | --- |
|  | 5.1 Were outcome data available for all, or nearly all, participants? | Outcome data on antibiotic utilization (days of therapy per 1,000 patient-days), antibiotic spectrum index, and length of therapy were extracted retrospectively from the electronic medical records and the VA Corporate Data Warehouse. The study reports complete data for the periods evaluated, with clearly defined exclusion criteria that were applied uniformly. There is no indication of substantial missing data. | Y |
|  | 5.2 Were participants excluded due to missing data on intervention status? | Intervention status was defined by the calendar period in which patients were admitted. There is no evidence that any patient was excluded because of missing data regarding the intervention period. | N |
|  | 5.3 Were participants excluded due to missing data on other variables needed for the analysis? | All necessary variables (demographics, diagnosis, antibiotic use metrics, and clinical outcomes) were available from the standard ASP workflow and electronic sources. Exclusions were limited to predetermined criteria (e.g., transfers, early deaths) rather than missing data | N |
|  | 5.4 **If PN/N to 5.1, or Y/PY to 5.2 or 5.3**: Are the proportion of participants and reasons for missing data similar across interventions? |  | NA |
|  | 5.5 **If PN/N to 5.1, or Y/PY to 5.2 or 5.3**: Is there evidence that results were robust to the presence of missing data? |  | NA |
|  | **Risk of bias judgement** |  | Low |
|  | Optional: What is the predicted direction of bias due to missing data? |  | Unpredictable |

| **Bias in measurement of outcomes** | | | |
| --- | --- | --- | --- |
|  | 6.1 Could the outcome measure have been influenced by knowledge of the intervention received? | Primary outcomes—including days of therapy per 1,000 patient-days, the mean antibiotic spectrum index, and mean length of therapy—are objectively measured using standardized data from the VA Corporate Data Warehouse and EMR. These measures are not subject to subjective interpretation and are unlikely to be influenced by knowledge of the intervention. | N |
|  | 6.2 Were outcome assessors aware of the intervention received by study participants? | Outcome data were extracted via routine electronic systems as part of the usual ASP workflow, and there is no indication that the assessors were aware of or influenced by the intervention status during data collection. | N |
|  | 6.3 Were the methods of outcome assessment comparable across intervention groups? | The same electronic data extraction methods and definitions were applied across the baseline, intervention, and post-intervention periods, ensuring that outcomes are measured consistently across all phases." | Y |
|  | 6.4 Were any systematic errors in measurement of the outcome related to intervention received? | There is no evidence that the method of measuring antibiotic use or clinical outcomes changed between the study periods. The objective nature of the data and the standardized extraction process minimize the risk of systematic measurement error. | N |
|  | **Risk of bias judgement** |  | Low |
|  | Optional: What is the predicted direction of bias due to measurement of outcomes? |  | Unpredictable |

| **Bias in selection of the reported result** | | | |
| --- | --- | --- | --- |
|  | Is the reported effect estimate likely to be selected, on the basis of the results, from... |  |  |
|  | 7.1. ... multiple outcome *measurements* within the outcome domain? | The study pre-specified its primary outcome (utilization of select broad-spectrum antibiotics measured as DOT per 1,000 patient-days) and secondary outcomes (antibiotic spectrum index, length of therapy, length of stay, readmission, 30-day mortality, and clinician satisfaction). These outcomes were reported comprehensively, and there is no evidence of selective reporting based on the results. | N |
|  | 7.2 ... multiple *analyses* of the intervention-outcome relationship? | Statistical analyses were conducted using standard methods (Fisher’s exact test, unpaired t tests, and segmented regression models) as pre-specified in the protocol. There is no indication that multiple alternative analyses were performed and selectively reported. | N |
|  | 7.3 ... different *subgroups*? | While the study reports subgroup data for different care settings (acute and long-term) and for individual antibiotic classes, these subgroup analyses were pre-specified and presented transparently without selective emphasis on favorable results. | N |
|  | **Risk of bias judgement** |  | Low |
|  | Optional: What is the predicted direction of bias due to selection of the reported result? |  | Unpredictable |

| **Overall bias** | | | |
| --- | --- | --- | --- |
|  | **Risk of bias judgement** | This prospective quasi-experimental study, conducted over three phases (baseline, intervention, and post-intervention), was designed to evaluate the impact of a telehealth antimicrobial stewardship pilot program implemented via a partnership between an academic medical center and a community hospital. The study employed robust design features—including equivalent time periods, a washout period, and standardized data collection from electronic sources—to ensure that baseline characteristics and outcomes were measured consistently. Objective outcome measures (DOT per 1,000 patient-days, ASI, and length of therapy) were analyzed using appropriate statistical methods, and potential confounding factors were minimized by the stable clinical setting and uniform inclusion criteria. Although quasi-experimental designs have inherent limitations compared with randomized trials, the comprehensive and objective nature of the data, along with the careful analytical approach, supports an overall risk of bias judgment of Low. | Low |
|  | Optional: What is the overall predicted direction of bias for this outcome? |  | Unpredictable |

# 18: Effect of adding a mobile health intervention to a multimodal antimicrobial stewardship programme across three teaching hospitals: an interrupted time series study [72].

|  | **Signalling questions** | **Description** | **Response options** |
| --- | --- | --- | --- |
| **Bias due to confounding** | | | |
|  | 1.1 Is there potential for confounding of the effect of intervention in this study?  **If N/PN to 1.1:** the study can be considered to be at low risk of bias due to confounding and no further signalling questions need be considered | This quasi-experimental interrupted time series (ITS) study examined changes in proxy antibiotic prescribing indicators before and after the introduction of a mobile health intervention (IAPP) as an adjunct to an existing multimodal antimicrobial stewardship programme. Although the analysis employed segmented regression with ARIMA models to account for underlying trends and autocorrelation, the ITS design remains inherently susceptible to confounding by secular trends and other concurrent changes (e.g., updates to local guidelines or other stewardship initiatives). Thus, despite robust statistical methods, residual confounding is plausible. | PY |
|  | **If Y/PY to 1.1**: determine whether there is a need to assess time-varying confounding: |  |  |
|  | 1.2. Was the analysis based on splitting participants’ follow up time according to intervention received?  **If N/PN**, answer questions relating to baseline confounding (1.4 to 1.6)  **If Y/PY**, go to question 1.3. |  | NA |
|  | 1.3. Were intervention discontinuations or switches likely to be related to factors that are prognostic for the outcome?  **If N/PN**, answer questions relating to baseline confounding (1.4 to 1.6)  **If Y/PY**, answer questions relating to both baseline and time-varying confounding (1.7 and 1.8) |  | NA |

|  | **Questions relating to baseline confounding only** | | |
| --- | --- | --- | --- |
|  | 1.4. Did the authors use an appropriate analysis method that controlled for all the important confounding domains? | The study used a segmented regression analysis of six-monthly point prevalence survey (PPS) data spanning from October 2008 to June 2014. With the intervention period clearly defined as August 2011 and with at least three data points before and after the intervention, the ITS design enabled estimation of immediate level changes and slope changes. The use of ARIMA models (after confirming the absence of first-order autocorrelation via the Durbin–Watson statistic) is appropriate; however, given the complex and multifaceted nature of the antimicrobial stewardship interventions, some time-varying confounders may remain unaccounted for. | Y |
|  | 1.5. **If Y/PY to 1.4**: Were confounding domains that were controlled for measured validly and reliably by the variables available in this study? | The key outcome measures—compliance with antimicrobial policy, documentation of indication, and documentation of stop/review dates—were derived from routinely collected data using standardized, protocol-driven forms completed by clinical pharmacists. These objective measures are valid and reliably captured. Nonetheless, while these data accurately reflect prescribing practices, the ITS design cannot fully eliminate potential confounding from unmeasured contextual changes over the lengthy study period. | Y |
|  | 1.6. Did the authors control for any post-intervention variables that could have been affected by the intervention? | Intervention status was defined solely by calendar period (pre- versus post-IAPP adoption), and outcome measurement was based on routinely collected data from the PPS. There is no evidence that variables measured after the intervention (or that lie on the causal pathway) were inappropriately adjusted for | N |
|  | **Questions relating to baseline and time-varying confounding** | |  |
|  | 1.7. Did the authors use an appropriate analysis method that controlled for all the important confounding domains and for time-varying confounding? |  | NA |
|  | 1.8. **If Y/PY to 1.7**: Were confounding domains that were controlled for measured validly and reliably by the variables available in this study? |  | NA |
|  | **Risk of bias judgement** |  | Moderate |
|  | Optional: What is the predicted direction of bias due to confounding? |  | Unpredictable |

| **Bias in selection of participants into the study** | | | |
| --- | --- | --- | --- |
|  | 2.1. Was selection of participants into the study (or into the analysis) based on participant characteristics observed after the start of intervention?  **If N/PN to 2.1:** go to 2.4 | All patients receiving systemic antimicrobials in the participating teaching hospitals were included in the periodic point prevalence surveys (PPS). Inclusion was determined using objective criteria (e.g., ICD diagnosis codes and medication chart reviews) that were applied uniformly across the entire study period. There is no evidence that participant selection was influenced by outcomes or post-intervention characteristics. | N |
|  | 2.2. **If Y/PY to 2.1**: Were the post-intervention variables that influenced selection likely to be associated with intervention?  2.3 **If Y/PY to 2.2**: Were the post-intervention variables that influenced selection likely to be influenced by the outcome or a cause of the outcome? |  | NA  NA |
|  | 2.4. Do start of follow-up and start of intervention coincide for most participants? | Data were collected at fixed, six-monthly intervals spanning the pre-intervention, intervention, and post-intervention periods. This consistent timing ensures that follow-up aligns appropriately with the defined intervention periods. | Y |
|  | 2.5. **If Y/PY to 2.2 and 2.3, or N/PN to 2.4**: Were adjustment techniques used that are likely to correct for the presence of selection biases? |  | NA |
|  | **Risk of bias judgement** |  | Low |
|  | Optional: What is the predicted direction of bias due to selection of participants into the study? |  | Unpredictable |

| **Bias in classification of interventions** | | | |
| --- | --- | --- | --- |
|  | 3.1 Were intervention groups clearly defined? | The intervention is clearly defined by the time period in which the mobile health component (IAPP) was added to the existing antimicrobial stewardship programme. The study design delineates pre-intervention and post-intervention periods with a defined wash-in period, allowing for clear classification of data according to the intervention status. | Y |
|  | 3.2 Was the information used to define intervention groups recorded at the start of the intervention? | Intervention status was assigned based on predetermined calendar dates and documented through routinely collected electronic data. This ensures that patients were classified according to the care model in place at the time of their antimicrobial use. | Y |
|  | 3.3 Could classification of intervention status have been affected by knowledge of the outcome or risk of the outcome? | Classification was solely based on the timing of data collection relative to the intervention, independent of outcome data. Thus, outcome knowledge did not influence intervention group assignment. | N |
|  | **Risk of bias judgement** |  | Low |
|  | Optional: What is the predicted direction of bias due to classification of interventions? |  | Unpredictable |

| **Bias due to deviations from intended interventions** | | | |
| --- | --- | --- | --- |
|  | **If your aim for this study is to assess the effect of assignment to intervention, answer questions 4.1 and 4.2** | |  |
|  | 4.1. Were there deviations from the intended intervention beyond what would be expected in usual practice? |  | Y / PY / PN / N / NI |
|  | 4.2. **If Y/PY to 4.1**: Were these deviations from intended intervention unbalanced between groups *and* likely to have affected the outcome? |  | NA / Y / PY / PN / N / NI |
|  | **If your aim for this study is to assess the effect of starting and adhering to intervention, answer questions 4.3 to 4.6** | |  |
|  | 4.3. Were important co-interventions balanced across intervention groups? | All participating teaching hospitals maintained their standard antimicrobial stewardship programmes throughout the study. The addition of the mobile health intervention was the primary change during the intervention period. There is no evidence that other co-interventions changed differentially across the study periods. | Y |
|  | 4.4. Was the intervention implemented successfully for most participants? | The mobile health intervention was implemented as part of the overall stewardship programme, and data were collected prospectively using a standardized protocol. The segmented regression analysis indicates that the intervention had measurable effects on the proxy prescribing indicators, suggesting successful implementation. | Y |
|  | 4.5. Did study participants adhere to the assigned intervention regimen? | Patients received antimicrobial prescriptions as part of routine care, and the intervention (mobile health component) was applied at the organizational level. There is no indication that patients deviated from the standard care pathways. Adherence to the intended intervention is assumed from the consistent application of the mobile app–supported data collection and audit procedures. | Y |
|  | 4.6. **If N/PN to 4.3, 4.4 or 4.5**: Was an appropriate analysis used to estimate the effect of starting and adhering to the intervention? |  | NA |
|  | **Risk of bias judgement** |  | Low |
|  | Optional: What is the predicted direction of bias due to deviations from the intended interventions? |  | Unpredictable |

| **Bias due to missing data** | | | |
| --- | --- | --- | --- |
|  | 5.1 Were outcome data available for all, or nearly all, participants? | Outcome data on the three key prescribing indicators (choice, indication, and stop/review date) were collected using a standardized, protocol-driven form by clinical pharmacists across multiple time points over nearly six years. Data appear to be comprehensive, with no indication of substantial missing data affecting the analysis. | Y |
|  | 5.2 Were participants excluded due to missing data on intervention status? | Intervention classification was based on calendar dates and was captured in the routinely collected dataset. There is no report of exclusions based on missing intervention status. | N |
|  | 5.3 Were participants excluded due to missing data on other variables needed for the analysis? | All necessary variables for analysis (demographics, antimicrobial prescribing indicators) were available from the electronic data sources. Exclusions, if any, were due to pre-specified criteria rather than missing data. | N |
|  | 5.4 **If PN/N to 5.1, or Y/PY to 5.2 or 5.3**: Are the proportion of participants and reasons for missing data similar across interventions? |  | NA |
|  | 5.5 **If PN/N to 5.1, or Y/PY to 5.2 or 5.3**: Is there evidence that results were robust to the presence of missing data? |  | NA |
|  | **Risk of bias judgement** |  | Low |
|  | Optional: What is the predicted direction of bias due to missing data? |  | Unpredictable |

| **Bias in measurement of outcomes** | | | |
| --- | --- | --- | --- |
|  | 6.1 Could the outcome measure have been influenced by knowledge of the intervention received? | Primary outcomes (compliance with antimicrobial policy, documentation of indication, and documentation of stop/review date) were derived from objective, routinely collected data using a standardized data collection form. These outcomes are based on chart documentation and are unlikely to be influenced by knowledge of the intervention. | N |
|  | 6.2 Were outcome assessors aware of the intervention received by study participants? | Data collection was performed by clinical pharmacists using a standardized form, and outcome assessment was conducted as part of routine stewardship monitoring. There is no indication that assessors were influenced by knowledge of the intervention status. | N |
|  | 6.3 Were the methods of outcome assessment comparable across intervention groups? | The same data collection procedures and definitions were used consistently throughout the pre-intervention and post-intervention periods across all hospitals. This uniform approach ensures comparability of outcome measurements. | Y |
|  | 6.4 Were any systematic errors in measurement of the outcome related to intervention received? | There is no evidence that the mobile health intervention affected the method by which outcomes were measured. The use of objective chart data and standardized forms minimizes the risk of systematic measurement error | N |
|  | **Risk of bias judgement** |  | Low |
|  | Optional: What is the predicted direction of bias due to measurement of outcomes? |  | Unpredictable |

| **Bias in selection of the reported result** | | | |
| --- | --- | --- | --- |
|  | Is the reported effect estimate likely to be selected, on the basis of the results, from... |  |  |
|  | 7.1. ... multiple outcome *measurements* within the outcome domain? | The study pre-specified the three primary proxy indicators of antibiotic prescribing and reported the segmented regression analyses for each outcome. There is no indication that the authors selectively reported outcomes from among multiple measurements. | N |
|  | 7.2 ... multiple *analyses* of the intervention-outcome relationship? | The statistical analysis plan, including segmented regression with ARIMA modeling, was clearly outlined and applied uniformly to all outcomes. There is no evidence that multiple competing analyses were performed with selective reporting of results. | N |
|  | 7.3 ... different *subgroups*? | Although the study presents data for medical and surgical specialties separately, these subgroup analyses were pre-specified as part of the overall evaluation framework. There is no indication that results were selectively reported from among various subgroups. | N |
|  | **Risk of bias judgement** |  | Low |
|  | Optional: What is the predicted direction of bias due to selection of the reported result? |  | Unpredictable |

| **Overall bias** | | | |
| --- | --- | --- | --- |
|  | **Risk of bias judgement** | This prospective quasi-experimental study evaluated the impact of adding a mobile health intervention to an existing multimodal antimicrobial stewardship programme across three teaching hospitals using an interrupted time series design. With clearly defined pre-intervention and post-intervention periods, a wash-in phase, and standardized, routinely collected outcome measures (via a protocol-driven chart review by clinical pharmacists), the study minimized potential biases in data collection and outcome measurement. Although the ITS design carries inherent risks of residual confounding, the robust analytic methods (segmented regression with ARIMA) and consistent measurement of proxy prescribing indicators support an overall risk of bias judgment of Low | Moderate |
|  | Optional: What is the overall predicted direction of bias for this outcome? |  | Unpredictable |

# 19: Sustained impact of a computer-assisted antimicrobial stewardship intervention on antimicrobial use and length of stay [73].

|  | **Signalling questions** | **Description** | **Response options** |
| --- | --- | --- | --- |
| **Bias due to confounding** | | | |
|  | 1.1 Is there potential for confounding of the effect of intervention in this study?  **If N/PN to 1.1:** the study can be considered to be at low risk of bias due to confounding and no further signalling questions need be considered | The study is a retrospective cohort study comparing outcomes before and after the implementation of a computer-assisted antimicrobial stewardship intervention (APSS-initiated PAF strategy) at a large teaching hospital. The intervention was implemented on 18 August 2010, with data collected for the pre-intervention period (August 2008–July 2010) and the intervention period (August 2010–July 2013). Although the authors used segmented regression analysis (with ARIMA modeling) to adjust for time trends and seasonality, the nonrandomized design over a five-year period leaves room for residual confounding due to unmeasured changes in clinical practice, introduction of generic antimicrobials, and other secular trends. | PY |
|  | **If Y/PY to 1.1**: determine whether there is a need to assess time-varying confounding: |  |  |
|  | 1.2. Was the analysis based on splitting participants’ follow up time according to intervention received?  **If N/PN**, answer questions relating to baseline confounding (1.4 to 1.6)  **If Y/PY**, go to question 1.3. |  | NA |
|  | 1.3. Were intervention discontinuations or switches likely to be related to factors that are prognostic for the outcome?  **If N/PN**, answer questions relating to baseline confounding (1.4 to 1.6)  **If Y/PY**, answer questions relating to both baseline and time-varying confounding (1.7 and 1.8) |  | NA |

|  | **Questions relating to baseline confounding only** | | |
| --- | --- | --- | --- |
|  | 1.4. Did the authors use an appropriate analysis method that controlled for all the important confounding domains? | The study employed segmented regression analysis of an interrupted time series, using 65 four-week periods (26 pre-intervention and 39 intervention) and ARIMA modeling to adjust for time trends, autocorrelation, and seasonal effects. This approach is appropriate for controlling many confounding factors inherent in a longitudinal design; however, despite these adjustments, some residual confounding remains due to the absence of randomization. | Y |
|  | 1.5. **If Y/PY to 1.4**: Were confounding domains that were controlled for measured validly and reliably by the variables available in this study? | Key outcome variables (such as days of therapy per 1,000 patient-days, DDD/1000 PDs, antimicrobial spending, and average length of stay) were extracted from the hospital’s clinical data warehouse and pharmacy financial databases using standardized methods. These objective measurements are valid and reliable. Nonetheless, while many potential confounders (e.g., basic demographic characteristics) were accounted for, unmeasured factors related to evolving clinical practices may still influence the outcomes. | Y |
|  | 1.6. Did the authors control for any post-intervention variables that could have been affected by the intervention? | Intervention status was defined strictly by the calendar period (pre-intervention vs. intervention) with a defined washout phase, and outcome data were collected independently using routine electronic systems. There is no indication that variables measured after the intervention (or on the causal pathway) were inappropriately adjusted for | N |
|  | **Questions relating to baseline and time-varying confounding** | |  |
|  | 1.7. Did the authors use an appropriate analysis method that controlled for all the important confounding domains and for time-varying confounding? |  | NA |
|  | 1.8. **If Y/PY to 1.7**: Were confounding domains that were controlled for measured validly and reliably by the variables available in this study? |  | NA |
|  | **Risk of bias judgement** |  | Moderate |
|  | Optional: What is the predicted direction of bias due to confounding? |  | Unpredictable |

| **Bias in selection of participants into the study** | | | |
| --- | --- | --- | --- |
|  | 2.1. Was selection of participants into the study (or into the analysis) based on participant characteristics observed after the start of intervention?  **If N/PN to 2.1:** go to 2.4 | The study included all hospitalized adult patients (≥18 years) who received intravenous or oral antimicrobials at the Centre Hospitalier Universitaire de Sherbrooke during the defined study period, with exclusions applied uniformly (psychiatric and maternity units). Participant selection was based on objective criteria captured prospectively in the clinical data warehouse, without influence from post-intervention outcomes. | N |
|  | 2.2. **If Y/PY to 2.1**: Were the post-intervention variables that influenced selection likely to be associated with intervention?  2.3 **If Y/PY to 2.2**: Were the post-intervention variables that influenced selection likely to be influenced by the outcome or a cause of the outcome? |  | NA  NA |
|  | 2.4. Do start of follow-up and start of intervention coincide for most participants? | For each patient, the admission date defined the start of follow-up, and the assignment to pre-intervention or intervention groups was determined by fixed calendar dates. This ensures that follow-up timing is consistent with the intervention status. | Y |
|  | 2.5. **If Y/PY to 2.2 and 2.3, or N/PN to 2.4**: Were adjustment techniques used that are likely to correct for the presence of selection biases? |  | NA |
|  | **Risk of bias judgement** |  | Low |
|  | Optional: What is the predicted direction of bias due to selection of participants into the study? |  | Unpredictable |

| **Bias in classification of interventions** | | | |
| --- | --- | --- | --- |
|  | 3.1 Were intervention groups clearly defined? | Intervention groups were defined based on the time period relative to the implementation of the computer-assisted stewardship intervention. The pre-intervention phase (August 2008–July 2010) and the intervention phase (August 2010–July 2013) are clearly delineated, with the intervention beginning on 18 August 2010. | Y |
|  | 3.2 Was the information used to define intervention groups recorded at the start of the intervention? | The assignment to pre-intervention or intervention groups was determined by admission dates recorded in the hospital’s clinical data warehouse. This information was captured prospectively using standardized electronic records, ensuring objective classification. | Y |
|  | 3.3 Could classification of intervention status have been affected by knowledge of the outcome or risk of the outcome? | Classification into the pre-intervention and intervention groups was solely based on calendar dates and the implementation of the stewardship intervention. There is no indication that outcome data influenced this classification. | N |
|  | **Risk of bias judgement** |  | Low |
|  | Optional: What is the predicted direction of bias due to classification of interventions? |  | Unpredictable |

| **Bias due to deviations from intended interventions** | | | |
| --- | --- | --- | --- |
|  | **If your aim for this study is to assess the effect of assignment to intervention, answer questions 4.1 and 4.2** | |  |
|  | 4.1. Were there deviations from the intended intervention beyond what would be expected in usual practice? |  | Y / PY / PN / N / NI |
|  | 4.2. **If Y/PY to 4.1**: Were these deviations from intended intervention unbalanced between groups *and* likely to have affected the outcome? |  | NA / Y / PY / PN / N / NI |
|  | **If your aim for this study is to assess the effect of starting and adhering to intervention, answer questions 4.3 to 4.6** | |  |
|  | 4.3. Were important co-interventions balanced across intervention groups? | Both study phases occurred within the same hospital environment, and the only systematic change was the implementation of the computer-assisted ASP (APSS-initiated PAF strategy). Although there were known co-interventions (e.g., introduction of generic antimicrobials) that were explicitly modeled, no other major changes in clinical practice were reported that differentially affected the groups. | Y |
|  | 4.4. Was the intervention implemented successfully for most participants? | The intervention was implemented as planned, with an initial allocation of a 0.5 full-time equivalent clinical pharmacist that increased to one full-time pharmacist, supported by an infectious diseases physician. Process measures (e.g., the number and type of stewardship recommendations) and significant changes in key outcomes (e.g., reductions in LOS and antimicrobial consumption) indicate that the intervention was successfully delivered. | Y |
|  | 4.5. Did study participants adhere to the assigned intervention regimen? | Patient care was delivered according to standard hospital protocols, and the computer-assisted ASP was applied consistently during the intervention phase. There is no evidence of deviations from the assigned care model, and outcomes were captured as part of routine clinical practice. | Y |
|  | 4.6. **If N/PN to 4.3, 4.4 or 4.5**: Was an appropriate analysis used to estimate the effect of starting and adhering to the intervention? |  | NA |
|  | **Risk of bias judgement** |  | Low |
|  | Optional: What is the predicted direction of bias due to deviations from the intended interventions? |  | Unpredictable |

| **Bias due to missing data** | | | |
| --- | --- | --- | --- |
|  | 5.1 Were outcome data available for all, or nearly all, participants? | Outcome data for antimicrobial consumption (DOT and DDD per 1,000 patient-days), antimicrobial spending, length of stay, and guideline non-concordance were extracted from the CHUS clinical data warehouse and pharmacy financial databases for 40,605 hospitalization episodes. The dataset is comprehensive, and no substantial missing data issues were reported. | Y |
|  | 5.2 Were participants excluded due to missing data on intervention status? | Intervention status was defined by admission dates, which were available for all patients. There is no indication that any patients were excluded due to missing information on intervention status. | N |
|  | 5.3 Were participants excluded due to missing data on other variables needed for the analysis? | All necessary variables (demographic, clinical, and antimicrobial use data) were obtained from routine electronic sources. Exclusions were based on pre-specified criteria rather than missing data. | N |
|  | 5.4 **If PN/N to 5.1, or Y/PY to 5.2 or 5.3**: Are the proportion of participants and reasons for missing data similar across interventions? |  | NA |
|  | 5.5 **If PN/N to 5.1, or Y/PY to 5.2 or 5.3**: Is there evidence that results were robust to the presence of missing data? |  | NA |
|  | **Risk of bias judgement** |  | Low |
|  | Optional: What is the predicted direction of bias due to missing data? |  | Unpredictable |

| **Bias in measurement of outcomes** | | | |
| --- | --- | --- | --- |
|  | 6.1 Could the outcome measure have been influenced by knowledge of the intervention received? | The primary outcomes (e.g., DOT per 1,000 patient-days, DDD per 1,000 patient-days, antimicrobial spending, LOS, and non-concordance with prescribing guidelines) are objectively measured using standardized electronic data from the clinical data warehouse and financial databases. These measures are not subject to subjective interpretation. | N |
|  | 6.2 Were outcome assessors aware of the intervention received by study participants? | Outcome data were extracted automatically using routine electronic methods, and there is no indication that assessors were influenced by knowledge of the intervention status | N |
|  | 6.3 Were the methods of outcome assessment comparable across intervention groups? | The same data extraction methods and definitions were used across both the pre-intervention and intervention periods. This uniformity ensures that outcome assessment is directly comparable between groups." | Y |
|  | 6.4 Were any systematic errors in measurement of the outcome related to intervention received? | There is no evidence that the method of outcome measurement changed as a result of the intervention. The objective nature of the data collection (using standardized electronic systems) minimizes the risk of systematic measurement error. | N |
|  | **Risk of bias judgement** |  | Low |
|  | Optional: What is the predicted direction of bias due to measurement of outcomes? |  | Unpredictable |

| **Bias in selection of the reported result** | | | |
| --- | --- | --- | --- |
|  | Is the reported effect estimate likely to be selected, on the basis of the results, from... |  |  |
|  | 7.1. ... multiple outcome *measurements* within the outcome domain? | The study pre-specified multiple outcomes, including antimicrobial consumption (DOT and DDD per 1,000 patient-days), antimicrobial spending, length of stay, and percentage of non-concordance with guidelines. All these outcomes were reported comprehensively, and there is no evidence of selective reporting. | N |
|  | 7.2 ... multiple *analyses* of the intervention-outcome relationship? | The analysis plan was clearly described and involved segmented regression analysis using ARIMA models, along with comparison of the final predicted values with conservative and maximum prediction values. There is no indication that the investigators performed multiple competing analyses with selective reporting. | N |
|  | 7.3 ... different *subgroups*? | Although the study reports outcomes for several endpoints and compares them across the two phases, subgroup analyses (e.g., by antibiotic class or by care setting) were pre-specified and reported in a transparent manner without evidence of selective emphasis. | N |
|  | **Risk of bias judgement** |  | Low |
|  | Optional: What is the predicted direction of bias due to selection of the reported result? |  | Unpredictable |

| **Overall bias** | | | |
| --- | --- | --- | --- |
|  | **Risk of bias judgement** | This retrospective cohort study evaluated the sustained impact of a computer-assisted antimicrobial stewardship intervention on antimicrobial use and length of stay over a five-year period at a large tertiary hospital. Although the study benefits from a large sample size, objective and routinely collected data, and robust analytical methods (segmented regression using ARIMA models) to adjust for secular trends and seasonal variations, its nonrandomized, retrospective design leaves room for residual confounding. In addition, concurrent changes—such as the introduction of generic antimicrobials—were modeled as co-interventions but may not have been fully accounted for. These factors justify an overall risk of bias judgment of Moderate. | Moderate |
|  | Optional: What is the overall predicted direction of bias for this outcome? |  | Unpredictable |
